# Within-job gender pay inequality in 15 countries

**DOI:** 10.1038/s41562-022-01470-z

**Published:** 2022-11-24

**Authors:** Andrew M. Penner, Trond Petersen, Are Skeie Hermansen, Anthony Rainey, István Boza, Marta M. Elvira, Olivier Godechot, Martin Hällsten, Lasse Folke Henriksen, Feng Hou, Aleksandra Kanjuo Mrčela, Joe King, Naomi Kodama, Tali Kristal, Alena Křížková, Zoltán Lippényi, Silvia Maja Melzer, Eunmi Mun, Paula Apascaritei, Dustin Avent-Holt, Nina Bandelj, Gergely Hajdu, Jiwook Jung, Andreja Poje, Halil Sabanci, Mirna Safi, Matthew Soener, Donald Tomaskovic-Devey, Zaibu Tufail

**Affiliations:** 1 Department of Sociology, University of California, Irvine, Irvine, CA USA; 2 Department of Sociology, University of California, Berkeley, Berkeley, CA USA; 3 Department of Sociology and Human Geography, University of Oslo, Oslo, Norway; 4 Swedish Institute for Social Research, Stockholm University, Stockholm, Sweden; 5 Department of Sociology, University of Massachusetts, Amherst, Amherst, MA USA; 6 Centre for Economic and Regional Studies, Budapest, Hungary; 7 Departments of Strategic Management and Managing People in Organizations, IESE Business School, Barcelona, Spain; 8 CRIS-CNRS, Sciences Po, Paris, France; 9 MaxPo, Sciences Po, Paris, France; 10 Department of Sociology, Stockholm University, Stockholm, Sweden; 11 Department of Organization, Copenhagen Business School, Copenhagen, Denmark; 12 Statistics Canada, Ottawa, Ontario Canada; 13 Faculty of Social Sciences, University of Ljubljana, Ljubljana, Slovenia; 14 Faculty of Economics, Meiji Gakuin University, Tokyo, Japan; 15 Department of Sociology, University of Haifa, Haifa, Israel; 16 Institute of Sociology, Czech Academy of Sciences, Prague, Czechia; 17 Department of Sociology, University of Groningen, Groningen, the Netherlands; 18 School of Labor and Employment Relations, University of Illinois, Urbana-Champaign, Urbana-Champaign, IL USA; 19 Department of Social Sciences, Augusta University, Augusta, GA USA; 20 Department of Economics, Vienna University of Economics and Business, Vienna, Austria; 21 Management Department, Frankfurt School of Finance and Management, Frankfurt, Germany; 22 Department of Sociology, University of Illinois, Urbana-Champaign, Urbana-Champaign, IL USA

**Keywords:** Sociology, Social policy

## Abstract

Extant research on the gender pay gap suggests that men and women who do the same work for the same employer receive similar pay, so that processes sorting people into jobs are thought to account for the vast majority of the pay gap. Data that can identify women and men who do the same work for the same employer are rare, and research informing this crucial aspect of gender differences in pay is several decades old and from a limited number of countries. Here, using recent linked employer–employee data from 15 countries, we show that the processes sorting people into different jobs account for substantially less of the gender pay differences than was previously believed and that within-job pay differences remain consequential.

## Abstract (web summary)

Using data from 15 countries, Penner et al. find that women earn less than men who are working for the same employer in the same occupation. These results highlight the continued importance of equal pay for equal work.

## Main

Despite great advances in gender equality, women earn less than men in all advanced industrialized countries. These gender gaps are strongly related to the occupations and establishments in which women and men work. Germinal research highlights that, although there are substantial differences in the overall wages men and women receive, women and men who do the same work for the same employer receive very similar wages^1–3^. The processes involved in sorting women and men into different jobs, and particularly into differentially remunerated male- and female-dominated occupations, are thus viewed as central to understanding gender pay inequality^4–6^.

This understanding of the gender gap has far-reaching policy implications. If there are sizeable differences between the pay that women and men receive when they do the same work for the same employer (that is, within-job inequality), policies mandating equal pay have an important role to play in creating gender equality in the labour market. If, however, differences arise overwhelmingly through sorting women and men into different jobs, policies should focus on the organizational hiring and promotion practices that match people to jobs, as well as on broader societal views regarding whose work is defined as valuable^7–9^.

Most evidence regarding gender pay inequality comes from surveys of individuals that contain occupational data but lack good indicators of firms and jobs. Data that contain detailed occupational information and link individuals to others working for the same employer (that is, linked employer–employee data) are rarely available, so that data that can examine gender differences among those with the same occupation and employer (that is, within-job inequality) are difficult to access. The best evidence on within-job gender pay differences comes from a limited number of countries using linked employer–employee data ranging from 1980 through 1990 to examine within-job gender wage differences^1–3^. In this Article, we contribute to this literature by using linked employer–employee data to provide recent estimates of the levels and change in within-establishment, within-occupation and within-job differences in earnings across 15 countries: Canada, Czechia, Denmark, France, Germany, Hungary, Israel, Japan, the Netherlands, Norway, Slovenia, South Korea, Spain, Sweden and the United States. We show that although much of the gender inequality we observe is accounted for by sorting into establishments, occupations and jobs, within-job gender gaps in earnings remain an important source of differences in all 15 countries. Analyses for the six countries where we can examine the contractual hourly wage rate show that sorting is similarly important for gender differences in wages, suggesting that equal pay policies have an important role to play in creating gender pay equity.

## Results

Our core analyses focus on four sets of ordinary least squares regression models. The first model adjusts only for basic individual-level covariates, and provides our baseline estimate of the overall gender pay gap in each country. In subsequent models, we introduce a series of fixed effects so that we compare women and men working in the same establishment (model 2), the same occupation (model 3) and the same job (that is, occupation–establishment unit; model 4). Comparing the results of these four models enables us to see the degree to which gender differences in pay in any given year are accounted for by sorting across establishments, occupations and occupation–establishment units.

Table 1 presents information on gender differences in earnings in our 15 countries. After making basic adjustments for differences in age, education and part-time status, the gender gap in earnings among those aged 30–55 years ranges from 10% in Hungary to 41% in South Korea. Within-job gender gaps are smaller but still substantial, ranging from 7% in Denmark and France to 26% in Japan. Comparing the results in the first and fourth columns (basic adjustment and within-job), we see that within-job gender differences remain a substantial source of the overall earnings gaps in all of our 15 countries. As is visible in the final column, within-job differences typically account for about half of the overall gender differences that we observe in our countries, ranging from just over a third of the overall gap (Israel) to over nine-tenths of the gender earnings gap in Hungary.

**Table 1:** Gender differences in earnings within establishment, occupation and job

	Year	Basic adjustments	Within:			Proportion within job
			Establishment	Occupation	Job	
Canada	2015	−0.221	−0.172	−0.137	−0.121	0.55
Czechia	2019	−0.280	−0.225	−0.179	−0.123	0.44
Denmark	2015	−0.178	−0.132	−0.107	−0.072	0.40
France	2015	−0.111	−0.108	−0.084	−0.065	0.59
Germany	2015	−0.241	−0.168	−0.206	−0.130	0.54
Hungary	2017	−0.099	−0.130	−0.098	−0.095	0.96
Israel	2015	−0.336	−0.197	−0.196	−0.119	0.35
Japan	2013	−0.350	−0.328	−0.304	−0.257	0.73
The Netherlands	2014	−0.202	−0.146	−0.111	−0.075	0.37
Norway	2018	−0.206	−0.128	−0.120	−0.086	0.42
Slovenia	2015	−0.190	−0.169	−0.157	−0.140	0.74
South Korea	2012	−0.406	−0.244	−0.335	−0.188	0.46
Spain	2017	−0.158	−0.176	−0.164	−0.121	0.77
Sweden	2018	−0.175	−0.118	−0.093	−0.076	0.43
United States	2015	−0.296	−0.214	−0.202	−0.141	0.48

Note: Each estimate represents the coefficient from a separate model estimating the difference between the logged earnings of women and men ages 30–55 years, with negative coefficients indicating that women earn less than men. Following standard conventions, we interpret these coefficients as the relative difference between the average female and male earnings, but more formally they indicate the difference in relative geometric means for unlogged earnings (which is the absolute difference in the arithmetic means of logged earnings). The ‘basic adjustment’ column reports differences from a model that controls for age, age-squared, education and full-time versus part-time status, except in cases where a country is missing a particular measure. Subsequent models provide estimates of within-establishment, within-occupation and within-job (occupation–establishment units) gender differences by introducing fixed effects for establishment, occupation and occupation–establishment units. The final column reports the proportion of the gender difference from the first column (with only basic adjustments) that remains when we compare women and men who are working in the same occupations and establishments. The country-specific information about each measure is summarized in Table 2, and details are provided in country-specific descriptions in the Supplement. *P* < 0.001 for all coefficients. *P* values and confidence intervals are reported in Supplementary Table 1.

The results in the second and third columns of Table 1 report within-establishment and within-occupation gender differences in earnings. Comparing these columns with the results with only basic adjustments highlights the role of sorting into establishments and occupations in creating gender pay differences. Where previous research^1–3^ found that sorting into occupations is substantially more important for gender inequality than sorting into establishments, we find evidence that sorting into both occupations and establishments plays an important role in producing gender differences. Our findings thus not only underscore the salience of within-job differences, but also document the importance of processes that differentially sort women and men into high-paying establishments and occupations.

**Fig. 1:**
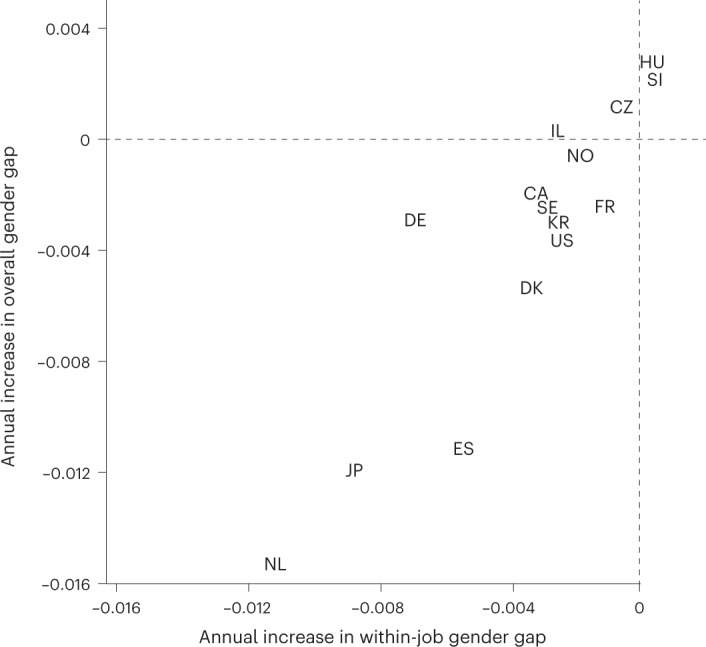
Annual change in overall and within-job gender pay gaps. CA, Canada; CZ, Czechia; DK, Denmark; DE, Germany; ES, Spain; FR, France; HU, Hungary; IL, Israel; JP, Japan; KR, South Korea; NL, the Netherlands; NO, Norway; SI, Slovenia; SE, Sweden; US, United States. The *y* axis represents the average annual change in the overall gender gap in earnings (accounting only for basic adjustments, and corresponding to the first column of results in Table 1), and the *x* axis reports the average annual change in the within-job gender gap in earnings (corresponding to the fourth column of results in Table 1). Larger positive numbers correspond to larger increases in the gender earnings gap across years, while negative numbers correspond to decreases in the gap. We use data from approximately 10 years in each country, beginning in 2005 where possible and continuing through the most recent year available (for information on the most recent year available to us in each country, see Table 1). In three countries (the Netherlands, South Korea and Spain), we do not have data from 2005 and so use 2006 as our initial year. See the tables presented in Supplementary Information for the underlying coefficients reporting gender differences for each year. Supplementary figures depict country-specific trends for overall, within-establishment, within-occupation and within-occupation–establishment gender differences in earnings for each country.

Figure 1 depicts how the within-job and overall gender gaps have changed from 2005 to our most recent year of data (for most countries this represents approximately 10 years; for information on the most recent year that we have data from each country, see Table 1). The *x* axis plots the average annual change in the within-job gender gap for each country, and the *y* axis plots each country’s average annual change in overall gender gap over this period. In most countries, both the overall gender gap and the within-job gender gap have fallen over time. However, this is not the case in the three Central and Eastern European countries. In Czechia, within-job gender differences decline, but overall gender differences in earnings increase, suggesting that gender differences in earnings in Czechia are increasingly due to processes sorting women and men into different jobs. Gender differences also increase in Hungary and Slovenia, where the increase is due not only to sorting processes, but also to an increase in within-job gender gaps. Of particular note, none of our 15 countries exhibits a decrease in the overall gender earnings gap coupled with an increase in within-job gender earnings gaps (as would be the case if egalitarian sorting processes counteracted rising within-job inequality); this suggests that the processes sorting women and men into different jobs are rarely gender egalitarian.

## Discussion

Given the rapid expansion of women’s rights around the world, one might expect uniform improvement in women’s pay via both reduced sorting into different jobs and lower levels of within-job inequality. The empirical record is more mixed, with nearly universal improvements in education and labour force participation, continued and sometimes even increased segregation, and little information on what happens within jobs^10^.

Our analyses of linked employer–employee data from 15 countries show that currently both within-job differences and sorting into jobs make substantial contributions to gender pay gaps. Interestingly, the trends we document highlight that sorting is increasingly important, and that within-job differences are shrinking in importance in most countries. Thus, while the conclusions drawn by previous research—that sorting accounts for the vast majority of gender differences, and within job inequality is not a substantial concern—may not accurately summarize the current state of gender pay inequality, if the trends we observe hold, they may describe our future. In the current context, however, our findings suggest that policies focusing on equal pay for equal work and policies attending to hiring, promotion and other job-sorting processes are both vital to establishing gender equality in the labour market.

### Limitations

Large-scale comparative analyses contain numerous challenges around data harmonization and ensuring that analytic decisions that are appropriate in some contexts are not problematic in others. Although we sought to ensure that the analyses conducted in each country are comparable, factors like parental leave policies, the availability and prevalence of part-time work, and the relevance of occupations and firms differ across our 15 countries. These differences necessarily mean that the comparisons we make across countries involve comparing contexts with different gender regimes and where paid work is organized very differently. Despite these limitations, we believe that these comparisons are informative, and in our Supplementary Information we report results from analyses where we alter variable definitions, model specifications and sample definitions, showing that the results we present here are remarkably robust.

## Methods

This study uses linked employer–employee data (that is, data that link individual employees to specific employers) from 15 countries to investigate the extent to which the gender pay gap arises from women and men receiving different pay when doing the same work for the same employer (as opposed to from processes sorting women and men into different occupations and establishments). By allowing us to compare individuals to others working for the same employer, the linked employer–employee data that we use provide important insights into inequality. Below we provide information on our modelling strategy for our core analyses, and we summarize the data available in each of our 15 countries in Table 2. More information on the data used for each country and results from country-specific robustness checks are included in Supplementary Information, which also presents country-specific results on changes over time, providing a sense of each country’s trends in gender inequality at the overall, establishment, occupation and job (that is, occupation–establishment) levels.

**Table 2:** Key features of data across countries

	Years	Data source	Establishment measure	Occupation measure	Education measure	Job spells or person-years	Sectors/workers omitted and other irregularities
Canada (*N* = 2,807,745)	2005–2015	Linked census data	Firm	4-Digit NOC	NA	Job spell	NA
Czechia (*N* = 1,533,578)	2002–2019	Registry and sample	Firm by region	4-Digit ISCO	15 categories	Person-year	Small (<10) private sector firms
Denmark (*N* = 1,206,326)	1994–2015	Registry	Establishment	4-Digit ISCO	4 categories	Job spell	NA
France (*N* = 12,650,697)	1993–2015	Registry	Establishment	4-Digit CSP	NA	Job spell	Houseworkers
Germany (*N* = 788,946)	1993–2015	Sample from registry	Establishment	4-Digit ISCO	8 categories	Job spell in sampled firm	Civil servants and self-employed; earnings imputed for top earners
Hungary (*N* = 1,509,651)	2003–2017	Sample from registry	Firm	4-Digit ISCO	3-Category proxy	Primary job	NA
Israel (*N* = 16,750)	2001–2015	Sample from registry	Establishment	2-Digit ISCO	3 categories	Highest-earning job spell	Earnings imputed for top earners
Japan (*N* = 604,497)	1993–2013	Sample	Establishment	Imputed	4 categories	Person-year	Agriculture, forestry, fisheries and public services; small establishments
The Netherlands (*N* = 65,919)	2006–2014	Sample from registry	Establishment	3-Digit ISCO; sample only	8-Category ISCED	Job spell	NA
Norway (*N* = 942,735)	1997–2018	Registry	Establishment	4-Digit ISCO	8-Category ISCED	Highest-earning job spell	NA
Slovenia (*N* = 519,746)	1999–2015	Registry	Firm by region	4-Digit ISCO	7-Category ISCED	Job spell	NA
South Korea (*N* = 480,644)	1982–2012	Sample	Establishment	4-Digit ISCO	5 categories	Person-year	Public sector; part-time workers; self-employed
Spain (*N* = 334,665)	2006–2017	Sample	Establishment	Grupo de cotización	4 categories	Job spell	NA
Sweden (*N* = 1,421,040)	2004–2018	Registry and sample	Establishment	4-Digit ISCO	16 categories	Job spell	NA
United States (*N* = 1,091,000)	2005–2015	Linked census data and sample	Firm	3-Digit SOC; sample only	6 categories	Highest-earning job spell	NA

Note: *N* contains information from the *N* of model 1 from Table 1.

### Models

As noted above, our core analyses focus on four sets of ordinary least squares regression models. Our first model adjusts only for basic individual-level covariates, and provides our baseline estimate of the overall gender pay gap in each country. In subsequent models we compare only women and men who work in the same establishment (model 2), only women and men who work in the same occupation (model 3) and only women and men who work in the same job (that is, occupation-establishment unit; model 4). We estimate these models separately by year for each country, allowing us to examine country-specific trends in these gender differences.

The equations estimated for our core models follow the same general form, using four different specifications:

1 $${{{\mathrm{ln}}}}\,{\mathrm{earnings}}_{it} = \theta _{B,t}x_{it} + \eta _{ft} + \varepsilon _{it},$$

2 $${{{\mathrm{ln}}}}\,{\mathrm{earnings}}_{it} = \theta _{E,t}x_{it} + \eta _{eft} + \varepsilon _{it},$$

3 $${{{\mathrm{ln}}}}\,{\mathrm{earnings}}_{it} = \theta _{O,t}x_{it} + \eta _{oft} + \varepsilon _{it},$$

4 $${{{\mathrm{ln}}}}\,{\mathrm{earnings}}_{it} = \theta _{OE,t}x_{it} + \eta _{oeft} + \varepsilon _{it},$$

where the subscripts represent *i* for individuals (or for each employment spell of an individual, depending on the country), *f* for full-time versus part-time status, *o* for occupations, *e* for establishments and *t* for years. The dependent variable is the logarithm of earnings (ln earnings_*it*_) for individual (or employment spell) *i* in year *t*, and the independent variables are collected in the vector *x*_*it*_, which includes a constant, the gender, age and age-squared of individual *i*, and a series of indicator variables for the education of individual *i* (except in countries where information on education was not available).

To address concerns regarding the comparability of full-time versus part-time workers, we consider full-time versus part-time status a defining characteristic of a job and include this axis in constructing fixed effects for all of our core models. Thus, model 1 includes the term *η*_*ft*_, a fixed effect (that is, indicator variable) for full-time versus part-time work, so that this basic adjustment model adjusts for age, age-squared, education and full-time versus part-time work. Model 2 includes the covariates in *x*_*it*_ (age, age-squared and education), as well as the fixed effects *η*_*eft*_ representing the unique units formed by combining the establishment and full-time versus part-time indicators. Model 2 thus provides estimates of the gender gap obtained from comparing women and men who work in the same establishment; for each establishment it can be thought of as estimating the gender gap separately for full-time workers and part-time workers and then taking a weighted average of these two gender gaps across all establishments. Models 3 and 4 are analogous to model 2, but contain the fixed effects *η*_*oft*_ and *η*_*oeft*_ that refer respectively to the unique units formed by combining full-time versus part-time status with either occupation (*η*_*oft*_) or occupation–establishment units (*η*_*oeft*_). The analytic sample for each model is restricted to gender-integrated fixed effect units. The subscripts to the *θ* parameters indicate that these are different coefficients, pertaining to different levels, basic adjustments (*B*), establishment (*E*), occupation (*O*) and occupation–establishment (*OE*).

We use the natural log of earnings as our dependent variable. Following standard conventions, these coefficients are interpreted as the relative difference between the average female and male earnings, but more formally our estimates refer to the difference in relative geometric means for unlogged earnings (which is the absolute difference in the arithmetic means of logged earnings). For an extended discussion of the interpretation of such coefficients, see Petersen^11^.

Data were analysed using STATA versions 14–17 and SAS version 9.

### Reporting summary

Further information on research design is available in the Nature Research Reporting Summary linked to this article.

## Supplementary information

Supplementary Information Supplementary Discussion, Tables 1–30 and Figs. 1–18.

Reporting Summary

## Data Availability

This paper uses restricted-access data from 15 different countries. As described in Supplementary Information, the data underlying our analyses in each country can be accessed by receiving permissions from the relevant data owners, including Statistics Canada; the Ministry of Labor and Social Affairs of the Czech Republic; Statistics Denmark; the French Comité du Secret Statistique; the German Institute for Employment Research; the Databank of the Centre for Economic and Regional Studies in Hungary; Israel’s Central Bureau of Statistics (CBS); the Japanese Ministry of Health, Labour and Welfare; the Central Bureau of Statistics of the Netherlands; Statistics Norway; the Slovenian Statistical Office; Statistics Korea; the Ministry of Labor, Migration and Social Security of Spain; Statistics Sweden; and the US Census Bureau.
