# Author Correction: Within-job gender pay inequality in 15 countries

**DOI:** 10.1038/s41562-023-01523-x

**Published:** 2023-01-31

**Authors:** Andrew M. Penner, Trond Petersen, Are Skeie Hermansen, Anthony Rainey, István Boza, Marta M. Elvira, Olivier Godechot, Martin Hällsten, Lasse Folke Henriksen, Feng Hou, Aleksandra Kanjuo Mrčela, Joe King, Naomi Kodama, Tali Kristal, Alena Křížková, Zoltán Lippényi, Silvia Maja Melzer, Eunmi Mun, Paula Apascaritei, Dustin Avent-Holt, Nina Bandelj, Gergely Hajdu, Jiwook Jung, Andreja Poje, Halil Sabanci, Mirna Safi, Matthew Soener, Donald Tomaskovic-Devey, Zaibu Tufail

**Affiliations:** 1 Department of Sociology, University of California, Irvine, Irvine, CA USA; 2 Department of Sociology, University of California, Berkeley, Berkeley, CA USA; 3 Department of Sociology and Human Geography, University of Oslo, Oslo, Norway; 4 Swedish Institute for Social Research, Stockholm University, Stockholm, Sweden; 5 Department of Sociology, University of Massachusetts, Amherst, Amherst, MA USA; 6 Centre for Economic and Regional Studies, Budapest, Hungary; 7 Departments of Strategic Management and Managing People in Organizations, IESE Business School, Barcelona, Spain; 8 CRIS-CNRS, Sciences Po, Paris, France; 9 MaxPo, Sciences Po, Paris, France; 10 Department of Sociology, Stockholm University, Stockholm, Sweden; 11 Department of Organization, Copenhagen Business School, Copenhagen, Denmark; 12 Statistics Canada, Ottawa, Ontario Canada; 13 Faculty of Social Sciences, University of Ljubljana, Ljubljana, Slovenia; 14 Faculty of Economics, Meiji Gakuin University, Tokyo, Japan; 15 Department of Sociology, University of Haifa, Haifa, Israel; 16 Institute of Sociology, Czech Academy of Sciences, Prague, Czechia; 17 Department of Sociology, University of Groningen, Groningen, the Netherlands; 18 School of Labor and Employment Relations, University of Illinois, Urbana-Champaign, Urbana-Champaign, IL USA; 19 Department of Social Sciences, Augusta University, Augusta, GA USA; 20 Department of Economics, Vienna University of Economics and Business, Vienna, Austria; 21 Management Department, Frankfurt School of Finance and Management, Frankfurt, Germany; 22 Department of Sociology, University of Illinois, Urbana-Champaign, Urbana-Champaign, IL USA

**Keywords:** Sociology, Social policy

Correction to: *Nature Human Behaviour* 10.1038/s41562-022-01470-z. Published online 24 November 2022.

In the version of this article initially published, the Acknowledgements information for Aleksandra Kanjuo Mrčela did not include thanks for support from the Slovenian Research Agency (ARRS) under grant no. P5-0193. The error has been corrected in the HTML and PDF versions of the article.

