# Supplementary material for: Within-job gender pay inequality in 15 countries
Source: Nat Hum Behav. 2022 Nov 24;7(2):184–9. doi: 10.1038/s41562-022-01470-z (PMC9957724; doi:10.1038/s41562-022-01470-z)
Supplement: Supplementary file 1 — Supplementary Discussion, Tables 1–30 and Figs. 1–18. [file 41562_2022_1470_MOESM1_ESM.pdf]

nature human behaviour

Article

https://doi.org/10.1038/s41562-022-01470-z

# Within-job gender pay inequality in 15 countries

In the format provided by the authors and unedited

## Supplementary Information for

# Within-Job Gender Pay Inequality in 15 Countries

Andrew Penner, Trond Petersen, Are Skeie Hermansen, Anthony Rainey, István Boza, Marta M. Elvira, Olivier Godechot, Martin Hällsten, Lasse Folke Henriksen, Feng Hou, Aleksandra Kanjuo Mrčela, Joe King, Naomi Kodama, Tali Kristal, Alena Křížková, Zoltán Lippényi, Silvia Maja Melzer, Eunmi Mun, Paula Apascaritei, Dustin Avent-Holt, Nina Bandelj, Gergely Hajdu, Jiwook Jung, Andreja Poje, Halil Sabanci, Mirna Safi, Matthew Soener, Donald Tomaskovic-Devey, Zaibu Tufail

**Corresponding author: Andrew Penner**
**E-mail: penner@uci.edu**

**This PDF file includes:**

- Supplementary text
- Supplementary Figures 1 to 18
- Supplementary Tables 1 to 30
- References to SI reference citations

## Contents

Appendix A. Overview and Supplementary Information for Table 1 .......... 5
Appendix B. Analytical Decision and Robustness Checks .......... 6
  Wages and Earnings .......... 6
  Defining Jobs and Addressing Part-time Work .......... 8
  Education .......... 11
  Establishments and Firms .......... 12
  Coverage of Employees within Establishments .......... 12
  Age .......... 14
  Persons vs. Employment Spells .......... 15
Appendix D. Country-specific Data Descriptions and Trends .......... 17
  Canada .......... 17
  Czechia .......... 18
  Denmark .......... 20
  France .......... 22
  Germany .......... 26
  Hungary .......... 32
  Israel .......... 34
  Japan .......... 36
  Netherlands .......... 38
  Norway .......... 40
  Slovenia .......... 43
  South Korea .......... 45
  Spain .......... 47
  Sweden .......... 50
  United States .......... 53
Supplement References .......... 56

## List of Figures

Supplementary Figure 1. Trends in Canadian Gender Differences in Earnings ........ 18

Supplementary Figure 2. Trends in Czech Gender Differences in Earnings ........ 20

Supplementary Figure 3. Trends in Danish Gender Differences in Earnings ........ 22

Supplementary Figure 4. Trends in French Gender Differences in Earnings ........ 25

Supplementary Figure 5. Trends in French Gender Differences in Earnings(from EDP-DADS 1% Panel w/out Education in the Model) ........ 26

Supplementary Figure 6. Trends in German Gender Differences in Earnings ........ 31

Supplementary Figure 7. Trends in East German Gender Differences in Earnings ........ 31

Supplementary Figure 8. Trends in West German Gender Differences in Earnings ........ 32

Supplementary Figure 9. Trends in Hungarian Gender Differences in Earnings ........ 34

Supplementary Figure 10. Trends in Israeli Gender Differences in Earnings ........ 36

Supplementary Figure 11. Trends in Japanese Gender Differences in Earnings ........ 38

Supplementary Figure 12. Trends in Dutch Gender Differences in Earnings ........ 40

Supplementary Figure 13. Trends in Norwegian Gender Differences in Earnings ........ 43

Supplementary Figure 14. Trends in Slovenian Gender Differences in Earnings ........ 45

Supplementary Figure 15. Trends in South Korean Gender Differences in Earnings ........ 47

Supplementary Figure 16. Trends in Spanish Gender Differences in Earnings ........ 50

Supplementary Figure 17. Trends in Swedish Gender Differences in Earnings ........ 53

Supplementary Figure 18. Trends in US Gender Differences in Earnings ........ 55

## List of Tables

Supplementary Table 1. Confidence intervals (95%) and p-values (two-tailed) for coefficients reported in Table 1. ........ 5

Supplementary Table 2. Gender Differences in Wages and Hourly Earnings within Occupation, Establishment, and Job ........ 7

Supplementary Table 3. Gender Differences in Earnings using Coarsened (One-Digit) Occupational Categories ........ 9

Supplementary Table 4. Gender Differences in Earnings at Different Levels in Models that Include Part-time Status as an Independent Control ........ 10

Supplementary Table 5. Gender Differences in Earnings at Different Levels in Models That Do Not Distinguish Between Part-Time and Full-Time Work ........ 10

Supplementary Table 6. Gender Differences in Earnings at Different Levels, on Sample with Education 11
Supplementary Table 7. Gender Differences in Earnings at Different Levels, Without Controls for Age and Education 11
Supplementary Table 8. Gender Differences in Earnings at Different Levels for Gender-Integrated Job Units 13
Supplementary Table 9. Gender Differences in Earnings at Different Levels with Alternate Age Cutoffs for the Sample 14
Supplementary Table 10. Gender Differences in Earnings at Different Levels, Using Persons as Unit of Analysis 15
Supplementary Table 11. Canadian Trends in Gender Differences in Earnings 18
Supplementary Table 12. Czech Trends in Gender Differences in Earnings 19
Supplementary Table 13. Danish Trends in Gender Differences in Earnings 21
Supplementary Table 14. French Trends in Gender Differences in Earnings from Social Security Data 23
Supplementary Table 15. French Trends in Gender Differences in Earnings from 1% Panel Data, With and Without Education 24
Supplementary Table 16. German Trends in Gender Differences in Earnings 28
Supplementary Table 17. East German Trends in Gender Differences in Earnings 29
Supplementary Table 18. West German Trends in Gender Differences in Earnings 30
Supplementary Table 19. Hungarian Trends in Gender Differences in Earnings 33
Supplementary Table 20. Israeli Trends in Gender Differences in Earnings 35
Supplementary Table 21. Japanese Trends in Gender Differences in Earnings 37
Supplementary Table 22. Dutch Trends in Gender Differences in Earnings 40
Supplementary Table 23. Norwegian Trends in Gender Differences in Earnings 42
Supplementary Table 24. Slovenian Trends in Gender Differences in Earnings 44
Supplementary Table 25. South Korean Trends in Gender Differences in Earnings 46
Supplementary Table 26. Spanish Trends in Gender Differences in Earnings 49
Supplementary Table 27. Swedish Trends in Gender Differences in Earnings 51
Supplementary Table 28. Swedish Trends in Gender Differences in Earnings using Whole Labor Market LISA Data 52
Supplementary Table 29. US Trends in Gender Differences in Earnings 54
Supplementary Table 30. US Trends in Gender Differences in Earnings using Full W2 Sample 55

## Appendix A. Overview and Supplementary Information for Table 1

This supplement provides additional information about data utilized in each country and reports results from country-specific supplementary analyses. Supplementary Table 1 also provides p-values and confidence intervals for the results reported in Table 1; as noted in the manuscript, all p-values are less than .001.

We first provide an overview of key decisions that informed our analyses, and report results from supplemental analyses where we alter variable definitions, model specifications, or sample definitions (Appendix B). We then discuss the differences in analytical decisions between our approach and previous work using Norwegian and Swedish data, highlighting the choices that produce the divergence in findings (Appendix C). Finally, in Appendix D we provide country-specific details about data and report country-specific trends in gender pay gaps at the four levels reported in Table 1: 1) including controls for basic covariates; 2) within-establishment; 3) within-occupation; and 4) within-job.

**Supplementary Table 1.** Confidence intervals (95%) and p-values (two-tailed) for coefficients reported in Table 1.

| | | Basic Adjustments | Within: | | |
|---|---|---|---|---|---|
| | | | Est | Occ | Job |
| Canada | Coefficient | -.221 | -.172 | -.137 | -.121 |
| | Confidence interval | [-0.223, -0.218] | [-0.174, -0.169] | [-0.140, -0.135] | [-0.125, -0.118] |
| | P-value | p<.0001 | p<.0001 | p<.0001 | p<.0001 |
| Czechia | Coefficient | -.280 | -.225 | -.179 | -.123 |
| | Confidence interval | [-0.284, -0.275] | [-0.229, -0.220] | [-0.185, -0.173] | [-0.128, -0.117] |
| | P-value | p<.0001 | p<.0001 | p<.0001 | p<.0001 |
| Denmark | Coefficient | -.178 | -.132 | -.107 | -.072 |
| | Confidence interval | [-0.179, -0.177] | [-0.134, -0.131] | [-0.108, -0.105] | [-0.073, -0.071] |
| | P-value | p<.0001 | p<.0001 | p<.0001 | p<.0001 |
| France | Coefficient | -.111 | -.108 | -.084 | -.065 |
| | Confidence interval | [-0.111, -0.110] | [-0.109, -0.107] | [-0.084, -0.083] | [-0.065, -0.064] |
| | P-value | p<.0001 | p<.0001 | p<.0001 | p<.0001 |
| Germany | Coefficient | -.241 | -.168 | -.206 | -.130 |
| | Confidence interval | [-0.250, -0.232] | [-0.172, -0.164] | [-0.215, -0.198] | [-0.134, -0.125] |
| | P-value | p<.0001 | p<.0001 | p<.0001 | p<.0001 |
| Hungary | Coefficient | -.099 | -.130 | -.098 | -.095 |
| | Confidence interval | [-0.101, -0.097] | [-0.132, -0.128] | [-0.100, -0.096] | [-0.097, -0.093] |
| | P-value | p<.0001 | p<.0001 | p<.0001 | p<.0001 |
| Israel | Coefficient | -.336 | -.197 | -.196 | -.119 |
| | Confidence interval | [-0.354, -0.318] | [-0.218, -0.177] | [-0.215, -0.176] | [-0.149, -0.089] |
| | P-value | p<.0001 | p<.0001 | p<.0001 | p<.0001 |
| Japan | Coefficient | -.350 | -.328 | -.304 | -.257 |
| | Confidence interval | [-0.356, -0.345] | [-0.333, -0.322] | [-0.310, -0.298] | [-0.263, -0.251] |

| | | | | | |
|---|---|---|---|---|---|
| | P-value | p<.0001 | p<.0001 | p<.0001 | p<.0001 |
| Netherlands | Coefficient | -.202 | -.146 | -.111 | -.075 |
| | Confidence interval | [-0.218, -0.187] | [-0.163, -0.128] | [-0.128, -0.094] | [-0.102, -0.049] |
| | P-value | p<.0001 | p<.0001 | p<.0001 | p<.0001 |
| Norway | Coefficient | -.206 | -.128 | -.120 | -.086 |
| | Confidence interval | [-0.207, -0.204] | [-0.130, -0.127] | [-0.121, -0.118] | [-0.087, -0.084] |
| | P-value | p<.0001 | p<.0001 | p<.0001 | p<.0001 |
| Slovenia | Coefficient | -.190 | -.169 | -.157 | -.140 |
| | Confidence interval | [-0.192, -0.188] | [-0.171, -0.166] | [-0.160, -0.155] | [-0.143, -0.137] |
| | P-value | p<.0001 | p<.0001 | p<.0001 | p<.0001 |
| South Korea | Coefficient | -.406 | -.244 | -.335 | -.188 |
| | Confidence interval | [-0.412, -0.400] | [-0.249, -0.240] | [-0.341, -0.329] | [-0.193, -0.183] |
| | P-value | p<.0001 | p<.0001 | p<.0001 | p<.0001 |
| Spain | Coefficient | -.158 | -.176 | -.164 | -.121 |
| | Confidence interval | [-0.164, -0.153] | [-0.182, -0.169] | [-0.170, -0.159] | [-0.129, -0.114] |
| | P-value | p<.0001 | p<.0001 | p<.0001 | p<.0001 |
| Sweden | Coefficient | -.175 | -.118 | -.093 | -.076 |
| | Confidence interval | [-0.180, -0.170] | [-0.122, -0.114] | [-0.098, -0.087] | [-0.079, -0.072] |
| | P-value | p<.0001 | p<.0001 | p<.0001 | p<.0001 |
| United States | Coefficient | -.296 | -.214 | -.202 | -.141 |
| | Confidence interval | [-0.435, -0.157] | [-0.222, -0.206] | [-0.220, -0.184] | [-0.147, -0.135] |
| | P-value | p<.0001 | p<.0001 | p<.0001 | p<.0001 |

## Appendix B. Analytical Decision and Robustness Checks

Below we highlight some of the common challenges and key decisions that informed our analyses, and report results from analyses where we alter variable definitions, model specifications, or sample definitions. We sought to make decisions that facilitated comparisons across our 15 countries, but also deferred to country leads on analytic decisions (e.g., educational categories, defining part-time work, and unreasonably low hours) to ensure that they made sense in that context.

***Wages and Earnings***. The distinction between wages and earnings is important: analyses of wages provide insight into inequality from the employer's perspective (the price employers pay for a unit of a particular employee's time); analyses of earnings capture the perspective of what employees receive, including potential differences in overtime, performance bonuses, and other components affecting take home pay, as well as how work contributes to employees' broader economic well-being. Research establishing the importance of sorting, and the relative unimportance of within-job differences (e.g., *1-3*) has focused on wages. The ability to isolate contractual hourly wage varies widely across countries, and as such in our primary analyses we focus on gender differences in gross earnings (controlling for full- and part-time work), as we have information on gross earnings in all 15 countries.

Although it has been widely assumed that sorting processes play a similar role in structuring differences in earnings and wages, this has not been empirically examined. As such, Supplementary Table 2 presents results for wages for the subset of countries where we can examine hourly wages. In the countries listed in Panel A we are able to calculate the hourly wage on contractual hours. In the countries listed in Panel B, we are unable to do so and use country-specific imputed measures of wages. In most of the countries in Panel B (Israel, Japan, Spain, and the United States), we use a measure of hourly earnings, as we are unable to separate overtime and regular earnings. In France, however, we are able to use administrative information on hours worked and the national overtime wage multipliers to more closely approximate hourly wage. As we cannot account for firm- and sector-specific overtime multipliers in France, to the degree that those working overtime are in workplaces covered by more generous agreements, our measure will diverge somewhat from a measure of hourly wage on contractual hours.

**Supplementary Table 2.** Gender Differences in Wages and Hourly Earnings within Occupation, Establishment, and Job

| | Year | Basic adj. | Fixed Effect for: | | | Proportion within job |
|---|---|---|---|---|---|---|
| | | | Est | Occ | Occ-Est | |
| **Panel A: Hourly Wage on Contractual Hours** | | | | | | |
| Czechia | 2019 | -.232 | -.184 | -.151 | -.098 | .42 |
| Denmark | 2015 | -.152 | -.119 | -.085 | -.063 | .41 |
| Netherlands | 2014 | -.088 | -.078 | -.075 | -.044 | .50 |
| Norway | 2018 | -.137 | -.080 | -.076 | -.046 | .34 |
| South Korea | 2012 | -.277 | -.218 | -.254 | -.175 | .63 |
| Sweden | 2018 | -.125 | -.077 | -.051 | -.035 | .28 |
| **Panel B: Hourly Earnings** | | | | | | |
| France | 2015 | -.115 | -.117 | -.095 | -.071 | .62 |
| Hungary | 2017 | -.108 | -.118 | -.097 | -.088 | .81 |
| Israel | 2015 | -.250 | -.136 | -.134 | -.087 | .35 |
| Japan | 2013 | -.320 | -.299 | -.269 | -.222 | .69 |
| Spain | 2017 | -.170 | -.153 | -.160 | -.101 | .59 |
| United States | 2015 | -.159 | -.122 | -.106 | -.085 | .53 |

Comparing Table 1 and Supplementary Table 2, we see that the degree to which sorting accounts for the gender gap is similar across earnings and wages. The gender differences we observe in wages are typically smaller than those we observe in earnings, but as with earnings, we find that around half of the gender wage gap is typically attributable to within-job differences, with sorting into jobs accounting for the other half. Our results for wages suggest that even in contexts like Norway and Sweden where gender wage differences were historically overwhelmingly due to sorting (*2*, *3*), within-job wage differences are now an important factor.

***Defining Jobs and Addressing Part-time Work***. We follow standard conventions in this literature in referring to the within occupation-establishment unit estimate as a "within-job" estimate (1-4). Jobs are often conceptualized as falling at the intersection of occupations and establishments, where individuals are hired to do "particular task[s] within a particular work group in a particular company or establishment" (5, pg. 9). As noted by Petersen et al. (3): "There is a question as to what is the appropriate level of detail for occupational or job titles, because if they get too detailed, the titles may just be indicators of wage levels rather than distinguishing the content of work performed" (pg. 203). We use a four-digit occupational classification scheme in ten of our 15 countries (Canada, Czechia, Denmark, France, Germany, Hungary, Norway, Slovenia, South Korea, and Sweden); in other countries we used a less precise measure because we only have data on a sample of individuals within a particular firm (Israel, the Netherlands, and the United States) or because of other data constraints (Japan and Spain).

To ensure that differences in the granularity of our occupational measurements are not problematic we also estimate models using coarsened one-digit occupational codes. Results from these models are reported in Supplementary Table 3. The final column of Supplementary Table 3 reports the degree to which sorting into jobs defined by four-digit occupations can be accounted for by sorting into jobs that are defined by one-digit occupations. We see that in nine of the 12 countries where we are able to estimate these models, sorting into jobs defined by one-digit occupational codes accounts for over 70 percent of the reductions in the gender gap that we observe when we account for sorting into jobs defined using finer-grained (e.g., four-digit) occupational codes.

Although in many countries it is common to focus only on full-time workers and omit part-time workers, we instead include all workers (except in South Korea, where our sample includes only full-time workers). As part-time work is differentially common across countries, focusing only on full-time workers could induce country-level differences that are a function of sorting into full- versus part-time work. Given the important differences between full- and part-time work, we believe that it is important not to treat full- and part-time workers as having the same job. As such, we conceptualize full- versus part-time status as an axis (along with occupation and establishment) that defines a job. Thus, as described below, our four main specifications include the following fixed effects: part-time status (Model 1), establishment by part-time status units (Model 2), occupation by part-time status units (Model 3), and occupation by establishment by part-time status units (Model 4). This means that we only compare part-time workers to other part-time workers, and full-time workers to other full-time workers, and in contexts where we are comparing workers within a given establishment, we compare part-time workers in that establishment to each other, and full-time workers in that establishment to each other, and then take the weighted average of the gender differences from these comparisons. If we instead include full- versus part-time status as an additive control variable in the model (i.e., do not interact it with fixed effects for occupation, establishment, or job units), we find largely similar results (see Supplementary Table 4).

**Supplementary Table 3.** Gender Differences in Earnings using Coarsened (One-Digit) Occupational Categories

| | Year | Basic Adj. | Fixed Effect for: Coarsened Occ | | | Fixed Effect for: Results from Table 1 | | Proportion within Job (Coarsened) | Sorting into Jobs (Coars-ened) | Proportion within Job (Table 1 ) | Sorting into Jobs (Table 1) | Coarsened Sorting / Ta-ble 1 Sorting |
|---|---|---|---|---|---|---|---|---|---|---|---|---|
| | | | Est | Occ | Occ-Est | Occ | Occ-Est | | | | | |
| Canada | 2015 | -.221 | -.172 | -.209 | -.170 | -.137 | -.121 | .77 | .23 | .55 | .45 | .51 |
| Czechia | 2019 | -.280 | -.225 | -.241 | -.179 | -.179 | -.123 | .64 | .36 | .44 | .56 | .64 |
| Denmark | 2015 | -.178 | -.132 | -.159 | -.095 | -.107 | -.072 | .53 | .47 | .40 | .60 | .78 |
| France | 2015 | -.111 | -.108 | -.107 | -.078 | -.084 | -.065 | .70 | .30 | .59 | .41 | .72 |
| Germany | 2015 | -.241 | -.168 | -.277 | -.142 | -.206 | -.130 | .59 | .41 | .54 | .46 | .89 |
| Hungary | 2017 | -.099 | -.130 | -.141 | -.114 | -.081 | -.091 | 1.00 | .00 | .92 | .08 | .00 |
| Israel | 2015 | -.336 | -.197 | -.297 | -.162 | -.196 | -.119 | .48 | .52 | .35 | .65 | .80 |
| Netherlands | 2014 | -.202 | -.146 | -.166 | -.103 | -.111 | -.075 | .51 | .49 | .37 | .63 | .78 |
| Norway | 2018 | -.206 | -.128 | -.186 | -.102 | -.120 | -.086 | .50 | .50 | .42 | .58 | .87 |
| Slovenia | 2015 | -.190 | -.169 | -.178 | -.137 | -.157 | -.140 | .72 | .28 | .74 | .26 | 1.00 |
| South Korea | 2012 | -.406 | -.244 | -.401 | -.215 | -.335 | -.188 | .53 | .47 | .46 | .54 | .88 |
| Sweden | 2018 | -.175 | -.118 | -.144 | -.093 | -.093 | -.076 | .53 | .47 | .43 | .57 | .83 |

Note: Within-occupation and within-job models use either a one-digit coarsened occupational code or the standard occupational coding system used in Table 1 and described in Table 2. The final column of this table reports the degree to which the reductions in the gender gap associated with sorting into jobs defined by finer-grained (e.g., four-digit) occupations can be accounted for by sorting into jobs defined by one-digit occupational categories. In Hungary the within job gender gap (where job is defined as the intersection of one digit occupation and establishment) is larger than the gender gap with basic adjustments only, so we report that the one-digit occupational category based job definition does not account for any of the sorting into finer-grained occupational category based jobs. In Slovenia, within-job gender inequality is smaller when we define jobs using one-digit occupational categories than when we define jobs using finer-grained occupational categories, so we note that the one-digit occupation version accounts for all of the finer-grained based job sorting.

**Supplementary Table 4.** Gender Differences in Earnings at Different Levels in Models that Include Part-time Status as an Independent Control

| | Year | Basic Adj. | Fixed Effect for: | | | Proportion within job |
|---|---|---|---|---|---|---|
| | | | Est | Occ | Occ-Est | |
| Canada | 2015 | -.221 | -.165 | -.134 | -.117 | .53 |
| Czechia | 2019 | -.280 | -.221 | -.180 | -.120 | .43 |
| Denmark | 2015 | -.178 | -.133 | -.108 | -.073 | .41 |
| France | 2015 | -.111 | -.108 | -.082 | -.061 | .55 |
| Germany | 2015 | -.241 | -.175 | -.213 | -.140 | .58 |
| Hungary | 2017 | -.099 | -.125 | -.098 | -.093 | .94 |
| Israel | 2015 | -.336 | -.202 | -.196 | -.133 | .40 |
| Japan | 2013 | -.350 | -.319 | -.299 | -.250 | .71 |
| Netherlands | 2014 | -.202 | -.150 | -.125 | -.066 | .33 |
| Norway | 2018 | -.206 | -.131 | -.124 | -.086 | .42 |
| Slovenia | 2015 | -.190 | -.169 | -.157 | -.140 | .74 |
| Spain | 2017 | -.158 | -.172 | -.169 | -.118 | .75 |
| Sweden | 2018 | -.175 | -.120 | -.095 | -.077 | .44 |

**Supplementary Table 5.** Gender Differences in Earnings at Different Levels in Models That Do Not Distinguish Between Part-Time and Full-Time Work

| | Year | Basic Adj. | Fixed Effect for: | | | Proportion within job |
|---|---|---|---|---|---|---|
| | | | Est | Occ | Occ-Est | |
| Canada | 2015 | -.516 | -.322 | -.303 | -.201 | .39 |
| Czechia | 2019 | -.332 | -.246 | -.203 | -.139 | .42 |
| Denmark | 2015 | -.183 | -.133 | -.108 | -.072 | .39 |
| France | 2015 | -.196 | -.156 | -.140 | -.102 | .52 |
| Germany | 2015 | -.437 | -.304 | -.338 | -.242 | .55 |
| Hungary | 2017 | -.131 | -.145 | -.110 | -.105 | .80 |
| Israel | 2015 | -.452 | -.245 | -.245 | -.162 | .36 |
| Japan | 2013 | -.717 | -.524 | -.570 | -.407 | .57 |
| Netherlands | 2014 | -.585 | -.365 | -.396 | -.226 | .39 |
| Norway | 2018 | -.306 | -.169 | -.161 | -.107 | .35 |
| Slovenia | 2015 | -.211 | -.180 | -.172 | -.148 | .70 |
| Spain | 2017 | -.376 | -.220 | -.345 | -.148 | .39 |
| Sweden | 2018 | -.221 | -.139 | -.110 | -.088 | .40 |

To highlight the importance of taking part-time status into account, Supplementary Table 5 provides results from models that do not account for full- versus part-time status; these models compare all workers to each other regardless of full- versus part-time status. Where the results in Supplementary Table 4 show that accounting for part-time status as a separate additive factor produces similar results to those in Table 1 (where part-time status is considered a defining characteristic of a job), the gender gaps in Supplementary Table 5 (where no adjustments for part-time status are

made) tend to be substantially larger. In Canada, Japan, the Netherlands, and Spain the basic adjusted gender gap is over twice as high without the part-time adjustment, and in the Netherlands the within-job gender pay gap without the part-time adjustment is also more than double.

***Education***. We control for education to account for basic differences in human capital wherever possible, using indicator variables that reflect each country's educational system (see the country-specific descriptions in the Supplemental Material for additional information regarding education). However, in Canada and France we only have information on education for a sub-sample of individuals. Rather than include only individuals with this information, we omit education from our primary models in these countries. Supplementary Table 6 estimates results on the sub-sample including education for Canada and France. In Hungary, we use a proxy for education based on the educational qualifications required for an individual's current and previous jobs. We estimate models without controls for education and age, which we report in Supplementary Table 7. Comparing results across Tables 1 and Supplementary Tables 6 and 7 suggests that gender earnings gaps tend to be larger in models that account for education than they are in those that do not, reflecting the higher educational levels of women in most countries. Importantly these differences are relatively small when comparing women and men working in the same occupation and establishment.

**Supplementary Table 6.** Gender Differences in Earnings at Different Levels, on Sample with Education

| | Year | Basic Adj. | Fixed Effect for: | | |
|---|---|---|---|---|---|
| | | | Est | Occ | Occ-Est |
| Canada | 2015 | -.189 | -.170 | -.136 | -.122 |
| France | 2015 | -.169 | -.118 | -.096 | -.083 |

**Supplementary Table 7.** Gender Differences in Earnings at Different Levels, Without Controls for Age and Education

| | Year | Basic Adj. | Fixed Effect for: | | | Proportion within job |
|---|---|---|---|---|---|---|
| | | | Est | Occ | Occ-Est | |
| Canada | 2015 | -.189 | -.170 | -.136 | -.122 | .65 |
| Czechia | 2019 | -.236 | -.209 | -.165 | -.109 | .46 |
| Denmark | 2015 | -.140 | -.131 | -.096 | -.068 | .49 |
| France | 2015 | -.109 | -.110 | -.082 | -.064 | .59 |
| Germany | 2015 | -.231 | -.178 | -.215 | -.133 | .58 |
| Hungary | 2017 | -.083 | -.152 | -.099 | -.090 | 1.08 |
| Israel | 2015 | -.281 | -.185 | -.175 | -.096 | .34 |
| Japan | 2013 | -.379 | -.351 | -.312 | -.258 | .68 |
| Netherlands | 2014 | -.175 | -.156 | -.112 | -.082 | .47 |
| Norway | 2018 | -.162 | -.124 | -.114 | -.085 | .52 |
| Slovenia | 2015 | -.069 | -.123 | -.127 | -.117 | 1.70 |

| | | | | | | |
|---|---|---|---|---|---|---|
| South Korea | 2012 | -.473 | -.301 | -.378 | -.228 | .48 |
| Spain | 2017 | -.058 | -.160 | -.153 | -.118 | 2.03 |
| Sweden | 2018 | -.128 | -.110 | -.084 | -.071 | .55 |
| United States | 2015 | -.251 | -.213 | -.209 | -.147 | .59 |

Note: In Canada and France the models reported in Table 1 do not include education, so the difference between the results in this table and Table 1 is the inclusion of age as a covariate. In the United States, the models reported in this table include age, but do not include education.

***Establishments and Firms***. Wherever possible we use data on establishments, which allow us to compare individuals working for the same firm at the same physical location. However, in some countries we only have information at the firm level. Although firm and establishment are identical for single-establishment firms, firms can include information from multiple establishments. We use firms as our proxy for establishments in Hungary and the United States, and we use firm-by-region units as our proxy for establishments in Canada, Czechia and Slovenia.

***Coverage of Employees within Establishments***. To compare the pay of women and men in the same occupation and establishment, it is important to have good coverage of employees within establishments. Administrative records that cover all individuals in the economy provide such data, as do surveys that collect information on all individuals in selected firms or establishments. However, in some countries we only have data on a sample of the individuals within each establishment, and in others we only have information on occupations for a subset of individuals who responded to a survey.

In most countries our baseline model (Model 1) provides similar estimates of gender differences when estimated on the full analytic sample (Table 1) and the sample of gender-integrated jobs (Supplementary Table 8). To the degree that our baseline model provides different estimates across these samples, this indicates that some of the sorting that drives the differences between the baseline and job-level estimates of the gender gap in Table 1 is sorting into single gender jobs. When we have information about all individuals working in a particular establishment the differences between Table 1 and Supplementary Table 8 indicate that there are important processes sorting women and men into single gender jobs. For example, the difference between the Danish gender gap in earnings from the Basic Adjustments models in Table 1 (-.178) and Supplementary Table 8 (-.148) indicates that approximately 3 percentage points of the Danish gender gap is due to sorting into single gender jobs with different pay. Importantly however, in countries where we only have information on a subset of employees within an establishment it is difficult to know whether differences between the estimates from the gender-integrated sample and the full sample reflect differences in the underlying population, or are specific to the sample in question.

**Supplementary Table 8.** Gender Differences in Earnings at Different Levels for Gender-Integrated Job Units

| | Year | Basic adj. | Fixed Effect for: | | | |
|---|---|---|---|---|---|---|
| | | | Est | Occ | Occ-Est | Proportion within job |
| Canada | 2015 | -.216 | -.161 | -.139 | -.121 | .56 |
| Czechia | 2019 | -.239 | -.187 | -.168 | -.123 | .51 |
| Denmark | 2015 | -.148 | -.102 | -.096 | -.072 | .49 |
| France | 2015 | -.135 | -.087 | -.079 | -.065 | .48 |
| Germany | 2015 | -.234 | -.139 | -.185 | -.130 | .56 |
| Hungary | 2017 | -.197 | -.138 | -.142 | -.095 | .48 |
| Israel | 2015 | -.290 | -.164 | -.131 | -.119 | .41 |
| Japan | 2013 | -.314 | -.277 | -.295 | -.257 | .82 |
| Netherlands | 2014 | -.126 | -.083 | -.078 | -.075 | .60 |
| Norway | 2018 | -.176 | -.106 | -.108 | -.086 | .49 |
| Slovenia | 2015 | -.222 | -.165 | -.181 | -.140 | .63 |
| South Korea | 2012 | -.322 | -.198 | -.292 | -.188 | .58 |
| Spain | 2017 | -.204 | -.150 | -.174 | -.121 | .59 |
| Sweden | 2018 | -.144 | -.100 | -.089 | -.076 | .53 |

In Hungary, the Netherlands, and Spain, our preliminary analyses found meaningful differences between the results from the baseline model (Model 1) when estimated on the subsample of gender-integrated jobs versus the full sample. Because the data in these countries do not include all employees within establishments, we are unable to conclude that these differences arise from sorting into single gender jobs in the population as they could be due to sampling within-establishments. To address this concern, we re-estimated models in these countries using post-stratification weights.

A simple example of the issue addressed by these weights is perhaps illustrative: in a job with one woman and one man, where each individual has a 50 percent chance of being included in the sample (as in Hungary), both the woman and the man have a 50 percent chance of being included in the full sample. But since both must be selected into the full sample for either to be in the gender-integrated sample, they have a 25 percent chance of being in the gender-integrated sample. Likewise, in the case of a job with three men and one woman, each of the three men has a 25 percent chance of being included in the gender-integrated sample (there is a 50 percent chance that they are in the full sample, and this is multiplied by the 50 percent chance that the one woman in the job is also in the full sample), while the one woman has a 43.75 percent chance of being included in the gender-integrated sample (she has a 50 percent chance of being in the full sample, and an 87.5 percent chance that at least one of the three men is in the full sample). The biases introduced by using such samples to examine within-establishment differences have the potential to be larger for samples selecting relatively few individuals within each establishment, as small establishments

(and establishments with relatively few people of a particular gender) will be particularly underrepresented in such samples. Our post-stratification weights seek to minimize the impact of these biases by weighting individuals based on the gender composition of workplaces and industries at the population level. Information on the variables used to construct the weights in Hungary, the Netherlands, and Spain is included in the country-specific data descriptions below (see Appendix C of the Supplementary Material).

***Age***. For our primary models, we use prime-age workers, defined as being between age 30 and 55, so as to mitigate differences related to country differences in maternity leave length. To ensure that our results are not sensitive to this restriction, we also estimated results across country specific age ranges in Canada (21-74), Czechia (16+), Denmark (16-68), France (all ages), Germany (16-64), Hungary (16-80), Israel (16-80), Japan (16-79), Netherlands (16-80), Norway (16-80), Slovenia (16-80), Spain (16-80), Sweden (18-67), and the United States (16+). Although comparing models with different age cutoffs does not fully account for issues that may arise around labor force participation when making comparisons across countries with different parental leave policies, and data constraints preclude a more formal analysis of how parental status might contribute to the differences that we observe, we find it reassuring that these analyses confirm that our results are robust to alternate age-cutoffs (see Supplementary Table 9).

**Supplementary Table 9.** Gender Differences in Earnings at Different Levels with Alternate Age Cutoffs for the Sample

| | Year | Basic Adj. | Fixed Effect for: | | | Proportion within job |
|---|---|---|---|---|---|---|
| | | | Est | Occ | Occ-Est | |
| Canada | 2015 | -.190 | -.137 | -.122 | -.103 | .54 |
| Czechia | 2019 | -.251 | -.198 | -.163 | -.105 | .42 |
| Denmark | 2015 | -.157 | -.119 | -.093 | -.064 | .41 |
| France | 2015 | -.107 | -.105 | -.080 | -.063 | .59 |
| Germany | 2015 | -.224 | -.160 | -.197 | -.125 | .56 |
| Hungary | 2017 | -.089 | -.108 | -.088 | -.082 | .92 |
| Israel | 2015 | -.312 | -.205 | -.189 | -.128 | .41 |
| Japan | 2013 | -.295 | -.268 | -.255 | -.211 | .72 |
| Netherlands | 2014 | -.171 | -.134 | -.102 | -.091 | .53 |
| Norway | 2018 | -.187 | -.116 | -.104 | -.074 | .40 |
| Slovenia | 2015 | -.184 | -.164 | -.155 | -.138 | .75 |
| South Korea | 2012 | -.338 | -.213 | -.269 | -.166 | .49 |
| Spain | 2017 | -.142 | -.153 | -.150 | -.105 | .74 |
| Sweden | 2018 | -.161 | -.110 | -.083 | -.068 | .42 |
| United States | 2015 | -.278 | -.181 | -.170 | -.119 | .43 |

Note: The alternate age cutoffs used varied by country as follows: Canada 21-74; Czechia 16+; Denmark 16-68; France all ages; Germany 16-64; Hungary 16-80; Israel 16-80; Japan 16-79; Netherlands 16-80; Norway 16-80; Slovenia 16-80; Spain 16-80; Sweden 18-67; US 16+.

***Persons vs. Employment Spells***. Many individuals change jobs in the middle of the year or hold multiple jobs concurrently in a year. In data from some countries, individuals are associated with their position as of a specific date, while in others every employment spell is logged separately. Analytically, one could focus on gender pay differences using employment spells in a given year as the unit of analysis or using individuals in a given year as the unit of analysis. Given our analytical focus, we prefer to use employment spells as our unit of analysis where possible. In Canada, Czechia, Denmark, France, Hungary, the Netherlands, and Slovenia, we use information from multiple employment spells in a year, while in Germany, Israel, Norway, Spain, Sweden, and the United States we use information from one employment spell per person per year as described in the country-specific descriptions in the Supplemental Material. In countries where we do not have employment spell information, we use individuals as our unit of analysis. Supplementary Table 10 reports supplemental analyses using persons within a year as the unit of analysis, confirming that results are largely similar regardless of whether persons or job spells are used as the unit of analysis.

**Supplementary Table 10.** Gender Differences in Earnings at Different Levels, Using Persons as Unit of Analysis

| | Year | Basic Adj. | Fixed Effect for: | | | Proportion within job |
|---|---|---|---|---|---|---|
| | | | Est | Occ | Occ-Est | |
| Czechia | 2019 | -.280 | -.225 | -.179 | -.123 | .44 |
| Denmark | 2015 | -.182 | -.137 | -.110 | -.075 | .41 |
| France | 2015 | -.121 | -.117 | -.095 | -.073 | .60 |
| Hungary | 2017 | -.106 | -.132 | -.100 | -.097 | .92 |
| Israel | 2015 | -.337 | -.197 | -.196 | -.119 | .35 |
| Netherlands | 2014 | -.295 | -.194 | -.197 | -.141 | .48 |
| Norway | 2018 | -.206 | -.128 | -.120 | -.086 | .42 |
| Slovenia | 2015 | -.190 | -.169 | -.157 | -.140 | .74 |
| Spain | 2017 | -.158 | -.176 | -.164 | -.121 | .77 |
| Sweden | 2018 | -.175 | -.118 | -.093 | -.076 | .43 |

Note: Each estimate represents the coefficient from a separate model estimating the difference between the logged earnings of women and men ages 30 to 55, with negative coefficients indicating that women earn less than men. Following standard conventions, we interpret these coefficients as the relative difference between the average female and male earnings, but more formally they indicate the difference in relative geometric means for unlogged earnings (which is the absolute difference in the arithmetic means of logged earnings). The "Basic Adjustment" column reports differences from a model that controls for age, age-squared, education, and full-time vs. part-time status, except in cases where a country is missing a particular measure. Subsequent models provide estimates of within-establishment, within-occupation, and within-job (occupation-establishment units) gender differences by introducing fixed effects for establishment, occupation, and occupation-establishment units. The final column reports the proportion of the gender difference from the first column (with only Basic Adjustments) that remains when we compare women and men who are working in the same occupations and establishments. P-values and confidence intervals are reported in Supplementary Table 30.

## Appendix C. Norwegian and Swedish Estimates in Prior Work

Previous studies using data from 1990 in Norway (*3*) and Sweden (*2*) found that gender differences in hourly wages arose overwhelmingly due to sorting, and that there were relatively small within-job gender differences. In Norway, prior work found a 21 percent overall gender gap in wages and a four percent within-job gender difference, so that the within-job gender gap accounted for 18 percent of the overall gender gap, and 82 percent of the overall gender gap was accounted for by sorting into jobs. Similarly, in Sweden prior work found an 18 percent overall gap in wages and a within-job gap of 2.4 percent, so that the within-job gender gap accounted for 13 percent of the overall gender gap and sorting accounted for 87 percent of the overall gender gap. Although our primary specification examined earnings differences, in Table 3 we report gender differences in hourly wage for a sub-set of countries. As noted above, we typically find smaller gender differences in wages (Supplementary Table 2) than in earnings (Table 1). Importantly, as both the overall gender wage gap and the within-job wage gap tend to be smaller in Supplementary Table 2 than in Table 1, on balance we find that sorting is similarly important for understanding gender differences in wages and earnings across our 15 countries.

Comparing our estimates of wage differences in Norway and Sweden to previous findings, we find that sorting accounts for less of the overall wage gap (and within-job differences account for more) than in prior work. Supplementary Table 2 shows that in Norway within-job wage differences account for 34 percent of the overall wage gap (versus 18 percent in prior work) and in Sweden within-job wage differences account for 28 percent of the overall wage gap (versus 13 percent in prior work). These differences arise because the more recent estimates of the overall gender wage gaps reported in Supplementary Table 2 are smaller (-.137 in Norway and -.125 in Sweden; compared to -.214 in Norway and -.182 in Sweden in prior work), and also because the within-job gender gaps in Supplementary Table 2 are slightly larger (-.046 in Norway and -.035 in Sweden; compared to -.038 and -.024, respectively, in prior work).

There are nine primary ways in which our analytical approach differs from that of prior work in Norway and Sweden. Specifically, the current study differs from prior work in that prior work: 1) used data ending in 1990 and the current study uses more recent data; 2) used selected industries in the private sector and the current study covers the economy more broadly; 3) did not control for age or education; 4) did not distinguish between full-time and part-time workers; 5) examined workers of all ages and the current study focuses on prime age workers; 6) used a more detailed occupation code than the 4-digit ISCO codes used in our primary specification; 7) examined differences in hourly wage, while our primary specification examines annual earnings; 8) measures the gender gap using the mean of an unlogged ratio, while the current analyses use a logged dependent variable; and 9) treated each job cell as one observation in analyses of within-job inequality (regardless of the number of employees in the cell), while the current analyses weight job cells by the number of employees.

To better understand how each decision affects the relative importance of sorting, we replicate prior work as closely as is possible with our data, and then iteratively change each of these analytical decisions, observing how the relative importance of sorting changes in these different specifications. Across Norway and Sweden the primary factors that account for the differences in sorting that we observe are: 1) controlling for full versus part-time status in the models, and 2) using a logged dependent variable (as opposed to an unlogged ratio). Accounting for full versus part-time status matters more through changing the estimate of the population-level gender gap, and using a logged dependent variable matters more through how it changes within-job differences. As we cannot directly replicate prior analyses our conclusions here are necessarily speculative, but our analyses suggest that the differences in the relative importance of sorting between our study and prior work is the result of these two analytical choices.

## Appendix D. Country-specific Data Descriptions and Trends

***Canada***. Our analyses use the linkage between Canadian census long-form files and the Longitudinal Worker File (LWF). Information on individuals' educational level and the occupation of their main job comes from the 2006 census (a mandatory census sent to one in five households, with a 94 percent response rate), the 2011 National Household Survey (a voluntary survey sent to one in three households, with a weighted response rate of 77 percent), and the 2016 census (a mandatory census sent to one in four households, with a response rate of 98 percent). The occupation codes are based on Canada's National Occupational Classification at the four-digit level, with about 520 categories. These census/NHS files are linked to the LWF in the corresponding income year (e.g., the income year for the 2016 census is 2015, so the 2016 census is linked with the 2015 LWF) using the linkage keys developed by Statistics Canada.

The LWF is an administrative file that contains basic demographic characteristics (age, gender and geographic region) and person-job information for all workers who were issued a T4 form (Statement of Remuneration Paid) by their employer in a given year. All employers in Canada are required to complete the T4 forms for their employees on an annual basis. For this study, the LWF provides the annual earnings of a job and the identifier of the employer (firm). The LWF does not have information on weeks and hours worked, so we do not report results for wages.

Our data allow us to examine how gender differences have changed across the years 2005, 2010, and 2015. Supplementary Table 11 and Supplementary Figure 1 present information on the Canadian trends in the gender gap overall, as well as within establishments, occupations, and jobs. Supplementary Table 11 also reports results from models estimated using a sample of gender-integrated jobs that is consistent across models within each year. The data files used for this project can be accessed at Statistics Canada upon receipt of a security check and authorization from Statistics Canada.

**Supplementary Table 11.** Canadian Trends in Gender Differences in Earnings

| | All Workplaces | | | | Gender-integrated Jobs Only | | | |
|---|---|---|---|---|---|---|---|---|
| | Basic Adj. | Fixed Effect for: | | | Basic Adj. | Fixed Effect for: | | |
| Year | | Est | Occ | Occ-Est | | Est | Occ | Occ-Est |
| 2005 | -.240 | -.208 | -.168 | -.153 | -.248 | -.194 | -.168 | -.153 |
| 2010 | -.215 | -.184 | -.150 | -.136 | -.217 | -.175 | -.148 | -.136 |
| 2015 | -.221 | -.172 | -.137 | -.121 | -.216 | -.161 | -.139 | -.121 |

**Supplementary Figure 1.** Trends in Canadian Gender Differences in Earnings

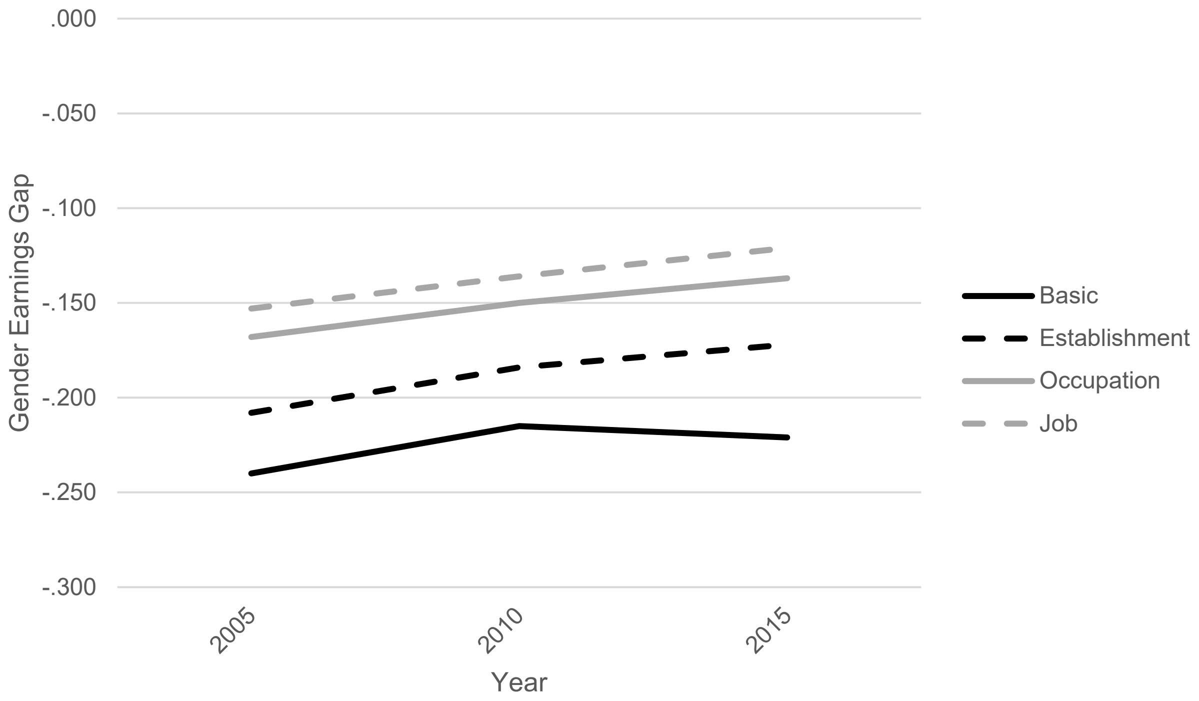


***Czechia***. Our analyses use data from the Average Earnings Information System (*Informační systém o průměrném výdělku - ISPV*) for the private sector and the Information System on Wages and Service Income (*Informační systémy o platu a služebním příjmu - ISPSP*) for the public sector. The ISPV is a survey conducted by Trexima, spol. s r.o. for the Ministry of Labor and Social Affairs. The content of the survey is aligned with the EU Structure of Earnings Survey (Commission Regulation (EC) No 1916/2000). Trexima, spol. s r.o. publishes results quarterly (http://www.ispv.cz) and aggregate results are also published by the Czech Statistical Office (http://www.czso.cz). For the private sector the survey takes place every quarter and is compulsory for selected firms with more than 10 employees. Each quarter data on wages, hours worked, and the number of employees are collected from employers. Biannual data about wages, hourly wages, hours worked, age, gender, education, tenure with the employer, type of contract, occupation, citizenship and place of work are collected for all employees in these firms. Analogous public sector

data are collected biannually by the ISPSP, which is administered by the Ministry of Finance and covers all public sector employees. Combined, the ISPV and ISPSP data are collected from about 51 percent of employees in Czechia and representatively cover 82 percent of Czech employees.

Occupation is based on the ISCO classification (5-digit) completed by employers for each employee. Education is measured using 15 categories ranging from no education (1) to a doctoral degree (15), which we recode into 4 categories. There is an Identification Number (a firm-level identifier) for each employer. As a proxy for establishment we use the information on place of work for each employee combined with the firm Identification Number. Place of work is based on Czechia's 78 Local Administrative Units (LAU1) districts.

Our data allow us to examine how gender differences have changed from 2002 to 2019. Supplementary Table 12 and Supplementary Figure 2 present information on the trends in the Czech gender gap overall, as well as within establishments, occupations, and jobs. Table S3 also reports results from models estimated using a sample of gender-integrated jobs that is consistent across models within each year. The data used for this project can be accessed at the Trexima, spol. s r.o. upon receipt of proper authorizations from the Ministry of Labor and Social Affairs of the Czech Republic.

**Supplementary Table 12.** Czech Trends in Gender Differences in Earnings

| | All Workplaces | | | | Gender-integrated Jobs Only | | | |
|---|---|---|---|---|---|---|---|---|
| | Basic Adj. | Fixed Effect for: | | | Basic Adj. | Fixed Effect for: | | |
| Year | | Est | Occ | Occ-Est | | Est | Occ | Occ-Est |
| 2002 | -.235 | -.198 | -.186 | -.123 | -.203 | -.159 | -.164 | -.123 |
| 2003 | -.243 | -.203 | -.192 | -.127 | -.209 | -.163 | -.166 | -.127 |
| 2004 | -.254 | -.189 | -.193 | -.123 | -.232 | -.167 | -.165 | -.123 |
| 2005 | -.263 | -.198 | -.198 | -.132 | -.236 | -.174 | -.171 | -.132 |
| 2006 | -.268 | -.209 | -.209 | -.134 | -.238 | -.180 | -.180 | -.134 |
| 2007 | -.267 | -.209 | -.203 | -.129 | -.226 | -.178 | -.176 | -.129 |
| 2008 | -.294 | -.207 | -.195 | -.123 | -.233 | -.171 | -.167 | -.123 |
| 2009 | -.283 | -.214 | -.202 | -.133 | -.238 | -.182 | -.175 | -.133 |
| 2010 | -.279 | -.210 | -.193 | -.123 | -.232 | -.170 | -.161 | -.123 |
| 2011 | -.276 | -.209 | -.191 | -.126 | -.230 | -.178 | -.170 | -.126 |
| 2012 | -.280 | -.213 | -.203 | -.129 | -.240 | -.179 | -.180 | -.129 |
| 2013 | -.284 | -.221 | -.203 | -.137 | -.234 | -.185 | -.188 | -.137 |
| 2014 | -.295 | -.221 | -.196 | -.135 | -.242 | -.190 | -.180 | -.135 |
| 2015 | -.313 | -.227 | -.200 | -.133 | -.249 | -.191 | -.178 | -.133 |
| 2016 | -.309 | -.233 | -.203 | -.132 | -.253 | -.194 | -.181 | -.132 |
| 2017 | -.300 | -.228 | -.190 | -.122 | -.247 | -.184 | -.170 | -.122 |
| 2018 | -.289 | -.225 | -.182 | -.120 | -.243 | -.184 | -.164 | -.120 |
| 2019 | -.280 | -.225 | -.179 | -.123 | -.239 | -.187 | -.168 | -.123 |

**Supplementary Figure 2.** Trends in Czech Gender Differences in Earnings

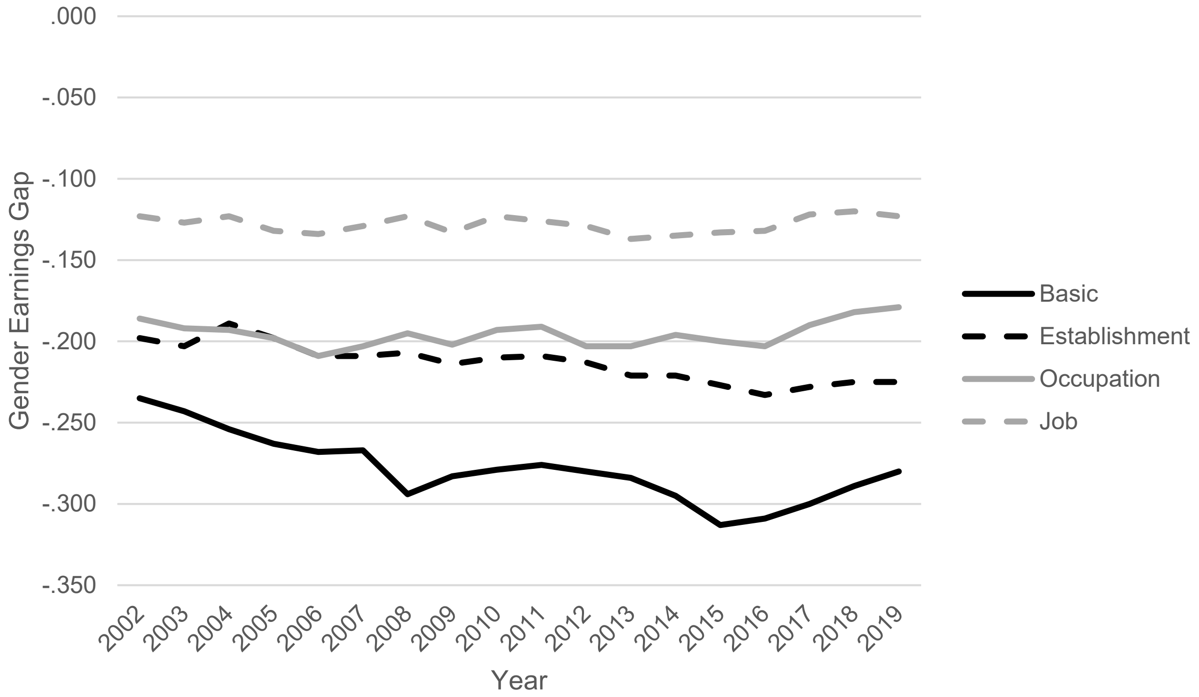


***Denmark***. Our analyses use data from Statistics Denmark's registry-based Labor Force Statistics (RAS, *Den Registerbaserede Arbejdsstyrkestatistik*) and the Integrated Database for Labor Market Research (IDA, *Den Integrerede Database for Arbejdsmarkedsforskning*) for information on hourly earnings, part- versus full-time status, occupations, and establishments. These data are collected once per year in November and provide information on all employment spells in both the public and private sector. Information on employment spells comes from employer-reported tax records, which distinguish primary, secondary, and tertiary jobs. As tertiary jobs are associated with poorer data quality, we exclude them from our analyses, and use data on primary and secondary jobs with earnings that are greater than half of the tenth percentile of the earnings distribution.

Our measure of hourly wages is derived from the LONN variable FORTJ_STAND which is a standardized hourly wage measure excluding wages during holidays and illness as well as overtime (for details on FORTJ_STAND, see https://www.dst.dk/da/Statistik/dokumentation/Times/loen/fortj-stand. This variable is available for all employees in the public sector but in the private sector reporting is only mandatory for establishments with 10 or more employees and so a weighting scheme has been developed to adjust for the sample bias (for details on weighting scheme, see pages 47-49 in http://www.dst.dk/pukora/epub/upload/17075/loen.pdf).

Occupation is based on Statistics Denmark's four-digit version of ISCO-88 and ISCO-08 called DISCO. Information about gender and age is based on records from the Central Population Register (BEF, *Befolkningsregisteret*). Information about education refers to each individual's highest level of educational qualifications in each year based on annual records from the National Students Register (KOTRE, *Det Komprimerede Elevregister*). Information on educational level is measured using four categories (elementary, high school, vocational, and college).

Our data allow us to examine how gender earnings differences have changed from 1994-2015. Supplementary Table 13 and Supplementary Figure 3 present information on the trends in the Danish gender gap overall, as well as within establishments, occupations, and jobs. Table S4 also reports results from models estimated using a sample of gender-integrated jobs that is consistent across models within each year. Beginning in 2008 the registry data include the so-called e-income register, which includes a wider range of jobs and identifies earnings more precisely, leading to a structural break in the trend. Data similar to those used for this project can be accessed at Statistics Denmark upon receipt of proper authorizations and after paying the relevant fees.

**Supplementary Table 13.** Danish Trends in Gender Differences in Earnings

| | All Workplaces | | | | Gender-integrated Jobs Only | | | |
|---|---|---|---|---|---|---|---|---|
| | Basic Adj. | Fixed Effect for: | | | Basic Adj. | Fixed Effect for: | | |
| Year | | Est | Occ | Occ-Est | | Est | Occ | Occ-Est |
| 1994 | -.234 | -.173 | -.139 | -.092 | -.205 | -.147 | -.119 | -.092 |
| 1995 | -.237 | -.168 | -.140 | -.087 | -.182 | -.126 | -.111 | -.087 |
| 1996 | -.239 | -.171 | -.142 | -.085 | -.183 | -.128 | -.112 | -.085 |
| 1997 | -.241 | -.175 | -.148 | -.088 | -.194 | -.130 | -.117 | -.088 |
| 1998 | -.247 | -.181 | -.153 | -.095 | -.196 | -.137 | -.127 | -.095 |
| 1999 | -.248 | -.181 | -.156 | -.097 | -.206 | -.138 | -.127 | -.097 |
| 2000 | -.251 | -.186 | -.167 | -.110 | -.208 | -.148 | -.145 | -.110 |
| 2001 | -.249 | -.184 | -.166 | -.109 | -.206 | -.146 | -.143 | -.109 |
| 2002 | -.246 | -.182 | -.163 | -.110 | -.206 | -.146 | -.144 | -.110 |
| 2003 | -.238 | -.177 | -.155 | -.111 | -.201 | -.144 | -.139 | -.111 |
| 2004 | -.234 | -.180 | -.156 | -.110 | -.197 | -.144 | -.139 | -.110 |
| 2005 | -.231 | -.169 | -.153 | -.105 | -.199 | -.135 | -.134 | -.105 |
| 2006 | -.230 | -.170 | -.157 | -.111 | -.199 | -.140 | -.138 | -.111 |
| 2007 | -.232 | -.173 | -.162 | -.111 | -.202 | -.142 | -.141 | -.111 |
| 2008 | -.201 | -.153 | -.132 | -.087 | -.168 | -.119 | -.114 | -.087 |
| 2009 | -.178 | -.139 | -.122 | -.081 | -.154 | -.110 | -.108 | -.081 |
| 2010 | -.182 | -.143 | -.118 | -.079 | -.159 | -.112 | -.105 | -.079 |
| 2011 | -.182 | -.139 | -.114 | -.076 | -.158 | -.108 | -.102 | -.076 |
| 2012 | -.177 | -.137 | -.111 | -.075 | -.154 | -.106 | -.100 | -.075 |
| 2013 | -.176 | -.134 | -.109 | -.074 | -.151 | -.105 | -.098 | -.074 |
| 2014 | -.177 | -.133 | -.108 | -.074 | -.150 | -.104 | -.097 | -.074 |
| 2015 | -.178 | -.132 | -.107 | -.072 | -.148 | -.102 | -.096 | -.072 |

**Supplementary Figure 3.** Trends in Danish Gender Differences in Earnings

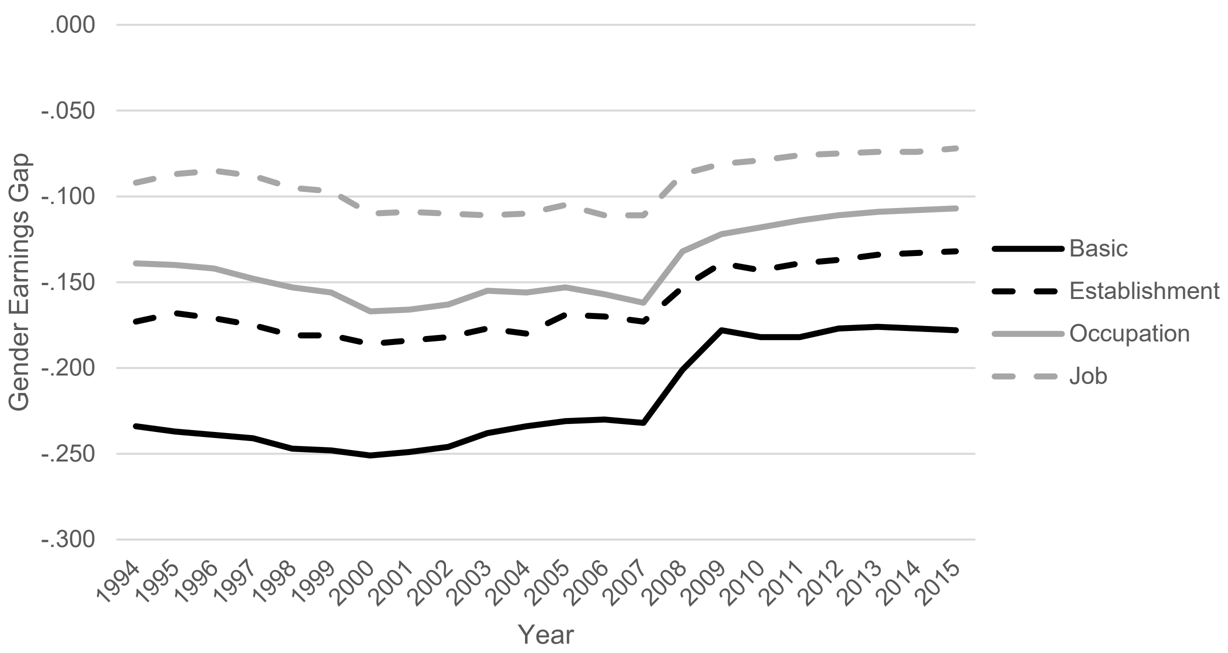


***France***. Our analyses use data from the DADS social security register (*Déclaration Annuelle de Données Sociales*). The data consist of population-level observations of private sector workers, plus all hospital and local civil service workers; state civil servants are included beginning 2009. We compute hourly wage using employees' annual gross wages and hours information, taking into account compulsory overtime bonuses. For instance, overtime hours between 35 hours and 43 hours per week get paid at least 25 percent more, and hours above 43 hours per week get paid 50 percent more.

Person-job matches that report earnings less than half of the yearly minimum wage are excluded. This leads to exclude approximately a third of job spells and 20 percent of individuals, mostly very short-term job spells. Our measure of occupation is the four-digit *Nomenclature des Professions et Categories Socio-Professionnelles* (CSP), which contains approximately 400 unique occupational codes. Codes for each worker are reported by French firms in the DADS and occasionally contain errors, especially in early years. When an invalid four-digit CSP code is used, we draw upon the nested design of the codes to use the most detailed valid code available. For example, the four-digit CSP code "376a" for "Financial market professionals" is nested within one-, two-, and three-digit codes corresponding to "Managers and professionals" ("3"), "Administrative and retail managers" ("37"), and "Bank, insurance, real estate professionals" ("376"). When firms use incorrect four-digit codes such as "376h," "379a," or "396a," we use the most detailed valid code possible, which in these cases would respectively be "376," "37," and "3" as their occupational codes. In 2015, 94 percent of the working population had a valid four-digit code and 6 percent had

a valid one-digit code. In contrast, in 2000, 34 percent had a valid four-digit code, 10 percent a valid three-digit code, 46 percent a valid two-digit code, 8 percent a valid one-digit code, and 2 percent had no valid digit code (and were thus dropped from the sample).

Our data do not include information on employees' education. We conduct supplementary analyses in which we use the linked census EDP-DADS panel data which contains information about education for one percent of the population. Results from these models (see Supplementary Table 6) suggest that the gender earnings gap net of education is several percentage points larger than the gender earnings gap we observe when we do not include this control. Note that because we are working with a relatively small sample, we use two-digit occupations for analyses using the EDP-DADS data.

Our data allow us to examine how gender differences have changed from 1993 to 2015 using the DADS social security data. Supplementary Table 14 and Supplementary Figure 4 present information on the trends in the French gender gap overall, as well as within establishments, occupations, and jobs. Supplementary Table 14 also reports results from models estimated using a consistent sample of gender-integrated jobs in each year. As state civil servants are only included beginning in 2009, to ensure that their inclusion does not change our trends we estimate results from 2009 both with and without state civil servants. It is also important to note that there is a break in the data in 2001, and that occupation data are missing for a substantial portion of the population (up to 30 percent) before 2003; as such, results from before 2003 should be interpreted with caution. Supplementary Table 15 reports results on trends from 1976 to 2015 using the longer time-series available in the one percent EDP-DADS panel data. As the EDP-DADS data include a measure of education (unlike the DADS social security data used in the main analyses), we present trend results from models both with and without education in Supplementary Table 15. Supplementary Figure 5 presents the trends in the gender gap from the EDP-DADS models that do not include education. Access to the DADS data can be obtained from the CASD dedicated to researchers authorized by the French *Comité du Secret Statistique*.

**Supplementary Table 14.** French Trends in Gender Differences in Earnings from Social Security Data

| | All Workplaces | | | | Gender-integrated Jobs Only | | | |
|---|---|---|---|---|---|---|---|---|
| | Basic Adj. | Fixed Effect for: | | | Basic Adj. | Fixed Effect for: | | |
| Year | | Est | Occ | Occ-Est | | Est | Occ | Occ-Est |
| 1993 | -.183 | -.222 | -.140 | -.105 | -.210 | -.166 | -.125 | -.105 |
| 1994 | -.166 | -.212 | -.126 | -.097 | -.200 | -.158 | -.116 | -.097 |
| 1995 | -.151 | -.197 | -.109 | -.090 | -.176 | -.148 | -.105 | -.090 |
| 1996 | -.136 | -.191 | -.104 | -.091 | -.174 | -.146 | -.099 | -.091 |
| 1997 | -.126 | -.186 | -.101 | -.090 | -.165 | -.144 | -.097 | -.090 |
| 1998 | -.122 | -.181 | -.098 | -.087 | -.163 | -.140 | -.096 | -.087 |
| 1999 | -.116 | -.176 | -.097 | -.085 | -.153 | -.134 | -.093 | -.085 |

| | | | | | | | | |
|---|---|---|---|---|---|---|---|---|
| 2000 | -.123 | -.173 | -.100 | -.083 | -.154 | -.131 | -.094 | -.083 |
| 2001 | -.130 | -.171 | -.102 | -.083 | -.160 | -.130 | -.096 | -.083 |
| 2002 | -.140 | -.167 | -.097 | -.080 | -.168 | -.126 | -.089 | -.080 |
| 2003 | -.139 | -.164 | -.095 | -.081 | -.168 | -.125 | -.090 | -.081 |
| 2004 | -.136 | -.159 | -.092 | -.078 | -.167 | -.123 | -.087 | -.078 |
| 2005 | -.134 | -.154 | -.092 | -.076 | -.163 | -.118 | -.090 | -.076 |
| 2006 | -.134 | -.149 | -.093 | -.078 | -.169 | -.116 | -.095 | -.078 |
| 2007 | -.129 | -.143 | -.089 | -.078 | -.161 | -.112 | -.091 | -.078 |
| 2008 | -.133 | -.142 | -.089 | -.079 | -.162 | -.112 | -.089 | -.079 |
| 2009(a) | -.132 | -.135 | -.101 | -.079 | -.164 | -.108 | -.092 | -.079 |
| 2009(b) | -.124 | -.130 | -.103 | -.074 | -.154 | -.104 | -.095 | -.074 |
| 2010 | -.127 | -.126 | -.088 | -.071 | -.157 | -.101 | -.084 | -.071 |
| 2011 | -.125 | -.125 | -.092 | -.074 | -.152 | -.100 | -.090 | -.074 |
| 2012 | -.120 | -.119 | -.089 | -.069 | -.147 | -.094 | -.085 | -.069 |
| 2013 | -.117 | -.115 | -.087 | -.068 | -.144 | -.092 | -.084 | -.068 |
| 2014 | -.114 | -.112 | -.086 | -.067 | -.139 | -.089 | -.081 | -.067 |
| 2015 | -.111 | -.108 | -.084 | -.065 | -.135 | -.087 | -.079 | -.065 |

Note: We report two sets of estimates for 2009, when civil servants were first included in the data: 2009(a) matches prior year results and does not include civil servants; 2009(b) matches later year results and includes civil servants.

**Supplementary Table 15.** French Trends in Gender Differences in Earnings from 1% Panel Data, With and Without Education

| | Without Education (Main French Specification) | | | | With Education | | | |
|---|---|---|---|---|---|---|---|---|
| | Basic Adj. | Fixed Effect for: | | | Basic Adj. | Fixed Effect for: | | |
| Year | | Est | Occ | Occ-Est | | Est | Occ | Occ-Est |
| 1976 | -.281 | -.326 | -.193 | -.159 | -.273 | -.272 | -.192 | -.157 |
| 1977 | -.280 | -.322 | -.190 | -.147 | -.273 | -.268 | -.191 | -.150 |
| 1978 | -.256 | -.295 | -.181 | -.156 | -.253 | -.241 | -.181 | -.155 |
| 1979 | -.239 | -.295 | -.157 | -.128 | -.232 | -.240 | -.156 | -.127 |
| 1980 | -.239 | -.277 | -.171 | -.125 | -.238 | -.233 | -.171 | -.123 |
| 1982 | -.213 | -.259 | -.150 | -.116 | -.211 | -.213 | -.149 | -.114 |
| 1984 | -.201 | -.249 | -.171 | -.128 | -.202 | -.199 | -.165 | -.124 |
| 1985 | -.191 | -.247 | -.166 | -.130 | -.191 | -.199 | -.159 | -.121 |
| 1986 | -.185 | -.235 | -.169 | -.113 | -.190 | -.194 | -.163 | -.115 |
| 1987 | -.204 | -.234 | -.168 | -.095 | -.205 | -.194 | -.165 | -.095 |
| 1988 | -.187 | -.224 | -.149 | -.104 | -.191 | -.190 | -.146 | -.103 |
| 1989 | -.197 | -.229 | -.158 | -.107 | -.200 | -.195 | -.155 | -.106 |
| 1991 | -.201 | -.219 | -.154 | -.106 | -.208 | -.187 | -.154 | -.105 |
| 1992 | -.197 | -.203 | -.140 | -.090 | -.206 | -.176 | -.141 | -.089 |
| 1993 | -.198 | -.203 | -.152 | -.094 | -.206 | -.184 | -.154 | -.095 |
| 1994 | -.188 | -.172 | -.145 | -.080 | -.199 | -.157 | -.147 | -.082 |
| 1995 | -.165 | -.178 | -.131 | -.091 | -.178 | -.163 | -.132 | -.094 |
| 1996 | -.154 | -.166 | -.122 | -.087 | -.173 | -.156 | -.125 | -.091 |

| | | | | | | | | |
|---|---|---|---|---|---|---|---|---|
| 1997 | -.147 | -.162 | -.113 | -.078 | -.165 | -.150 | -.116 | -.082 |
| 1998 | -.132 | -.152 | -.106 | -.081 | -.156 | -.143 | -.109 | -.084 |
| 1999 | -.136 | -.172 | -.108 | -.078 | -.160 | -.157 | -.111 | -.079 |
| 2000 | -.141 | -.176 | -.114 | -.084 | -.168 | -.163 | -.117 | -.086 |
| 2001 | -.156 | -.164 | -.114 | -.067 | -.179 | -.150 | -.118 | -.068 |
| 2002 | -.157 | -.162 | -.106 | -.085 | -.180 | -.151 | -.109 | -.086 |
| 2003 | -.155 | -.162 | -.104 | -.093 | -.179 | -.153 | -.107 | -.094 |
| 2004 | -.152 | -.153 | -.102 | -.083 | -.179 | -.145 | -.106 | -.084 |
| 2005 | -.149 | -.151 | -.096 | -.084 | -.177 | -.145 | -.101 | -.085 |
| 2006 | -.147 | -.145 | -.094 | -.086 | -.174 | -.140 | -.098 | -.087 |
| 2007 | -.142 | -.144 | -.089 | -.087 | -.169 | -.141 | -.094 | -.088 |
| 2008 | -.143 | -.141 | -.090 | -.086 | -.171 | -.140 | -.094 | -.088 |
| 2009 | -.131 | -.139 | -.117 | -.096 | -.180 | -.139 | -.121 | -.097 |
| 2010 | -.131 | -.133 | -.095 | -.089 | -.177 | -.134 | -.100 | -.091 |
| 2011 | -.147 | -.154 | -.119 | -.109 | -.183 | -.155 | -.124 | -.109 |
| 2012 | -.127 | -.124 | -.100 | -.090 | -.176 | -.128 | -.105 | -.091 |
| 2013 | -.126 | -.122 | -.098 | -.089 | -.175 | -.126 | -.102 | -.090 |
| 2014 | -.122 | -.118 | -.094 | -.086 | -.172 | -.123 | -.099 | -.086 |
| 2015 | -.119 | -.113 | -.091 | -.082 | -.169 | -.118 | -.096 | -.083 |

**Supplementary Figure 4.** Trends in French Gender Differences in Earnings

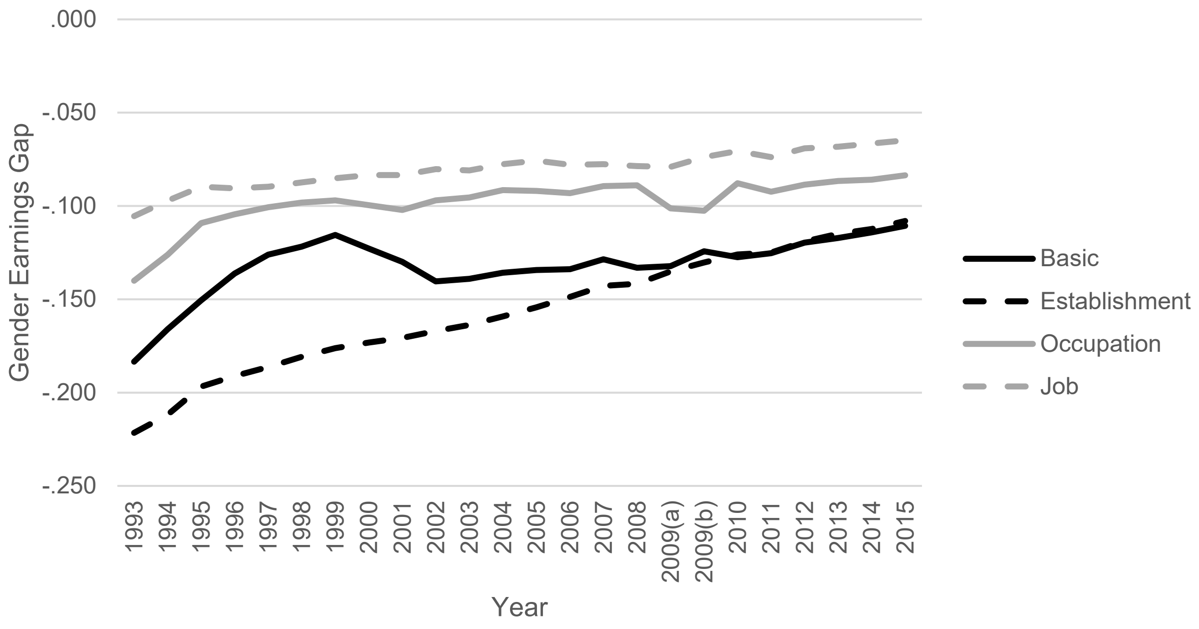

**Supplementary Figure 5.** Trends in French Gender Differences in Earnings(from EDP-DADS 1% Panel w/out Education in the Model)

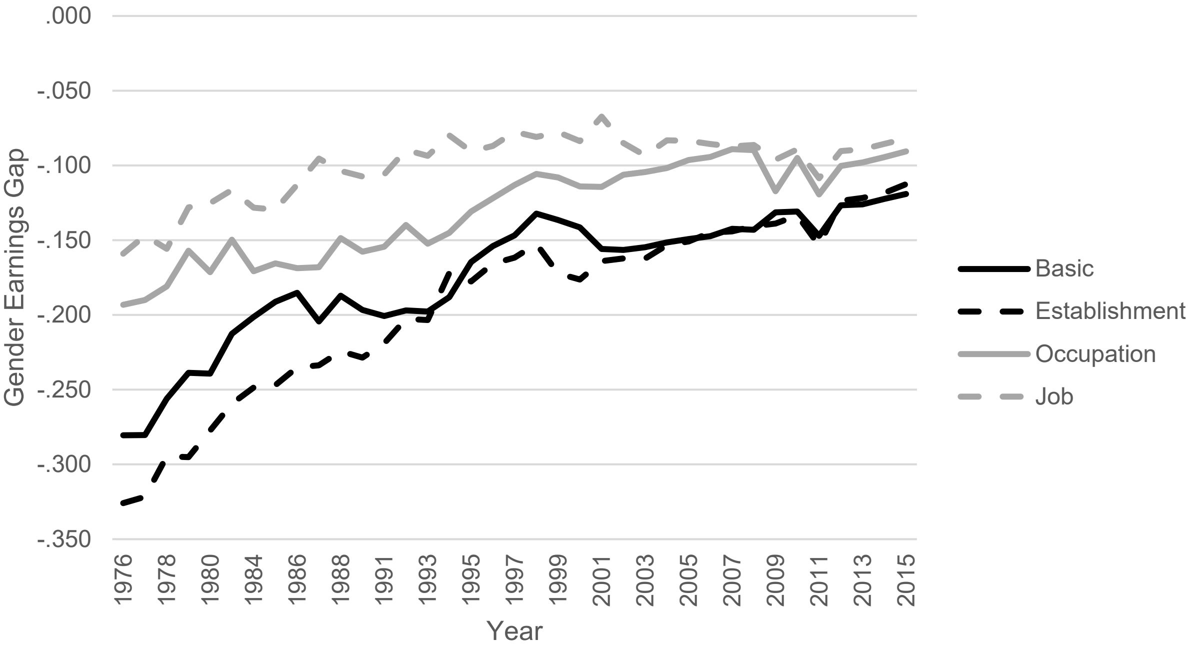


***Germany***. Our analyses use a customized sample for the project "Dynamics of organizational earnings inequality: Investigation within the Comparative Organizational Inequality International Network (COIN)" of the Integrated Employment Biographies Sample (IEBS) combining records of the employment history (BeH) and benefit recipient history (LeH) of the Federal Employment Agency. The customized sample of the IEBS was drawn in 2017 and roughly covers five percent of the German employed population and 20,000 establishments. The data spans from 1990 to 2015, and East Germany is included from 1992 onwards. As the East German data reaches the quality of the West German data in 1993, we focus our analyses on data from 1993 to 2015. Although many of the other countries analyzed for this project have substantial regional variation, the German case—with the relatively recent unification of East and West Germany—is unique. Given the different norms and ideologies (e.g., *6, 7*) we present supplemental analyses with results separately for East and West Germany.

The basis for the data is the integrated notification procedure for health, pension and unemployment insurance, which came into effect in 1973 and was extended to cover Eastern Germany in 1991. Under this procedure employers are required to submit notifications to the responsible social security agencies concerning all their employees covered by social security at least once a year. Thus, our data covers the approximately 80 percent of the workforce that is liable for social security contributions, but excludes elite civil servants (*Beamte*) and the self-employed.

These data represent a sample of firms and their employees. We first randomly sample 20,000 establishments among all establishments that existed in Germany between 1993 and 2013 (without regard to the duration of their existence or their region). The establishments were drawn proportionally to their size (c.f. *8*) across the whole panel period, and smaller firms are selected with a decreasing probability. For privacy reasons, we limit the maximum of the sampling probability to 0.3, as otherwise, due to the skewness of the workplace size distribution, nearly all large workplaces would be drawn into the sample. This sampling strategy reproduces both workplace and individual population parameters.

We then select employees from the 20,000 establishments. For very large establishments, the number of employees was limited to 1,000 randomly selected employees. For all others, all employees are selected. Once an individual was selected into the sample, all available information on the individual between 1990 and 2015 was provided even if the employee was working only for a limited period in the previously selected establishments. The customized sample of the Integrated Employment Biographies Sample (IEBS) are episode data (i.e., each observation has a start date and an end date). The data are transformed from spell into panel data to estimate the models, and for each employee we keep information on one job per year. Given our interest in comparing women and men in the same establishments and occupations, we focus on observations in the 20,000 selected establishments. Regardless of the amount of time spent in other establishments, if an employee spent at least one day in a given year working in one of the 20,000 selected establishments, the information from this establishment is selected into our analytic sample over information on employment in other establishments. If an employee is not employed in one of our 20,000 sampled establishments (e.g., took a job in a different establishment), and has more than one employment spell, we select the employment spell with the longer duration. Finally, if employees held multiple jobs in one or more of the selected establishments for the same duration, we select the one with the higher income.

The data provide information on private and public-sector establishments from all industries. Daily earnings, averaged across the employment period (e.g., the average daily earnings for one year) are reported. Bonuses are included in these earnings, and it is not possible to differentiate between the bonus paid and other sources of earnings (e.g., contractual wage on contractual hours), or to know whether a bonus was paid or not. Incomes not obligated to pay social security because they are below the threshold for small-scale employment (e.g., newspaper delivery), which is currently €450 per month, are excluded from the sample. They were automatically excluded from the sample until 1999 and are removed by excluding earnings up to € 2 above the threshold after. The earnings are also top-coded at the social contribution limit, which differs by year and in East and West Germany. We consider all daily earnings that are larger than the contribution limit minus 2€ as censored in order to account for rounding errors. To impute the top-coded earnings we follow Card, Heining and Kline (*9*) and use information on individual and workplace wages prior to the censored period. However, rather than focusing on the mean individual and workplace wage prior

to the censored observation, as was done by Card and coauthors, we utilize information on lagged earnings. We reason that the censored earnings are more strongly influenced by the most recent period than by mean earnings over longer periods. Using lagged information, the earnings distribution is smoother than the distribution created by replicating Card's imputation model (*10*).

The data contain no information on the hours worked, but differentiate between full- and part-time work, with part-time employees defined as those working 18 hours or less. Occupation is based on a German version of ISCO-08 (*Klassifizierung der Berufe*), which is fully transferrable to ISCO-08 four-digit occupations. Vocational training plays an important role in the German labor market, and typically involves not only academic instruction in schools but also apprenticeship-based training in workplaces (on-the-job training). We thus use two separate variables to measure education. The first differentiates between persons with: 1) no school certificate; 2) lower secondary school certificate; 3) intermediate school certificate; and 4) upper secondary school certificate (which is comparable with the A-level and is necessary to enter universities). The second education variable accounts for vocational training and university degrees, qualifications which are gained after secondary education, using the categories: 1) no vocational training; 2) vocational training; 3) university of applied science degree; and 4) university degree.

Our data allow us to examine how gender differences have changed from 1993 to 2015. Supplementary Table 16 and Figure S6 present information on the trends in the German gender gap overall, as well as within establishments, occupations, and jobs. Given the salience of regional differences in Germany, we also present this information separately for East and West Germany. Supplementary Table 17 and Supplementary Figure 7 present the East German results, and Supplementary Table 18 and Supplementary Figure 8 present results from West Germany. Supplementary Tables 16, 17, and 18 also report results from models estimated using a consistent sample of gender-integrated jobs in each year. Our results are consistent with the idea that gender inequality operates differently in East and West Germany. Specifically, while establishment and occupational sorting in West Germany follow the pattern observed elsewhere and typically advantages men (i.e., men sort into establishments, occupations, and jobs that tend to have higher pay, so that within-establishment, within-occupation, and within-job gender gaps are smaller than the gender gap in the labor market more broadly), in East Germany these sorting processes tend to advantage women, so that the gender gap becomes larger when we compare women with men who work in the same establishments, occupations, and jobs. Data similar to those used for this project can be accessed at the Institute for Employment Research upon receipt of proper authorizations.

**Supplementary Table 16.** German Trends in Gender Differences in Earnings

| | All Workplaces | | | | Gender-integrated Jobs Only | | | |
|---|---|---|---|---|---|---|---|---|
| | Basic Adj. | Fixed Effect for: | | | Basic Adj. | Fixed Effect for: | | |
| Year | | Est | Occ | Occ-Est | | Est | Occ | Occ-Est |
| 1993 | -.307 | -.214 | -.365 | -.199 | -.258 | -.193 | -.285 | -.199 |

| | | | | | | | | |
|---|---|---|---|---|---|---|---|---|
| 1994 | -.294 | -.211 | -.350 | -.195 | -.255 | -.190 | -.275 | -.195 |
| 1995 | -.288 | -.208 | -.344 | -.191 | -.251 | -.188 | -.269 | -.191 |
| 1996 | -.277 | -.203 | -.328 | -.192 | -.246 | -.186 | -.262 | -.192 |
| 1997 | -.271 | -.200 | -.317 | -.187 | -.244 | -.183 | -.256 | -.187 |
| 1998 | -.274 | -.212 | -.319 | -.199 | -.255 | -.200 | -.266 | -.199 |
| 1999 | -.270 | -.209 | -.316 | -.196 | -.245 | -.196 | -.259 | -.196 |
| 2000 | -.270 | -.213 | -.311 | -.201 | -.252 | -.201 | -.262 | -.201 |
| 2001 | -.272 | -.218 | -.316 | -.206 | -.255 | -.207 | -.267 | -.206 |
| 2002 | -.279 | -.217 | -.323 | -.206 | -.259 | -.207 | -.272 | -.206 |
| 2003 | -.267 | -.208 | -.308 | -.197 | -.244 | -.197 | -.260 | -.197 |
| 2004 | -.275 | -.210 | -.311 | -.199 | -.248 | -.199 | -.258 | -.199 |
| 2005 | -.270 | -.210 | -.302 | -.197 | -.250 | -.200 | -.261 | -.197 |
| 2006 | -.273 | -.208 | -.300 | -.196 | -.253 | -.198 | -.261 | -.196 |
| 2007 | -.276 | -.204 | -.303 | -.189 | -.250 | -.191 | -.259 | -.189 |
| 2008 | -.283 | -.206 | -.305 | -.192 | -.257 | -.194 | -.261 | -.192 |
| 2009 | -.273 | -.199 | -.297 | -.186 | -.252 | -.188 | -.258 | -.186 |
| 2010 | -.272 | -.194 | -.292 | -.183 | -.256 | -.185 | -.256 | -.183 |
| 2011 | -.248 | -.170 | -.235 | -.135 | -.226 | -.147 | -.197 | -.135 |
| 2012 | -.234 | -.164 | -.215 | -.127 | -.216 | -.139 | -.187 | -.127 |
| 2013 | -.231 | -.164 | -.207 | -.124 | -.215 | -.135 | -.180 | -.124 |
| 2014 | -.240 | -.165 | -.211 | -.126 | -.229 | -.136 | -.184 | -.126 |
| 2015 | -.241 | -.168 | -.206 | -.130 | -.234 | -.139 | -.185 | -.130 |

**Supplementary Table 17.** East German Trends in Gender Differences in Earnings

| | All Workplaces | | | | Gender-integrated Jobs Only | | | |
|---|---|---|---|---|---|---|---|---|
| | Basic Adj. | Fixed Effect for: | | | Basic Adj. | Fixed Effect for: | | |
| Year | | Est | Occ | Occ-Est | | Est | Occ | Occ-Est |
| 1993 | -.109 | -.120 | -.190 | -.119 | -.049 | -.113 | -.145 | -.119 |
| 1994 | -.108 | -.120 | -.194 | -.112 | -.047 | -.109 | -.141 | -.112 |
| 1995 | -.096 | -.115 | -.198 | -.109 | -.047 | -.107 | -.145 | -.109 |
| 1996 | -.105 | -.119 | -.195 | -.111 | -.065 | -.105 | -.145 | -.111 |
| 1997 | -.102 | -.115 | -.192 | -.109 | -.070 | -.105 | -.141 | -.109 |
| 1998 | -.110 | -.120 | -.190 | -.105 | -.060 | -.104 | -.127 | -.105 |
| 1999 | -.107 | -.119 | -.197 | -.110 | -.058 | -.109 | -.133 | -.110 |
| 2000 | -.102 | -.126 | -.189 | -.118 | -.068 | -.117 | -.142 | -.118 |
| 2001 | -.105 | -.131 | -.202 | -.120 | -.060 | -.117 | -.139 | -.120 |
| 2002 | -.104 | -.132 | -.204 | -.125 | -.068 | -.124 | -.150 | -.125 |
| 2003 | -.096 | -.126 | -.181 | -.116 | -.055 | -.117 | -.136 | -.116 |
| 2004 | -.113 | -.130 | -.196 | -.120 | -.067 | -.123 | -.142 | -.120 |
| 2005 | -.118 | -.135 | -.199 | -.121 | -.070 | -.127 | -.141 | -.121 |
| 2006 | -.116 | -.128 | -.196 | -.122 | -.080 | -.127 | -.144 | -.122 |
| 2007 | -.131 | -.126 | -.204 | -.113 | -.089 | -.124 | -.141 | -.113 |
| 2008 | -.128 | -.125 | -.195 | -.110 | -.095 | -.117 | -.136 | -.110 |

| | | | | | | | | |
|---|---|---|---|---|---|---|---|---|
| 2009 | -.121 | -.121 | -.181 | -.106 | -.094 | -.113 | -.133 | -.106 |
| 2010 | -.118 | -.126 | -.171 | -.115 | -.106 | -.121 | -.142 | -.115 |
| 2011 | -.095 | -.109 | -.143 | -.086 | -.074 | -.094 | -.099 | -.086 |
| 2012 | -.078 | -.103 | -.119 | -.072 | -.075 | -.082 | -.109 | -.072 |
| 2013 | -.097 | -.108 | -.126 | -.078 | -.087 | -.088 | -.117 | -.078 |
| 2014 | -.110 | -.107 | -.140 | -.079 | -.095 | -.088 | -.116 | -.079 |
| 2015 | -.109 | -.108 | -.137 | -.082 | -.100 | -.091 | -.116 | -.082 |

**Supplementary Table 18.** West German Trends in Gender Differences in Earnings

| | All Workplaces | | | | Gender-integrated Jobs Only | | | |
|---|---|---|---|---|---|---|---|---|
| | Basic Adj. | Fixed Effect for: | | | Basic Adj. | Fixed Effect for: | | |
| Year | | Est | Occ | Occ-Est | | Est | Occ | Occ-Est |
| 1993 | -.320 | -.247 | -.339 | -.229 | -.273 | -.224 | -.271 | -.229 |
| 1994 | -.317 | -.243 | -.333 | -.225 | -.272 | -.221 | -.267 | -.225 |
| 1995 | -.317 | -.239 | -.334 | -.220 | -.270 | -.217 | -.266 | -.220 |
| 1996 | -.303 | -.230 | -.322 | -.218 | -.262 | -.213 | -.261 | -.218 |
| 1997 | -.297 | -.226 | -.311 | -.210 | -.260 | -.206 | -.257 | -.210 |
| 1998 | -.295 | -.239 | -.311 | -.225 | -.274 | -.227 | -.271 | -.225 |
| 1999 | -.292 | -.232 | -.309 | -.218 | -.263 | -.218 | -.264 | -.218 |
| 2000 | -.295 | -.236 | -.310 | -.222 | -.271 | -.222 | -.267 | -.222 |
| 2001 | -.294 | -.240 | -.313 | -.225 | -.275 | -.227 | -.272 | -.225 |
| 2002 | -.300 | -.237 | -.321 | -.225 | -.277 | -.226 | -.275 | -.225 |
| 2003 | -.288 | -.228 | -.308 | -.215 | -.262 | -.215 | -.263 | -.215 |
| 2004 | -.291 | -.229 | -.308 | -.217 | -.264 | -.216 | -.262 | -.217 |
| 2005 | -.285 | -.227 | -.299 | -.215 | -.270 | -.217 | -.267 | -.215 |
| 2006 | -.288 | -.226 | -.299 | -.212 | -.271 | -.213 | -.267 | -.212 |
| 2007 | -.290 | -.221 | -.300 | -.205 | -.266 | -.205 | -.264 | -.205 |
| 2008 | -.300 | -.223 | -.307 | -.210 | -.272 | -.210 | -.269 | -.210 |
| 2009 | -.290 | -.216 | -.302 | -.203 | -.268 | -.204 | -.269 | -.203 |
| 2010 | -.289 | -.209 | -.300 | -.198 | -.269 | -.199 | -.265 | -.198 |
| 2011 | -.268 | -.184 | -.237 | -.146 | -.240 | -.158 | -.205 | -.146 |
| 2012 | -.258 | -.177 | -.221 | -.138 | -.231 | -.150 | -.193 | -.138 |
| 2013 | -.251 | -.176 | -.211 | -.134 | -.226 | -.146 | -.184 | -.134 |
| 2014 | -.257 | -.176 | -.211 | -.134 | -.237 | -.144 | -.186 | -.134 |
| 2015 | -.257 | -.181 | -.208 | -.139 | -.241 | -.148 | -.188 | -.139 |

**Supplementary Figure 6.** Trends in German Gender Differences in Earnings

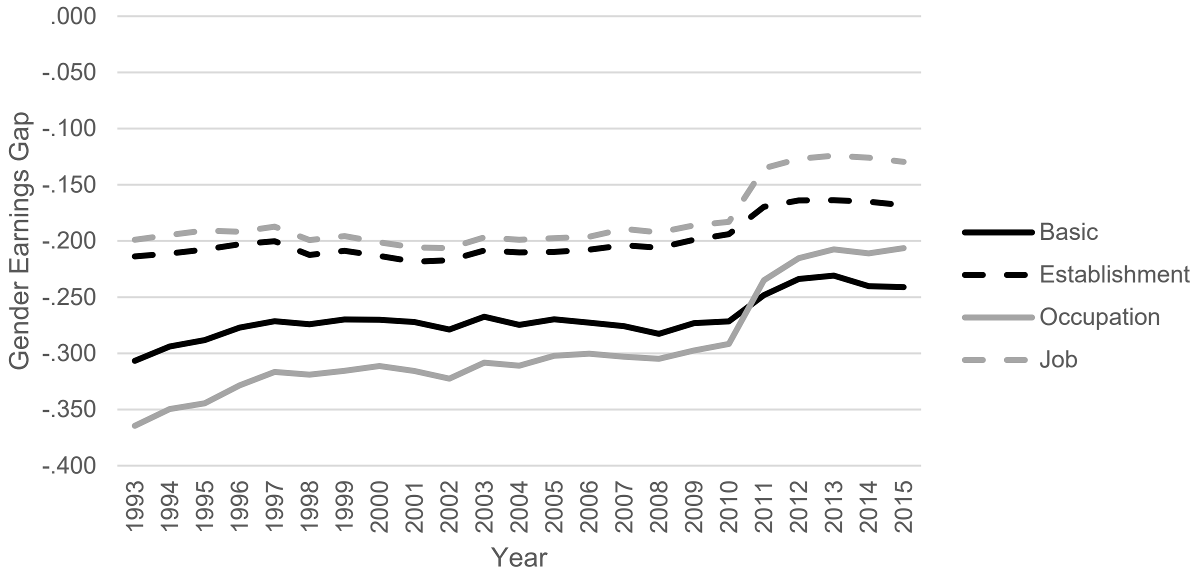


**Supplementary Figure 7.** Trends in East German Gender Differences in Earnings

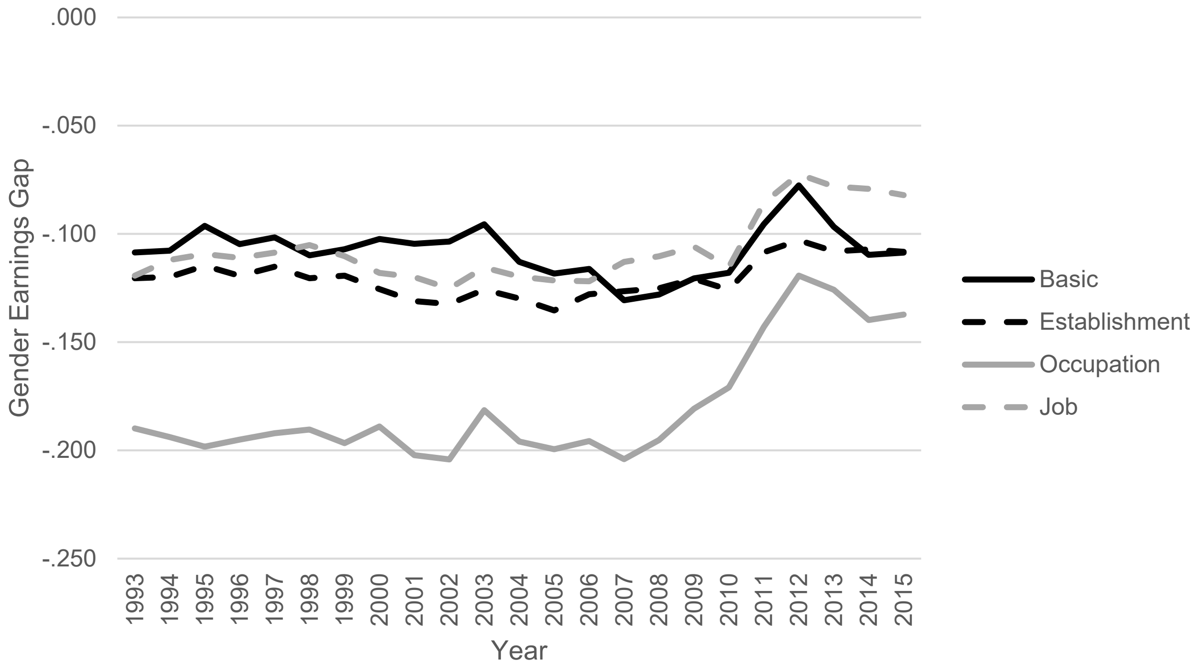

**Supplementary Figure 8.** Trends in West German Gender Differences in Earnings

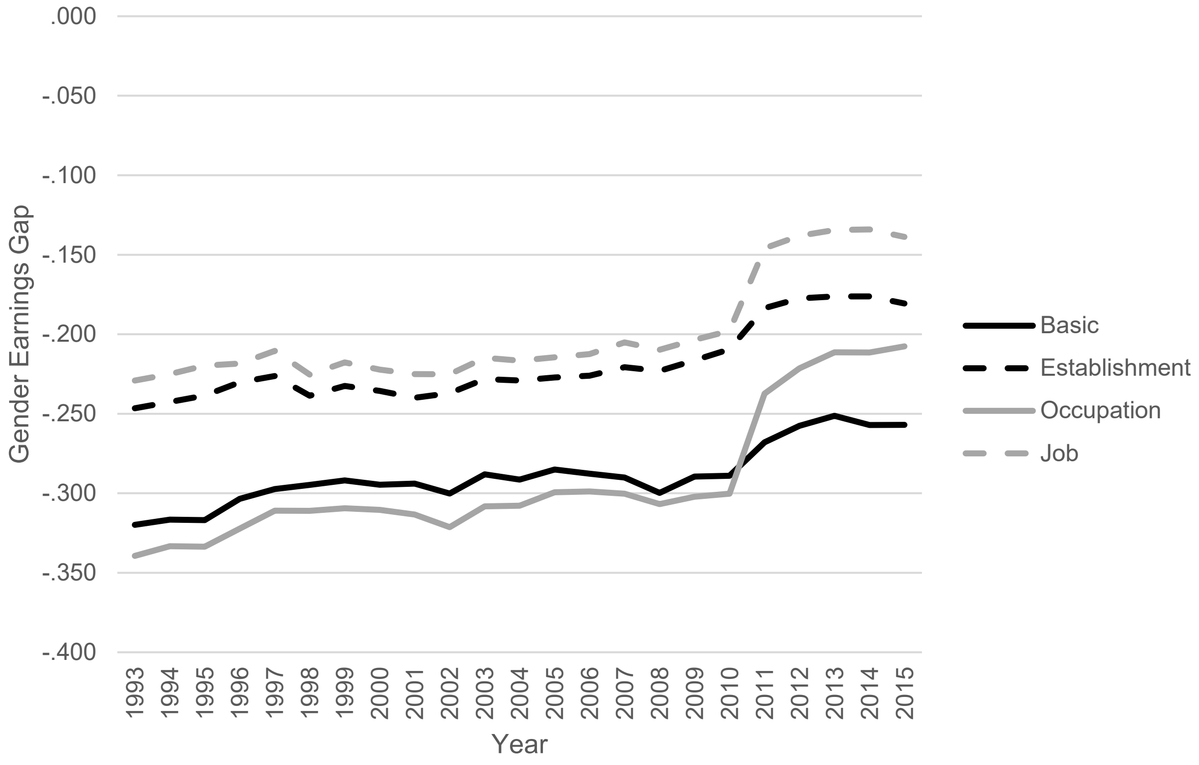


***Hungary***. Our analyses use data processed by the Databank of the Centre for Economic and Regional Studies. These data are generated by linking data from five governmental institutions (the Pension Directorate, the Tax Office, the Health Insurance Fund, the Office of Education, and the Public Employment Service). The data are a 50 percent random sample of the Hungarian population followed from 2003 to 2017. The earnings concept is monthly earnings from each person's primary job. Low-wage job-spells, defined as job-spells earning less than half of the lowest earnings decile in a given year, are dropped from the sample.

We measure occupations using the Hungarian FEOR system, which is very similar to ISCO, so that our measure of occupation can be thought of as four-digit ISCO codes. We are unable to disaggregate individual establishments within employers, and use an anonymized employer identifier as our proxy for establishment. We also lack a measure of education, but derive a three-category proxy using the educational requirements of the occupations that an individual has worked in. For example, if a person has previously worked in a position that requires a higher education degree, this person is coded as having completed higher education.

As the Hungarian data are from a 50 percent random sample of the population, in small firms with only a few workers of either gender we may not observe both women and men, and so gender-integrated firms might appear to include only women (or only men). Therefore gender-integrated

firms and occupation-firm units in our data may not reflect the population of gender-integrated firms and occupation-firm units. If certain sectors have firms or jobs with fewer women (or men) than other sectors, these sectors may be underrepresented in the estimation of the average gender wage gap. To address this, we use post-stratification weights in our analyses. We created weights using the combination of gender, year, 2-digit sector, and 3-digit occupation codes. In each cell the weights are given by the ratio of the total number of workers divided by the number in gender-integrated firms. These weights provide estimates of the gender gap that better reflect the original distribution of workers across sector-occupation cells in that year. To the degree that the weights are effective, we can interpret the differences between the Basic Adjustment results in Tables 1 and 6 as attributable to sorting into gender-segregated units. Of note, Table 6 reports a larger gender difference in the Basic Adjustments model than in Table 1, so that where Table 1 shows that nearly all of the gender earnings inequality we observe is due to within-job differences, in Table 6 we see that only about half of the gender earnings inequality among those working in gender-integrated jobs is due to within-job differences.

Our data allow us to examine how gender differences have changed from 2003-2017. Supplementary Table 19 and Supplementary Figure 9 present information on the trends in the Hungarian gender gap overall, as well as within establishments, occupations, and jobs. Supplementary Table 19 also reports results from models estimated using a consistent sample of gender-integrated jobs in each year. Data similar to those used for this project can be accessed at the Databank of the Centre for Economic and Regional Studies upon receipt of proper authorizations.

**Supplementary Table 19.** Hungarian Trends in Gender Differences in Earnings

| | All Workplaces | | | | Gender-integrated Jobs Only | | | |
|---|---|---|---|---|---|---|---|---|
| | Basic Adj. | Fixed Effect for: | | | Basic Adj. | Fixed Effect for: | | |
| Year | | Est | Occ | Occ-Est | | Est | Occ | Occ-Est |
| 2003 | -.048 | -.102 | -.097 | -.085 | -.133 | -.080 | -.124 | -.085 |
| 2004 | -.083 | -.118 | -.108 | -.091 | -.181 | -.102 | -.136 | -.091 |
| 2005 | -.065 | -.112 | -.104 | -.091 | -.170 | -.096 | -.133 | -.091 |
| 2006 | -.083 | -.113 | -.110 | -.091 | -.171 | -.101 | -.133 | -.091 |
| 2007 | -.103 | -.113 | -.117 | -.092 | -.180 | -.100 | -.136 | -.092 |
| 2008 | -.099 | -.112 | -.115 | -.096 | -.178 | -.101 | -.139 | -.096 |
| 2009 | -.113 | -.100 | -.110 | -.091 | -.190 | -.088 | -.132 | -.091 |
| 2010 | -.098 | -.080 | -.091 | -.076 | -.167 | -.061 | -.114 | -.076 |
| 2011 | -.102 | -.101 | -.083 | -.077 | -.196 | -.102 | -.126 | -.077 |
| 2012 | -.101 | -.105 | -.079 | -.080 | -.200 | -.112 | -.127 | -.080 |
| 2013 | -.122 | -.129 | -.104 | -.093 | -.234 | -.132 | -.147 | -.093 |
| 2014 | -.108 | -.128 | -.105 | -.095 | -.222 | -.136 | -.147 | -.095 |
| 2015 | -.110 | -.130 | -.107 | -.096 | -.227 | -.139 | -.150 | -.096 |
| 2016 | -.104 | -.132 | -.106 | -.099 | -.222 | -.141 | -.152 | -.099 |
| 2017 | -.099 | -.130 | -.098 | -.095 | -.197 | -.138 | -.142 | -.095 |

**Supplementary Figure 9.** Trends in Hungarian Gender Differences in Earnings

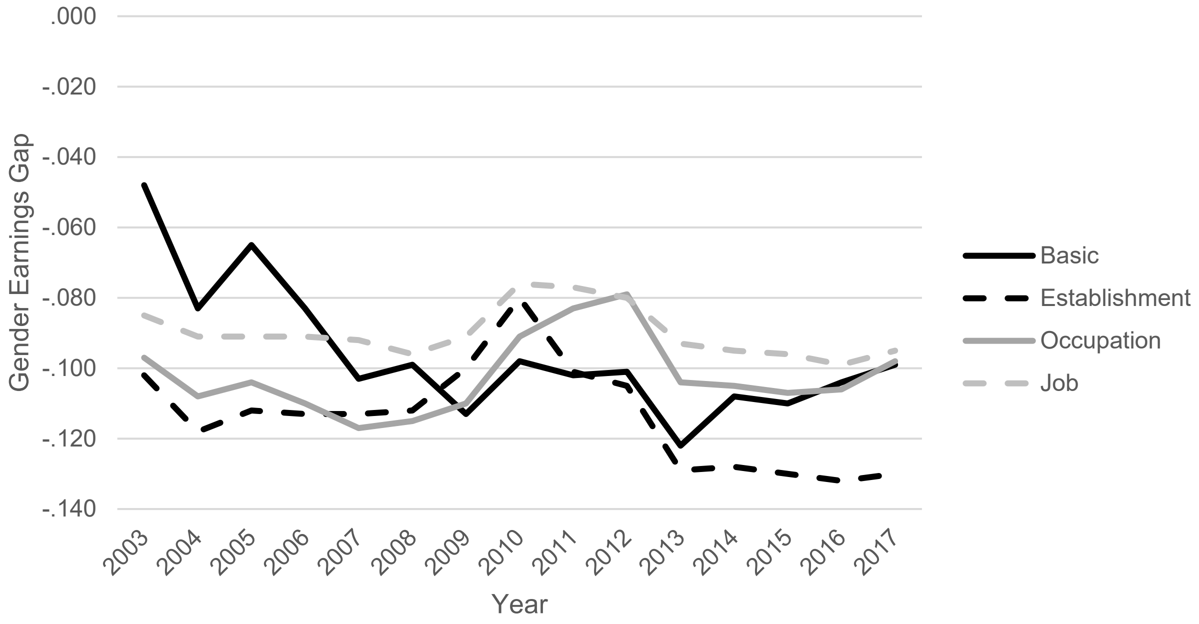


***Israel***. Our analyses use data from the administrative longitudinal employer-employee panel (LEEP) generated by the Israeli Central Bureau of Statistics (CBS) from 2001 to 2015. These data include monthly earnings, demographic, educational, and employer information for each employment spell for each individual. For individuals who work at multiple establishments in a year, we use information from their highest-earning job. The Israeli LEEP data was top-coded by the CBS at the 95 percentile; we imputed the censored part of the earnings distribution in each year using a tobit model that reflects individual and workplace-specific components of earnings (*9*).

As the LEEP data do not contain occupation information, we link these data to Labor Force Surveys (LFS), which cover about a one percent random sample of Israeli households. The LFS ask respondents to self-report their current primary or most recent primary occupation at the time of the survey. In the years 2001 to 2010, self-reported occupations coded by CBS coders into one of approximately 417 3-digit categories from the Standard Classification of Occupations 1994. Since 2012, self-reported occupations coded into about 500 3-digit categories from the Standard Classification of Occupations 2011, which is based on the International Standard Classification of Occupations ISCO-08. We use a crosswalk between 2011 and 1994 codes to create a consistent set of 360 occupations matching the 1994 and 2011 codes. As we only have information on one percent of employees within each firm, we collapse these into two-digit codes for our analyses.

We also derive information on hours worked from the LFS, multiplying weekly hours worked by 4.2 to obtain the total monthly number of hours worked. We then divide total monthly earnings by

monthly hours worked to arrive at an estimation of hourly earnings in a typical week, which we use as our proxy for hourly wage.

Our data allow us to examine how gender differences have changed from 2001-2015. Supplementary Table 20 and Supplementary Figure 10 present information on the trends in the Israeli gender gap overall, as well as within establishments, occupations, and jobs. Supplementary Table 20 also reports results from models estimated using a consistent sample of gender-integrated jobs in each year. Data similar to those used for this project can be accessed at Israel's Central Bureau of Statistics (CBS) upon receipt of proper authorizations.

**Supplementary Table 20.** Israeli Trends in Gender Differences in Earnings

| | All Workplaces | | | | Gender-integrated Jobs Only | | | |
|---|---|---|---|---|---|---|---|---|
| | Basic Adj. | Fixed Effect for: | | | Basic Adj. | Fixed Effect for: | | |
| Year | | Est | Occ | Occ-Est | | Est | Occ | Occ-Est |
| 2001 | -.382 | -.282 | -.267 | -.204 | -.327 | -.210 | -.215 | -.204 |
| 2002 | -.326 | -.219 | -.227 | -.140 | -.233 | -.148 | -.154 | -.140 |
| 2003 | -.339 | -.232 | -.253 | -.158 | -.227 | -.148 | -.154 | -.158 |
| 2004 | -.335 | -.245 | -.204 | -.124 | -.263 | -.140 | -.140 | -.124 |
| 2005 | -.334 | -.249 | -.214 | -.144 | -.233 | -.150 | -.168 | -.144 |
| 2006 | -.320 | -.202 | -.213 | -.133 | -.237 | -.139 | -.139 | -.133 |
| 2007 | -.310 | -.200 | -.205 | -.139 | -.219 | -.146 | -.135 | -.139 |
| 2008 | -.289 | -.181 | -.176 | -.127 | -.202 | -.123 | -.128 | -.127 |
| 2009 | -.284 | -.222 | -.188 | -.138 | -.215 | -.150 | -.147 | -.138 |
| 2010 | -.297 | -.227 | -.202 | -.156 | -.246 | -.156 | -.169 | -.156 |
| 2011 | -.267 | -.267 | -.267 | -.267 | -.327 | -.327 | -.327 | -.327 |
| 2012 | -.338 | -.192 | -.207 | -.110 | -.230 | -.125 | -.108 | -.110 |
| 2013 | -.357 | -.195 | -.199 | -.120 | -.280 | -.143 | -.143 | -.120 |
| 2014 | -.363 | -.195 | -.192 | -.074 | -.269 | -.110 | -.087 | -.074 |
| 2015 | -.336 | -.197 | -.196 | -.119 | -.290 | -.164 | -.131 | -.119 |

**Supplementary Figure 10.** Trends in Israeli Gender Differences in Earnings

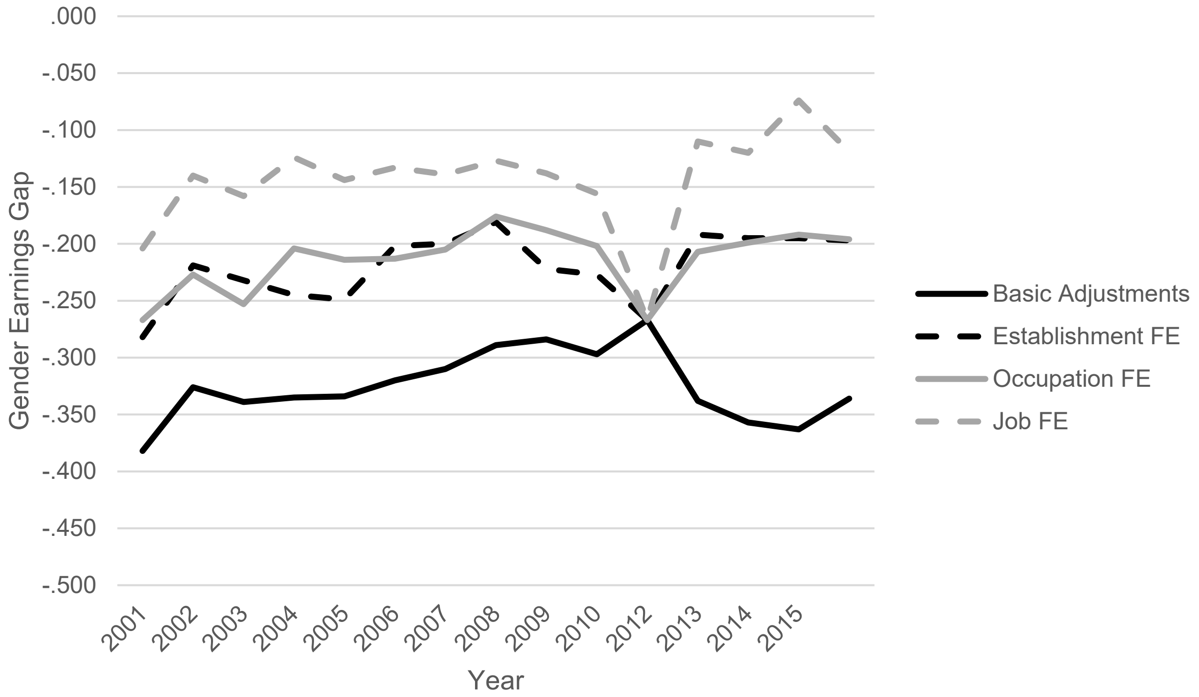


***Japan***. Our analyses use data from the Basic Survey on Wage Structures (BSWS), the most comprehensive wage survey in Japan, which is conducted every year by the Ministry of Health, Labour and Welfare. The BSWS covers almost all industries except agriculture, forestry, fisheries, and public services. It covers private- and public-sector firms with ten or more employees, and private-sector establishments with more than five employees. The establishments in the sample are randomly chosen in proportion to the size of prefectures, industries, and the number of employees. The sampling for the survey was implemented in two steps: first, a random sample of establishments was selected from the Establishment and Enterprise Census, which covers all establishments in Japan; the establishments selected in the first step were then asked to take a random sample of workers and provide their payroll records. All large establishments are sampled, but the threshold for selection based on the establishment's number of employees varies by industry. Smaller establishments are selected with a decreasing sampling probability based on the number of employees. By contrast, the selection probabilities of employees from large establishments are low, and those from small establishments are high.

These data are collected once a year in July and contain information on individual workers' monthly salaries in June, the total bonus payments in the previous year, hours worked, gender, age, length of employment, education, job title, and job type. The data include approximately 1.2 million workers for each year, from 70,000 establishments in the period 1989-2013. Annual earnings

include wages (regular and overtime) and annual bonuses. Our measure of hourly earnings is calculated by dividing monthly earnings (annual earnings divided by 12) by monthly working hours.

Our occupation measure is constructed from variables for managerial status (managers in firms with over 100 employees were classified as directors, managers, section heads, and foremen; ten percent of the sample), blue collar/white collar flags in manufacturing establishments (eight and six percent, respectively), and occupation codes for the 45 percent of workers with specific skills (e.g., teachers, architects, programmers). The 40 percent who are missing occupation codes are considered general office workers and are assigned to their own occupation code. Education is measured using four categories: 1) junior high school graduates (who obtained nine years of education); 2) senior high school graduates (twelve years of education); 3) college or technical college graduates (fourteen years of education); and 4) university graduates or higher (16+ years of education). Part-time workers are not required to provide information about education, so we assume that part-time workers are high school graduates.

Our data allow us to examine how gender differences have changed from 1989 to 2013. Supplementary Table 21 and Supplementary Figure S11 present information on the trends in the Japanese gender gap overall, as well as within establishments, occupations, and jobs. Supplementary Table 21 also reports results from models estimated using a consistent sample of gender-integrated jobs in each year. Data similar to those used for this project can be accessed at the Ministry of Health, Labour and Welfare upon the approval of an application by the relevant authorities.

**Supplementary Table 21.** Japanese Trends in Gender Differences in Earnings

| | All Workplaces | | | | Gender-integrated Jobs Only | | | |
|---|---|---|---|---|---|---|---|---|
| | Basic Adj. | Fixed Effect for: | | | Basic Adj. | Fixed Effect for: | | |
| Year | | Est | Occ | Occ-Est | | Est | Occ | Occ-Est |
| 1993 | -.608 | -.488 | -.544 | -.437 | -.540 | -.445 | -.496 | -.437 |
| 1994 | -.584 | -.478 | -.529 | -.427 | -.527 | -.436 | -.487 | -.427 |
| 1995 | -.571 | -.470 | -.519 | -.416 | -.500 | -.427 | -.476 | -.416 |
| 1996 | -.560 | -.464 | -.506 | -.412 | -.494 | -.422 | -.466 | -.412 |
| 1997 | -.556 | -.458 | -.503 | -.402 | -.489 | -.414 | -.461 | -.402 |
| 1998 | -.531 | -.439 | -.477 | -.380 | -.463 | -.391 | -.436 | -.380 |
| 1999 | -.505 | -.427 | -.461 | -.372 | -.440 | -.382 | -.420 | -.372 |
| 2000 | -.477 | -.413 | -.435 | -.358 | -.433 | -.368 | -.410 | -.358 |
| 2001 | -.470 | -.401 | -.432 | -.350 | -.415 | -.358 | -.399 | -.350 |
| 2002 | -.449 | -.394 | -.412 | -.338 | -.400 | -.350 | -.385 | -.338 |
| 2003 | -.445 | -.386 | -.413 | -.333 | -.401 | -.344 | -.386 | -.333 |
| 2004 | -.416 | -.366 | -.378 | -.313 | -.377 | -.324 | -.362 | -.313 |
| 2005 | -.445 | -.396 | -.400 | -.326 | -.402 | -.345 | -.381 | -.326 |
| 2006 | -.453 | -.395 | -.395 | -.322 | -.405 | -.343 | -.375 | -.322 |
| 2007 | -.442 | -.387 | -.390 | -.314 | -.383 | -.332 | -.359 | -.314 |

| | | | | | | | | |
|---|---|---|---|---|---|---|---|---|
| 2008 | -.422 | -.366 | -.364 | -.286 | -.370 | -.306 | -.344 | -.286 |
| 2009 | -.379 | -.352 | -.334 | -.281 | -.339 | -.298 | -.320 | -.281 |
| 2010 | -.370 | -.345 | -.326 | -.274 | -.338 | -.294 | -.312 | -.274 |
| 2011 | -.353 | -.332 | -.313 | -.263 | -.311 | -.282 | -.301 | -.263 |
| 2012 | -.358 | -.323 | -.311 | -.254 | -.326 | -.275 | -.302 | -.254 |
| 2013 | -.350 | -.328 | -.304 | -.257 | -.314 | -.277 | -.295 | -.257 |

**Supplementary Figure 11.** Trends in Japanese Gender Differences in Earnings

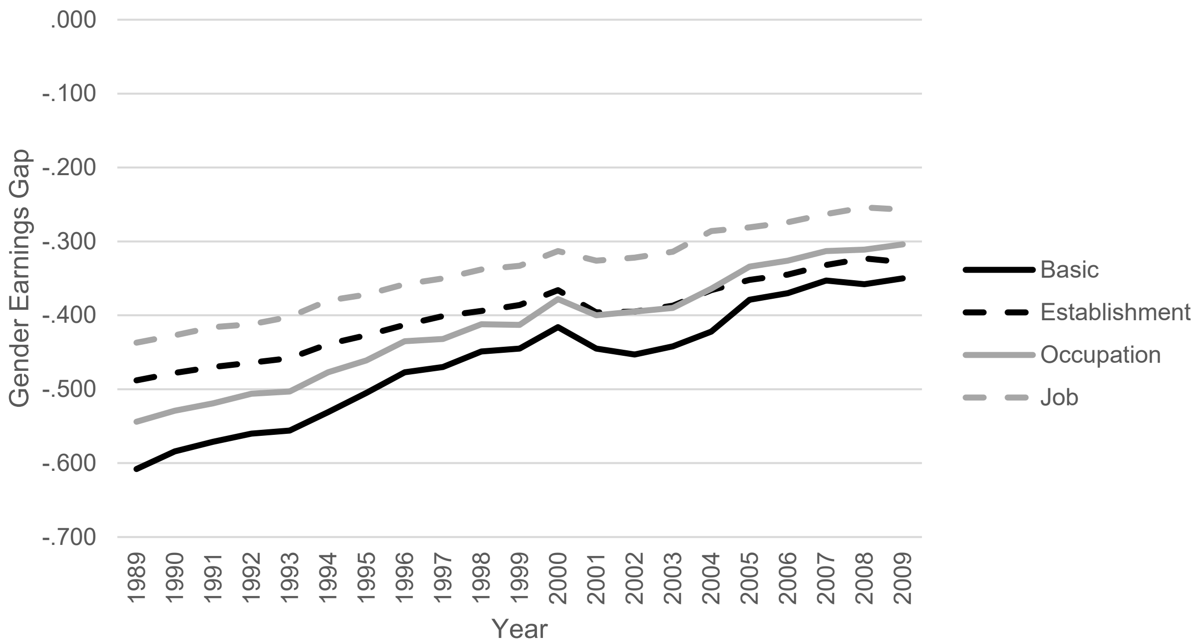


***Netherlands***. Our analyses use data on the Dutch population 16 years or older from the Dutch Labor Force survey (*Enquete Beroepsbevolking*, EBB) in the period 2006-2014. The EBB contains information on education and occupation, and it is linked to the Social Statistics Database (*Sociaal Statistisch Bestand*, SSB) of the Central Bureau of Statistics of Netherlands, a system of linked municipal, vital, and educational registers, and social and employment insurance administration (*Polisadministratie*). The micro-level registry data contain complete population information on age, gender, monthly salaries and contractual working hours for all jobs held in a given year, and it identifies employers. The EBB has a quarterly rotating panel design: in each quarter, it surveys a roughly one percent sample of the Dutch population and administers a follow-up survey to the respondents who participated in the survey in the previous quarter. Each individual stays in the panel for a maximum period of 12 months. The sampling method of the EBB is a two-step stratified household sample: in the first step a stratified sample of municipalities was taken, followed by a systematic random sample of addresses within each municipality. The total number of respondents in the EBB ranges from 122,312 to 165,966, depending on the year. As the Central Bureau of

Statistics draws the household sample for the EBB from the municipal registers, almost all EBB respondents can be matched to register data (e.g., in 2006, 99.1 percent of EBB respondents were matched to the SSB).

Wages are calculated using the contractual yearly wage from a given job excluding bonus payments, cash benefits, and overtime pay, divided by the number of hours worked to arrive at hourly base wage. Earnings are measured with the total cash earnings which include annual bonus payments and overtime pay divided by the number of months employed in a given job to adjust for variation in job spells (e.g., job changes and seasonal work). The EBB measures occupations coded into ISCO 2008 codes. We used the 3-digit version, as the 4-digit version was not feasible for the job-fixed-effects specification due to limited sample sizes within establishments. When individuals have more than one occupation code for a primary job recorded (e.g., they change jobs during the survey window), we use information from their first recorded occupation.

Our measure of education (ISCED 2011) is gathered from the digital administration of educational institutions for recent cohorts of graduates, supplemented by self-reported education data from surveys linked with the SSB for older cohorts. As the Dutch Central Bureau of Statistics primarily uses the EBB to supplement missing information on education for older cohorts, there is a close to perfect overlap between the EBB sample and education sample (e.g. 99.7 percent in 2006). In total, we have education information for about two-thirds of all Dutch workers under the age of 50.

As discussed above, the household-based sample results in the underrepresentation of smaller and relatively gender-segregated establishments. We corrected for the bias this may generate by weighting the sample distribution of workplace gender composition to match the population-level distribution of workplace gender composition (from the SSB). We use workplace gender composition quintiles for the total population of workers to create weights for our baseline and occupation fixed effects comparisons, and the workplace gender composition distribution for the universe of gender-integrated workplaces to create weights for our establishment and job fixed effect models.

Our data allow us to examine how gender differences have changed from 2006-2014. Supplementary Table 22 and Supplementary Figure 12 present information on the trends in the Dutch gender gap overall, as well as within establishments, occupations, and jobs. Supplementary Table 22 also reports results from models estimated using a consistent sample of gender-integrated jobs in each year. Note the Central Bureau of Statistics modified the universe of jobs included in their jobs and wages database (SPOLISBUS) between 2009 and 2010, so that care should be exercised when comparing results from before and after this change. The most notable change is that beginning in 2010 data exclude volunteer positions where paid compensation does not reach the taxability threshold. Data similar to those used for this project can be accessed at Central Bureau of Statistics of the Netherlands upon receipt of proper authorizations.

**Supplementary Table 22.** Dutch Trends in Gender Differences in Earnings

| | All Workplaces | | | | Gender-integrated Jobs Only | | | |
|---|---|---|---|---|---|---|---|---|
| | Basic Adj. | Fixed Effect for: | | | Basic Adj. | Fixed Effect for: | | |
| Year | | Est | Occ | Occ-Est | | Est | Occ | Occ-Est |
| 2006 | -0.324 | -0.215 | -0.179 | -0.163 | -0.195 | -0.174 | -0.147 | -0.163 |
| 2007 | -0.353 | -0.225 | -0.209 | -0.178 | -0.265 | -0.193 | -0.170 | -0.178 |
| 2008 | -0.357 | -0.231 | -0.209 | -0.195 | -0.265 | -0.206 | -0.191 | -0.195 |
| 2009 | -0.345 | -0.218 | -0.187 | -0.158 | -0.223 | -0.167 | -0.162 | -0.158 |
| 2010 | -0.285 | -0.193 | -0.148 | -0.129 | -0.206 | -0.145 | -0.111 | -0.129 |
| 2011 | -0.284 | -0.179 | -0.156 | -0.129 | -0.216 | -0.143 | -0.119 | -0.129 |
| 2012 | -0.23 | -0.164 | -0.12 | -0.132 | -0.194 | -0.136 | -0.124 | -0.132 |
| 2013 | -0.182 | -0.146 | -0.092 | -0.121 | -0.15 | -0.124 | -0.102 | -0.121 |
| 2014 | -0.202 | -0.146 | -0.111 | -0.075 | -0.126 | -0.083 | -0.078 | -0.075 |

**Supplementary Figure 12.** Trends in Dutch Gender Differences in Earnings

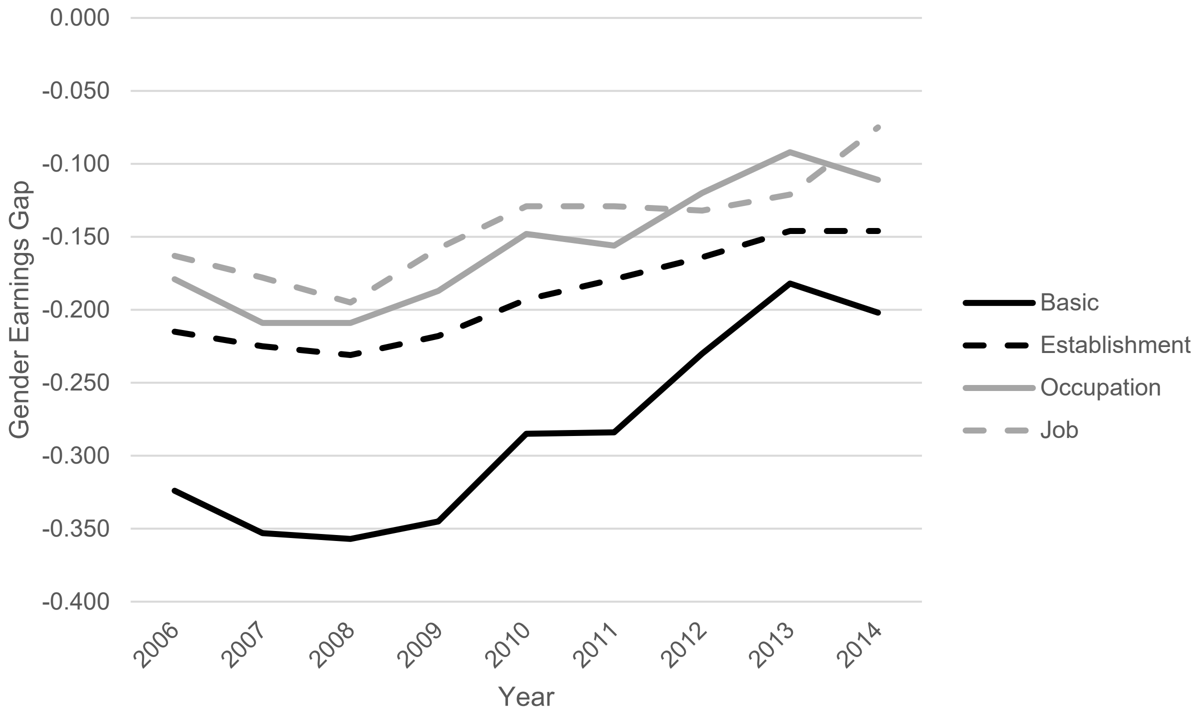


***Norway***. Our analyses use data from Statistics Norway's wage statistics in the period 1997-2018 for information on contractual monthly salaries, contractual hours worked, part- versus full-time status, occupation, and identifying employers. Between 1997 and 2014 these data were collected once per year in the fall (with some variation across sectors) on all job observations in the public sector and a large representative sample of approximately 70 percent of jobs in the private sector.

Beginning in 2015, all firms, establishments, and job observations in the private sector are included so that our data covers the entire public and private sectors in the Norwegian labor market.

For the 1997-2014 period, the private sector sample is drawn from the population of all firm records in Statistics Norway's register on establishments and firms (i.e., *Bedrifts- og foretaksregister*). Firms in the agricultural and forestry industries are excluded, while firms in the fishing industry were included beginning in 2002. In the private sector sample, the sampling unit is at the level of firms and all establishments within a firm are grouped together as one employer unit. All individuals in each sampled firm are included in the data. The private sector sample is stratified by both industry and number of employees. All large employers are sampled, but the threshold for selection based on the firm's number of employees varies by industry. Smaller firms are selected with a decreasing sampling probability based on the number of employees. Beginning in 2015, our sample includes all firms, establishments, and job observations in the private sector, as well as the entire public sector, drawn from an expanded version of Statistics Norway's wage statistics (i.e., *A-ordningen*).

Our measure of hourly wages is based on information on contractual monthly salaries and contractual hours worked at the time of registration each year. Monthly salary information is based on contractual regular earnings per month and does not include bonuses, nonregular extra pay, or overtime pay. In the private sector, hours worked is based on information on contractual hours worked per week. In the public sector, hours worked is based on information on the percent of full-time hours of employment (i.e., it measures the individual's contractual work hours as the percent of regular full-time work, ranging between zero and 100). The measure of earnings comes from tax records, and includes all work-related income (such as parental and sick leave benefits; but not unemployment benefits) for each year and is captured with high accuracy.

We merge the earnings data to the wage statistics sample in order to get information on occupation and on contractual work hours to create our indicator of full- versus part-time work. Information on occupation is based on Statistics Norway's four-digit Norwegian version of ISCO-88 (i.e., *Standard for yrkesklassifisering, STYRK98*), as well as on separate occupational title systems for various public sector employers (i.e., occupational titles from *Statens tjenestemannsregister*, *PAI registeret*, and *Maritimt register*). Starting in 2008, the occupational codes for all individuals and employers use the four-digit Norwegian version of ISCO-88. For analyses examining the factors behind the differences between our results and those in previous analyses, we also use a more detailed seven-digit measure of occupational titles available from 2006 to 2018. For individuals who work multiple jobs and thus have multiple job observations per year, we use information from their job observation with the highest contractual monthly salary.

Information about gender and age is based on records from the Central Population Register. Information about education refers to each individual's highest level of educational qualifications in

each year based on annual records from the National Education Database (i.e., *Nasjonal utdanningsdatabase, NUDB*). Information on educational level is measured using the eight category NUS2000 scale (i.e., the Norwegian version of ISCED-97), ranging from primary education (1) to doctoral level degree (8). Observations registered with no education (0) or missing education (9) are excluded (these observations represent less than one percent of the data).

Our data allow us to examine how gender differences have changed from 1997 to 2018. Supplementary Table 23 and Supplementary Figure 13 present information on the trends in the Norwegian gender gap overall, as well as within establishments, occupations, and jobs. Supplementary Table 23 also reports results from models estimated using a consistent sample of gender-integrated jobs in each year. Data similar to those used for this project can be accessed at Statistics Norway upon receipt of proper authorizations.

**Supplementary Table 23.** Norwegian Trends in Gender Differences in Earnings

| | All Workplaces | | | | Gender-integrated Jobs Only | | | |
|---|---|---|---|---|---|---|---|---|
| | Basic Adj. | Fixed Effect for: | | | Basic Adj. | Fixed Effect for: | | |
| Year | | Est | Occ | Occ-Est | | Est | Occ | Occ-Est |
| 1997 | -.236 | -.201 | -.166 | -.136 | -.209 | -.174 | -.157 | -.136 |
| 1998 | -.247 | -.203 | -.171 | -.140 | -.215 | -.175 | -.159 | -.140 |
| 1999 | -.237 | -.192 | -.164 | -.134 | -.209 | -.169 | -.155 | -.134 |
| 2000 | -.233 | -.189 | -.164 | -.133 | -.206 | -.166 | -.156 | -.133 |
| 2001 | -.229 | -.183 | -.148 | -.123 | -.201 | -.160 | -.143 | -.123 |
| 2002 | -.228 | -.181 | -.147 | -.118 | -.192 | -.146 | -.138 | -.118 |
| 2003 | -.221 | -.177 | -.148 | -.120 | -.185 | -.144 | -.138 | -.120 |
| 2004 | -.219 | -.173 | -.144 | -.112 | -.181 | -.139 | -.132 | -.112 |
| 2005 | -.213 | -.165 | -.137 | -.110 | -.178 | -.134 | -.127 | -.110 |
| 2006 | -.221 | -.167 | -.139 | -.111 | -.183 | -.134 | -.129 | -.111 |
| 2007 | -.231 | -.169 | -.148 | -.115 | -.191 | -.139 | -.134 | -.115 |
| 2008 | -.229 | -.165 | -.144 | -.109 | -.189 | -.133 | -.129 | -.109 |
| 2009 | -.216 | -.160 | -.139 | -.105 | -.184 | -.129 | -.125 | -.105 |
| 2010 | -.210 | -.156 | -.137 | -.102 | -.182 | -.126 | -.122 | -.102 |
| 2011 | -.207 | -.154 | -.135 | -.101 | -.180 | -.124 | -.121 | -.101 |
| 2012 | -.207 | -.152 | -.136 | -.100 | -.182 | -.123 | -.121 | -.100 |
| 2013 | -.206 | -.152 | -.133 | -.100 | -.183 | -.124 | -.121 | -.100 |
| 2014 | -.206 | -.149 | -.133 | -.099 | -.183 | -.122 | -.119 | -.099 |
| 2015 | -.216 | -.134 | -.127 | -.093 | -.187 | -.115 | -.115 | -.093 |
| 2016 | -.209 | -.136 | -.122 | -.091 | -.183 | -.112 | -.113 | -.091 |
| 2017 | -.204 | -.131 | -.120 | -.087 | -.176 | -.107 | -.109 | -.087 |
| 2018 | -.206 | -.128 | -.120 | -.086 | -.176 | -.106 | -.108 | -.086 |

**Supplementary Figure 13.** Trends in Norwegian Gender Differences in Earnings

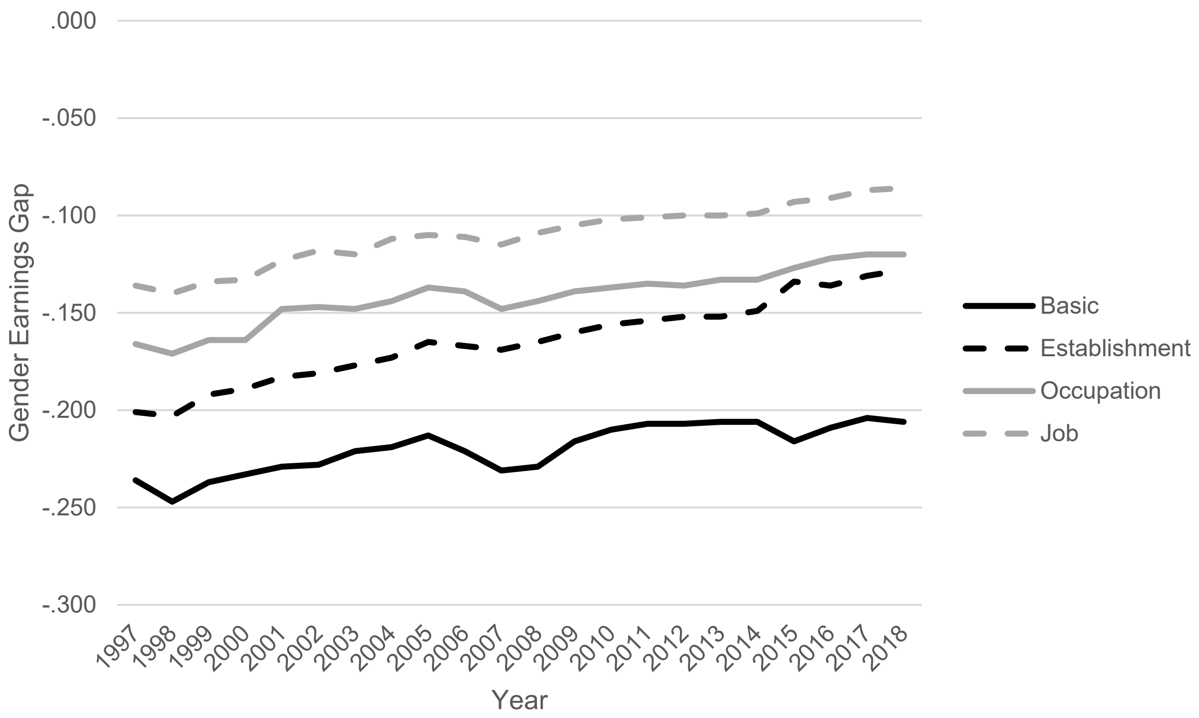


***Slovenia***. Our analyses use data from the register-based Labor Market Statistics (LMS) and the Statistical Register of Employment (SRDAP) for the period from 1999 to 2015. Data are collected by Slovenian Statistical Office and cover the entire Slovenian labor force and registered companies in both the private and public sector across all industries. Firm, establishment, and person identifiers were used to link the databases.

Information about gender, age, working time (contractual), employment relationship (temporary or permanent), and company's economic activity (NACE rev.2) is based on the data from Statistical Register of Employment (SRDAP). Full-time workers in Slovenia are employed for 36 hours or more per week, and part-time workers for less than 36 hours. The data from SRDAP represent the status of individuals on the 31$^{st}$ of December of each year. Information about gross earnings, occupation, and education for the period from 1999 to 2015 is based on records from the LMS. Data on earnings are obtained from the Slovenian financial administration. All taxed incomes earned in a given year are included, and we are unable to distinguish between the various components: wages, wage compensation for periods of absence (e.g., annual leave, paid absence due to personal circumstances, statutory holidays, and sick leave), commission fees, and other taxed incomes (e.g., severance pay, jubilee awards, and other income that is taxed when the threshold defined by the government is exceeded). Information about education refers to each individual's highest level of educational attainment in each year. Educational attainment was measured following ISCED 2011, using seven categories: 1) incomplete basic education; 2) basic education; 3)

short-term vocational upper secondary education; 4) vocational upper secondary education; 5) technical, general upper secondary education; 6) the first cycle of higher education (e.g., BA); 7) second or third stage of higher education (e.g., MA or PhD). Occupation is based on the Slovenian Statistical Office's national standard classification of occupations, which changes over time: in 1999, the four-digit version of ISCO-88 was used; from 2000-2010 six-digit ISCO-88 was used; and from 2011-2015 four-digit ISCO-08 was used. We use crosswalks to harmonize these data to the current four-digit ISCO-08 categories.

Our data allow us to examine how gender differences have changed from 1999 to 2015. Supplementary Table 24 and Supplementary Figure 14 present information on the trends in the Slovenian gender gap overall, as well as within establishments, occupations, and jobs. Supplementary Table 24 also reports results from models estimated using a consistent sample of gender-integrated jobs in each year. Data similar to those used for this project can be accessed at the Slovenian Statistical Office upon receipt of proper authorizations.

**Supplementary Table 24.** Slovenian Trends in Gender Differences in Earnings

| | All Workplaces | | | | Gender-integrated Jobs Only | | | |
|---|---|---|---|---|---|---|---|---|
| | Basic Adj. | Fixed Effect for: | | | Basic Adj. | Fixed Effect for: | | |
| Year | | Est | Occ | Occ-Est | | Est | Occ | Occ-Est |
| 1999 | -.143 | -.162 | -.147 | -.134 | -.179 | -.160 | -.174 | -.134 |
| 2000 | -.150 | -.162 | -.151 | -.136 | -.188 | -.161 | -.178 | -.136 |
| 2001 | -.141 | -.160 | -.145 | -.133 | -.174 | -.157 | -.165 | -.133 |
| 2002 | -.140 | -.158 | -.145 | -.132 | -.172 | -.154 | -.164 | -.132 |
| 2003 | -.145 | -.156 | -.143 | -.130 | -.176 | -.152 | -.161 | -.130 |
| 2004 | -.157 | -.159 | -.151 | -.131 | -.182 | -.154 | -.167 | -.131 |
| 2005 | -.168 | -.164 | -.158 | -.136 | -.187 | -.158 | -.172 | -.136 |
| 2006 | -.178 | -.171 | -.165 | -.143 | -.199 | -.165 | -.180 | -.143 |
| 2007 | -.197 | -.183 | -.177 | -.153 | -.215 | -.177 | -.192 | -.153 |
| 2008 | -.211 | -.189 | -.185 | -.157 | -.230 | -.183 | -.200 | -.157 |
| 2009 | -.172 | -.180 | -.163 | -.149 | -.202 | -.178 | -.183 | -.149 |
| 2010 | -.168 | -.171 | -.153 | -.138 | -.196 | -.168 | -.172 | -.138 |
| 2011 | -.169 | -.166 | -.152 | -.138 | -.200 | -.163 | -.173 | -.138 |
| 2012 | -.175 | -.165 | -.151 | -.136 | -.200 | -.161 | -.169 | -.136 |
| 2013 | -.174 | -.162 | -.147 | -.134 | -.202 | -.159 | -.169 | -.134 |
| 2014 | -.177 | -.164 | -.149 | -.137 | -.207 | -.161 | -.173 | -.137 |
| 2015 | -.190 | -.169 | -.157 | -.140 | -.222 | -.165 | -.181 | -.140 |

**Supplementary Figure 14.** Trends in Slovenian Gender Differences in Earnings

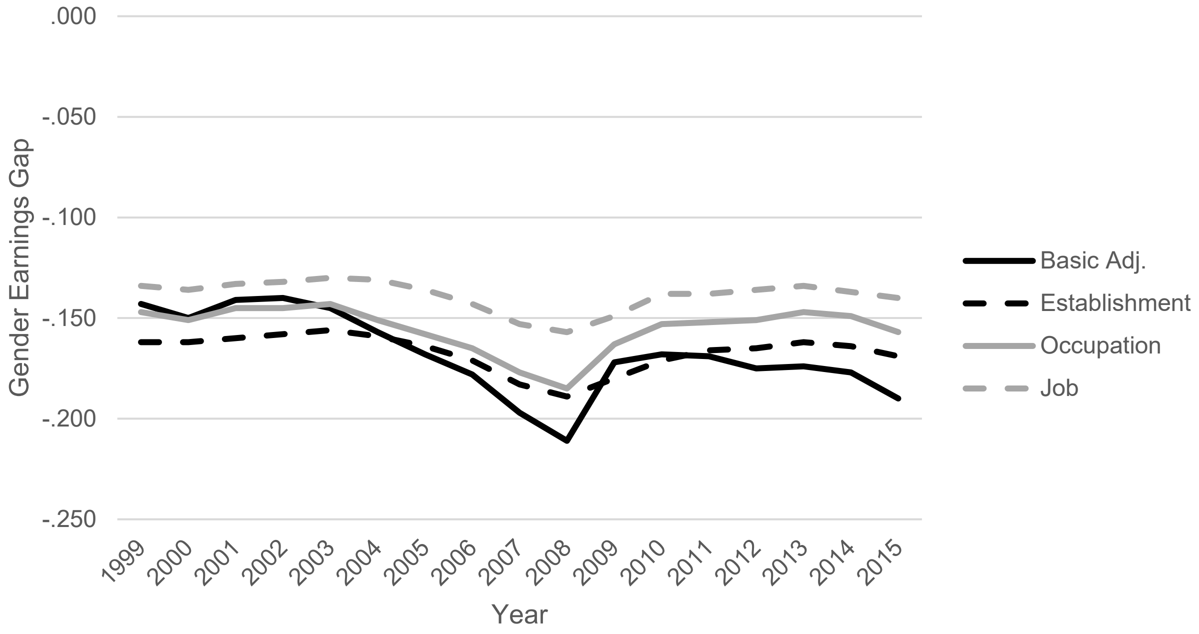


***South Korea***. Our analyses use data from the Korean Ministry of Employment and Labor (MOEL)'s Wage Structure Survey (1982-2004) and Survey on Labor Conditions by Employment Type (2006-2012) for information on hourly wage, occupations, and other characteristics of individual employees, such as gender, age, and education. These data are collected every year in June and provide information on employees at sampled private-sector establishments. We used the establishment identifier and individual employee's identifier to link the database.

The survey sample is drawn from the population of all firms included in the Establishment Status published by MOEL. Prior to 2006, establishments hiring five or more employees were included in the sample; from 2006 on, MOEL expanded the sample by including establishments hiring at least one employee. MOEL sampled approximately 3,000 to 6,000 establishments every year prior to 2006, and beginning in 2006 around 32,000 establishments were included. From each sampled establishment, a certain number of employees were randomly selected: all employees for establishments with 5-99 employees, 80 percent for establishments with 100-299, 70 percent for establishments with 300-499, 50 percent for establishments with 500-999, 30 percent for establishments with 1,000-4,999, 20 percent for establishments with 5,000-9,999, and ten percent for establishments with 10,000 or more employees.

Our measure of earnings includes regular pay, overtime pay, and bonuses. Our measure of hourly wages is calculated by dividing contractual monthly wages by the non-overtime hours worked during the month of June. Monthly wages consist of regular pay per month that does not include bonuses or overtime pay. Occupational categories are from Statistics Korea's Korean Standard

Classification of Occupations (KSCO), which is based on International Labor Organization's (ILO) ISCO. The survey provides information on employee gender, age, and education. Education refers to each individual's highest level of educational qualifications at the time of survey and is measured using five categories. Prior to 2002, the categories were: 1) elementary school; 2) middle school; 3) high school; 4) 2-year college; and 5) 4-year college or more. Post-2002 the categories were: 1) less than high school; 2) high school; 3) 2-year college; 4) 4-year college; and 5) graduate school.

The data allow us to examine how gender differences have changed between 1982 and 2012. Supplementary Table 25 and Supplementary Figure 15 present the trends in the Korean gender gap overall, as well as within establishments, occupations, and jobs. Supplementary Table 25 also reports results from models estimated using a consistent sample of gender-integrated jobs in each year. Data similar to those used for this project can be provided by Statistics Korea upon approval of a data application by the relevant authorities.

**Supplementary Table 25.** South Korean Trends in Gender Differences in Earnings

| | All Workplaces | | | | Gender-integrated Jobs Only | | | |
|---|---|---|---|---|---|---|---|---|
| | Basic Adj. | Fixed Effect for: | | | Basic Adj. | Fixed Effect for: | | |
| Year | | Est | Occ | Occ-Est | | Est | Occ | Occ-Est |
| 1982 | -.682 | -.565 | -.524 | -.401 | -.478 | -.411 | -.449 | -.401 |
| 1985 | -.614 | -.545 | -.474 | -.354 | -.480 | -.392 | -.424 | -.354 |
| 1986 | -.585 | -.482 | -.438 | -.326 | -.425 | -.335 | -.385 | -.326 |
| 1987 | -.565 | -.469 | -.440 | -.313 | -.440 | -.326 | -.390 | -.313 |
| 1989 | -.529 | -.427 | -.419 | -.279 | -.412 | -.293 | -.361 | -.279 |
| 1990 | -.523 | -.423 | -.421 | -.272 | -.423 | -.290 | -.363 | -.272 |
| 1992 | -.522 | -.405 | -.413 | -.276 | -.416 | -.291 | -.367 | -.276 |
| 1993 | -.527 | -.410 | -.503 | -.323 | -.456 | -.336 | -.448 | -.323 |
| 1994 | -.499 | -.394 | -.490 | -.320 | -.445 | -.333 | -.447 | -.320 |
| 1995 | -.479 | -.383 | -.482 | -.304 | -.429 | -.310 | -.427 | -.304 |
| 1996 | -.463 | -.373 | -.457 | -.269 | -.391 | -.280 | -.402 | -.269 |
| 1997 | -.458 | -.360 | -.437 | -.255 | -.391 | -.268 | -.390 | -.255 |
| 1998 | -.417 | -.339 | -.412 | -.234 | -.323 | -.246 | -.350 | -.234 |
| 1999 | -.432 | -.325 | -.390 | -.224 | -.359 | -.235 | -.338 | -.224 |
| 2000 | -.405 | -.331 | -.352 | -.227 | -.353 | -.247 | -.319 | -.227 |
| 2001 | -.450 | -.316 | -.387 | -.194 | -.372 | -.212 | -.332 | -.194 |
| 2002 | -.436 | -.355 | -.401 | -.226 | -.367 | -.254 | -.357 | -.226 |
| 2003 | -.430 | -.337 | -.383 | -.208 | -.380 | -.242 | -.334 | -.208 |
| 2004 | -.438 | -.337 | -.387 | -.206 | -.379 | -.245 | -.335 | -.206 |
| 2006 | -.423 | -.306 | -.405 | -.203 | -.353 | -.225 | -.345 | -.203 |
| 2007 | -.423 | -.295 | -.395 | -.205 | -.360 | -.226 | -.344 | -.205 |
| 2008 | -.423 | -.294 | -.378 | -.203 | -.362 | -.217 | -.331 | -.203 |
| 2009 | -.416 | -.304 | -.362 | -.236 | -.365 | -.253 | -.331 | -.236 |

| | | | | | | | | |
|---|---|---|---|---|---|---|---|---|
| 2010 | -.417 | -.292 | -.353 | -.217 | -.350 | -.236 | -.317 | -.217 |
| 2011 | -.423 | -.270 | -.352 | -.205 | -.347 | -.225 | -.307 | -.205 |
| 2012 | -.406 | -.244 | -.335 | -.188 | -.322 | -.198 | -.292 | -.188 |

**Supplementary Figure 15.** Trends in South Korean Gender Differences in Earnings

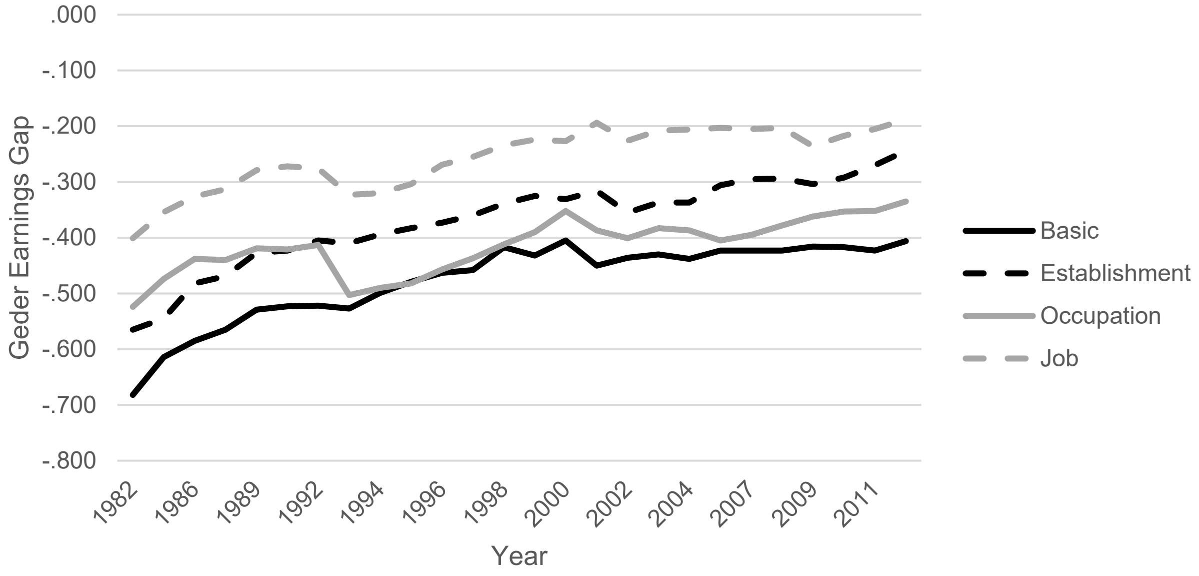


***Spain***. Our analyses use data from the Continuous Sample of Working Histories (CSWH) (*Muestra Continua de Vidas Laborales con Datos Fiscales*) from Spain's Social Security Office. The CSWH contains matched anonymized social security, income tax, and census records for a four percent, non-stratified random sample of the population that had any relationship with Spain's Social Security (whether via employment, self-employment, unemployment or retirement) in that year. The CSWH provides information on individuals' complete labor market histories from 1980 (or the year the individual registers with Social Security) to the year of data collection. Because earnings information from the Social Security records is censored at both the top and bottom of the earnings distribution, we use earnings from tax records containing uncensored gross labor earnings for each job, which are available from 2006 onwards.

For individuals who work at multiple establishments in a year, we only consider the main job, which is either the job with the longest spell within the same firm or the job with the highest earnings across firms. In this way, we build a yearly panel that covers employment spells, with a start/end date and tied to a firm identifier. Each spell includes information on individuals (e.g. age, gender, full-time status), establishments, occupations, and sectors.

Sector is measured using the National Classification of Economic Activities (CNAE-93). The main economic activity of each establishment is captured by one of 59 two-digit sector codes. As the CNAE was modified in 2009, sector codes (CNAE 2009) from 2009 and later have been matched with CNAE-93 equivalents.

As discussed above, our data come from a non-stratified random sample of individuals, so that the gender-integrated establishments and occupation-establishment units in our data may not reflect the population of gender-integrated establishments and occupation-establishment units. To address this, we use weights that ensure that the distribution of women (and separately, the distribution of men) across sectors in our sample matches their distribution in the population. Thus, to the degree that certain sectors tend to have establishments with fewer women (or men) in them, our weights will correct for this. Importantly, however, we do not have information about the number of women and men in each establishment, so we are unable to include this information in our weights. This means that we cannot tell whether the distribution across industries in the overall population matches the distribution across industries in the population of gender-integrated establishments; to the degree that these populations diverge, our weights may introduce bias, as they weight the gender-integrated sample's sectoral distribution to match the overall population's sectoral distribution.

In order to get a robust estimate of the gender distribution across sectors for creating our weights, we pool across all years of the Labor Force Surveys from 2006 to 2017. By taking into consideration the sectoral breakdown of more than 224 million person-years in the Labor Force Surveys (2006-2017), we have calculated a weight for each 2-digit sector code. Finally, we set the maximum weight to 40 in order to avoid the overrepresentation of sectors with weights that are unreasonably large. This upper cap has not been applied to more than one percent of the observations in any year.

To ensure that jobs with unreasonably low earnings are not included in the analyses, we first dropped any observation with earnings below the mean wage of the bottom decile. Second, we integrate information on the minimum wage for each year within our time frame, and for full-time workers we dropped observations earning less than half the minimum wage; for part-time workers, we dropped observations earning less than 25 percent of the minimum wage for any specific year (in Spain the minimum wage for part-time workers is half of the regular minimum wage). In addition to the uncensored aggregate earnings, we calculate hourly earnings (our proxy for hourly wages) for our sample. We calculate hours worked using information on the number of days worked and the percent of employment (e.g., eight hours per day for a full-time worker, four hours per day for a half-time worker, two hours per day for a quarter-time worker).

Our measure of occupation comes from the occupation information that employers are required to provide (*grupo de cotización*) to the Social Security office, and contains ten occupational categories. We use four categories of education: 1) less than secondary education, 2) secondary education, 3) tertiary education, and 4) Masters degrees and above.

Our data allow us to examine how gender differences have changed from 2006 to 2017. Supplementary Table 26 and Supplementary Figure 16 present information on the trends in the Spanish gender gap overall, as well as within establishments, occupations, and jobs. Supplementary Table 26 also reports results from models estimated using a consistent sample of gender-integrated jobs in each year. The variable definitions and information used for weighting are publicly available and can be accessed through the websites of the Social Security Office (*Instituto Nacional De La Seguridad Social*) and Spanish Statistical Office (*Instituto Nacional de Estadística*), respectively. The data files used for this project can be accessed from the Social Security Office upon receipt of authorizations from the Ministry of Labour, Migrations and Social Security of Spain (*Ministerio de Trabajo, Migraciones y Seguridad Social*).

**Supplementary Table 26.** Spanish Trends in Gender Differences in Earnings

| | All Workplaces | | | | Gender-integrated Jobs Only | | | |
|---|---|---|---|---|---|---|---|---|
| | Basic Adj. | Fixed Effect for: | | | Basic Adj. | Fixed Effect for: | | |
| Year | | Est | Occ | Occ-Est | | Est | Occ | Occ-Est |
| 2006 | -.280 | -.264 | -.279 | -.180 | -.322 | -.219 | -.275 | -.180 |
| 2007 | -.266 | -.254 | -.266 | -.179 | -.310 | -.219 | -.267 | -.179 |
| 2008 | -.230 | -.237 | -.235 | -.158 | -.285 | -.198 | -.244 | -.158 |
| 2009 | -.176 | -.210 | -.181 | -.139 | -.249 | -.179 | -.208 | -.139 |
| 2010 | -.172 | -.198 | -.173 | -.134 | -.240 | -.171 | -.201 | -.134 |
| 2011 | -.157 | -.189 | -.159 | -.121 | -.233 | -.155 | -.191 | -.121 |
| 2012 | -.142 | -.188 | -.147 | -.126 | -.230 | -.159 | -.195 | -.126 |
| 2013 | -.114 | -.166 | -.121 | -.113 | -.190 | -.142 | -.158 | -.113 |
| 2014 | -.118 | -.169 | -.128 | -.116 | -.187 | -.147 | -.157 | -.116 |
| 2015 | -.124 | -.166 | -.136 | -.115 | -.186 | -.143 | -.159 | -.115 |
| 2016 | -.140 | -.172 | -.149 | -.120 | -.199 | -.148 | -.167 | -.120 |
| 2017 | -.158 | -.176 | -.164 | -.121 | -.204 | -.150 | -.174 | -.121 |

**Supplementary Figure 16.** Trends in Spanish Gender Differences in Earnings

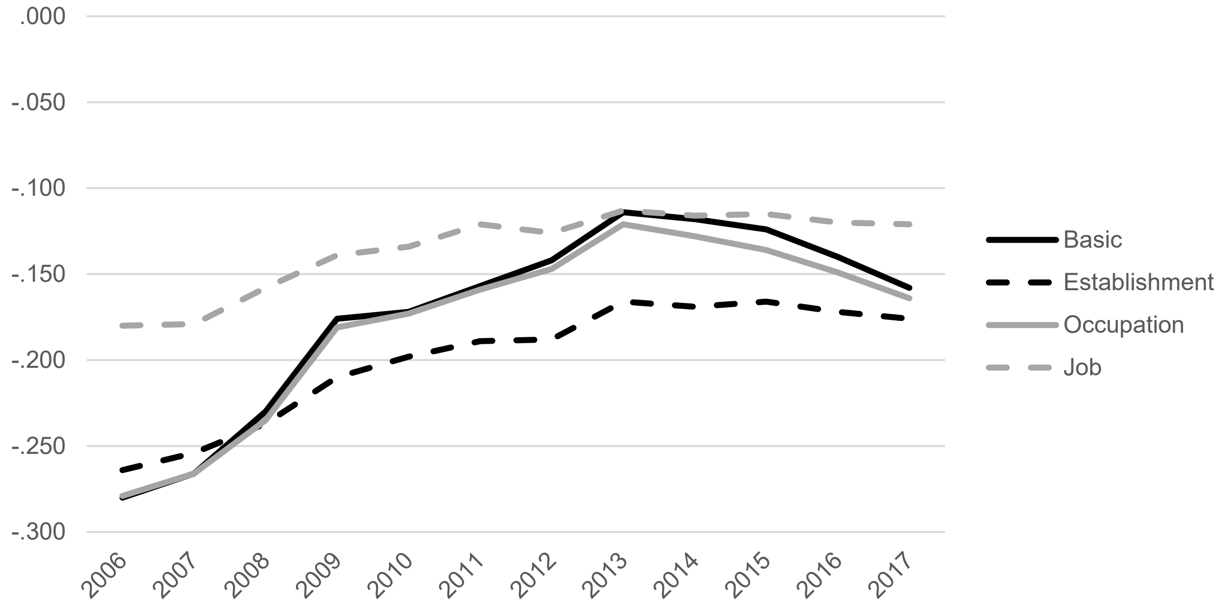


***Sweden***. Our analyses use data from Statistics Sweden's wage statistics in the period 2004-2018 for information on contractual monthly salaries, contractual hours worked (which is used to compute part- versus full-time status), and identifying employers. These data are collected once per year in the fall (with some variation across sectors) on all job observations in the public sector and in large private firms (500+ employees), and for a representative sample for the rest of the private sector. Taken together, these data include information on all public employees and roughly half of all workers in the private sector. In the private sector sample, the sampling unit is the firm and all establishments within a firm are grouped together as one employer unit. This sample covers approximately five percent of establishments in all firms, but very small firms (one through nine employees) are underrepresented (we have information on roughly three percent of establishments in very small firms). All individuals in each sampled firm are included in the data. The private sector sample is stratified by industry, white vs. blue collar work, and number of employees.

Our measure of hourly wages is based on information on contractual monthly salaries and contractual hours worked at the time of registration each year. Monthly salary information is based on contractual regular earnings per month and does not include non-contractual bonuses, non-regular extra pay, or overtime pay. In the private sector, hours worked is based on information on contractual hours worked per week. In the public sector, hours worked is based on information on the percent of full-time hours of employment (i.e., it measures the individual's contractual work hours as the percent of regular full-time work, ranging between zero and 100). The measure of earnings comes from tax records, and includes all work-related income (such as parental and sick leave benefits; but not unemployment benefits) for each year. Although earnings data exist for the full

population, we merge these to the wage statistics sample in order to get information on contractual work hours to create our indicator of full- versus part-time work.

Occupation is based on Statistics Sweden's Swedish version of ISCO-88 (*Standard för svensk yrkesklassificering, SSYK96*). These are available at the three- to four-digit level since the mid-1990s, and at the four-digit level since 2004. For individuals who work multiple jobs and thus have multiple job observations per year, we use information from their job observation with the highest contractual monthly salary.

Information about gender and age is based on records from the national register. Information about education refers to each individual's highest level of educational qualifications in each year based on annual records from the Education Register, using the Swedish version of ISCED-97. We use this information to create a 16-category measure that distinguishes between levels of education (e.g., secondary vs. tertiary) and type of education within-level (e.g., vocational vs. academic/general).

Our data allow us to examine how gender differences have changed from 2004-2018. Supplementary Table 27 and Supplementary Figure 17 present information on the trends in the Swedish gender gap overall, as well as within establishments, occupations, and jobs. In Supplementary Table 28 we report trends from models on earnings estimated on the whole population, not limited to the wage statistics sample (but where we lack information on which workers were full- versus part-time, and so use an earnings-based proxy for this). These results also use 3-digit occupation codes, as Statistics Sweden restricts the level of detail available on ISCO codes for the full population. Given the salience of full- versus part-time work in the Swedish context, we use results from our sample-based analyses as our primary specification. Supplementary Tables 27 and 28 also report result from models estimated using a consistent sample of gender-integrated jobs in each year. Data similar to those used for this project can be accessed at Statistics Sweden upon receipt of the proper authorizations.

**Supplementary Table 27.** Swedish Trends in Gender Differences in Earnings

| | All Workplaces | | | | Gender-integrated Jobs Only | | | |
|---|---|---|---|---|---|---|---|---|
| | Basic Adj. | Fixed Effect for: | | | Basic Adj. | Fixed Effect for: | | |
| Year | | Est | Occ | Occ-Est | | Est | Occ | Occ-Est |
| 2004 | -.201 | -.164 | -.126 | -.115 | -.182 | -.145 | -.130 | -.115 |
| 2005 | -.207 | -.164 | -.127 | -.113 | -.184 | -.142 | -.129 | -.113 |
| 2006 | -.207 | -.160 | -.122 | -.108 | -.183 | -.140 | -.125 | -.108 |
| 2007 | -.213 | -.161 | -.124 | -.109 | -.189 | -.141 | -.125 | -.109 |
| 2008 | -.208 | -.159 | -.124 | -.108 | -.182 | -.137 | -.125 | -.108 |
| 2009 | -.193 | -.145 | -.118 | -.101 | -.172 | -.126 | -.119 | -.101 |
| 2010 | -.194 | -.143 | -.116 | -.097 | -.170 | -.124 | -.116 | -.097 |

| | | | | | | | | |
|---|---|---|---|---|---|---|---|---|
| 2011 | -.195 | -.142 | -.117 | -.097 | -.172 | -.124 | -.116 | -.097 |
| 2012 | -.195 | -.138 | -.116 | -.095 | -.170 | -.119 | -.112 | -.095 |
| 2013 | -.191 | -.133 | -.112 | -.089 | -.162 | -.113 | -.107 | -.089 |
| 2014 | -.189 | -.130 | -.100 | -.081 | -.158 | -.109 | -.096 | -.081 |
| 2015 | -.183 | -.130 | -.098 | -.081 | -.157 | -.110 | -.096 | -.081 |
| 2016 | -.179 | -.124 | -.094 | -.077 | -.150 | -.104 | -.090 | -.077 |
| 2017 | -.174 | -.118 | -.089 | -.073 | -.146 | -.099 | -.088 | -.073 |
| 2018 | -.175 | -.118 | -.093 | -.076 | -.144 | -.100 | -.089 | -.076 |

**Supplementary Table 28.** Swedish Trends in Gender Differences in Earnings using Whole Labor Market LISA Data

| | All Workplaces | | | | Gender-integrated Jobs Only | | | |
|---|---|---|---|---|---|---|---|---|
| | Basic Adj. | Fixed Effect for: | | | Basic Adj. | Fixed Effect for: | | |
| Year | | Est | Occ | Occ-Est | | Est | Occ | Occ-Est |
| 2001 | -.279 | -.218 | -.190 | -.160 | -.262 | -.183 | -.190 | -.160 |
| 2002 | -.272 | -.214 | -.187 | -.158 | -.254 | -.181 | -.188 | -.158 |
| 2003 | -.266 | -.208 | -.182 | -.156 | -.251 | -.178 | -.185 | -.156 |
| 2004 | -.265 | -.205 | -.179 | -.155 | -.253 | -.176 | -.183 | -.155 |
| 2005 | -.271 | -.204 | -.178 | -.153 | -.257 | -.174 | -.181 | -.153 |
| 2006 | -.269 | -.198 | -.174 | -.149 | -.255 | -.170 | -.177 | -.149 |
| 2007 | -.271 | -.193 | -.173 | -.145 | -.255 | -.165 | -.174 | -.145 |
| 2008 | -.264 | -.187 | -.170 | -.141 | -.247 | -.161 | -.171 | -.141 |
| 2009 | -.248 | -.180 | -.161 | -.136 | -.232 | -.155 | -.163 | -.136 |
| 2010 | -.248 | -.178 | -.160 | -.134 | -.233 | -.153 | -.161 | -.134 |
| 2011 | -.249 | -.173 | -.159 | -.131 | -.233 | -.149 | -.159 | -.131 |
| 2012 | -.245 | -.166 | -.154 | -.125 | -.228 | -.143 | -.154 | -.125 |

**Supplementary Figure 17.** Trends in Swedish Gender Differences in Earnings

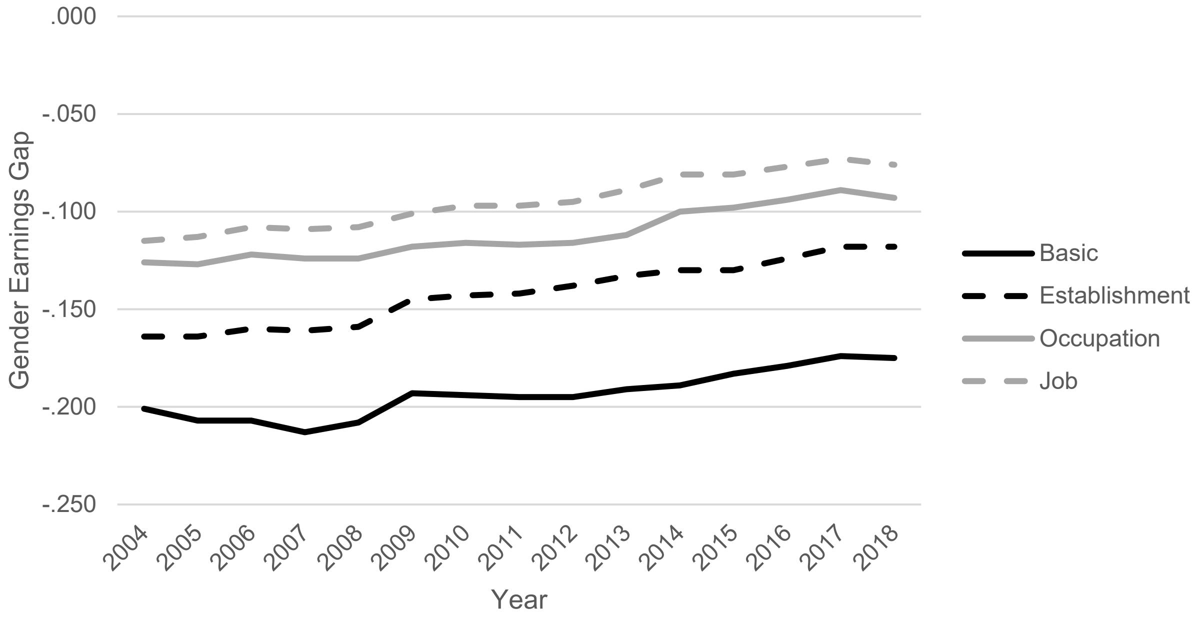


***United States***. Our analyses use earnings and employer information for each individual's employment spell(s) from Internal Revenue Service (IRS) Form W-2, and cover the tax years 2005-2015. Individuals on this form are identified and linked across datasets using a unique, anonymized Personal Identification Key (PIK). This form also contains the Employer Identification Number (EIN), which in most cases identifies a firm (see *11* for more details). Because we lack geographic information on Form W-2, we are unfortunately unable to stratify further by region or state. We take Box 1 from W-2, which reports total annual taxable earnings for each individual at a particular EIN, including salary, wages, and bonuses, but excludes deferred compensation. W-2 reports do not indicate spell duration or the number of hours worked. We unduplicate by EIN-PIK-year, taking the most recently dated form available. For individuals who work at multiple EINs in a year, we use information from their highest-earning W-2 report, selecting one at random in the case of individuals with multiple equally well-compensated W-2s. Supplemental analyses using all unduplicated W-2 employment spells (instead of only the highest-paid spell) provide similar results.

Because Form W-2 contains no occupational information, we link these forms to the American Community Survey (ACS), a one percent random sample of U.S. households that asks respondents to self-report their current primary or most recent primary occupation at the time of the survey. We link individuals' highest-paid W-2 report to the concurrent ACS year; for example, W-2s from tax year 2015 are linked to respondents in the 2015 ACS. Self-reported occupations are coded by highly-trained Census Bureau coders into one of approximately 500 three-digit categories from

the Standard Occupation Classification (SOC) system. Analyses using less granular two-digit occupational codes produce similar patterns, suggesting that changes in this classification system does not affect results. Information about gender and age come from the Social Security Administration's Numerical Identification File (Numident).

We additionally derive information on hours worked, weeks worked, and education from the ACS. Educational information applies to the current period, and (average) hours worked and weeks worked pertain to the previous 12 months. We multiply hours worked by weeks worked (using interval midpoints for weeks worked) to obtain the total annual number of hours worked. We then divide total W-2 earnings by annual hours worked to arrive at our estimate of hourly wage in a typical week. This assumes individuals are working a similar number of hours in the current year. Unfortunately, these data do not allow us to isolate overtime and bonuses from total compensation in creating this hourly wage variable.

We define individuals as working full-time if their total nominal W-2 earnings surpassed the equivalent of working the federal minimum wage in that year × 40 hours × 50 weeks. Similarly, we define marginal part-time workers – those with either very low earnings or who worked very few hours in the year – as earning *less than* equivalent of the minimum wage × 40 hours × 13 weeks. All models control for marginal part-time work, but we do not use the marginal part-time work to define our fixed effects. Analyses using sample self-reported hours worked yield gender earnings gaps that are comparable to our estimates based on earnings thresholds.

Our data allow us to examine how gender differences have changed from 2005-2015. Supplementary Table 29 and Supplementary Figure 18 present information on the trends in the U.S. gender gap overall, as well as within establishments, occupations, and jobs. Supplementary Table 30 presents the total annual gender earnings gaps from 2005-2015 using the full W2 dataset. In these analyses we restrict to individuals' highest paid W2 spell in a year. The first column in Supplementary Table 30 conditions only on age, age squared, and part-time work, while the second additionally conditions on firm fixed effects. Data used for this project can be accessed at the U.S. Census Bureau upon receipt of proper authorizations.

**Supplementary Table 29.** US Trends in Gender Differences in Earnings

| Year | Basic Adj. | Fixed Effect for: | | |
|---|---|---|---|---|
| | | Est | Occ | Occ-Est |
| 2005 | -.332 | -.255 | -.234 | -.165 |
| 2006 | -.334 | -.254 | -.236 | -.163 |
| 2007 | -.321 | -.245 | -.226 | -.159 |
| 2008 | -.302 | -.235 | -.213 | -.155 |
| 2009 | -.275 | -.219 | -.197 | -.142 |
| 2010 | -.279 | -.215 | -.198 | -.140 |
| 2011 | -.282 | -.209 | -.195 | -.134 |

| | | | | |
|---|---|---|---|---|
| 2012 | -.285 | -.209 | -.196 | -.135 |
| 2013 | -.286 | -.211 | -.195 | -.136 |
| 2014 | -.290 | -.210 | -.199 | -.135 |
| 2015 | -.296 | -.214 | -.202 | -.141 |

**Supplementary Table 30.** US Trends in Gender Differences in Earnings using Full W2 Sample

| Year | Basic Adj. | Firm Fixed Effect |
|---|---|---|
| 2005 | -.255 | -.253 |
| 2006 | -.260 | -.254 |
| 2007 | -.245 | -.241 |
| 2008 | -.228 | -.227 |
| 2009 | -.199 | -.206 |
| 2010 | -.203 | -.203 |
| 2011 | -.206 | -.203 |
| 2012 | -.207 | -.200 |
| 2013 | -.207 | -.197 |
| 2014 | -.211 | -.196 |
| 2015 | -.212 | -.195 |

**Supplementary Figure 18.** Trends in US Gender Differences in Earnings

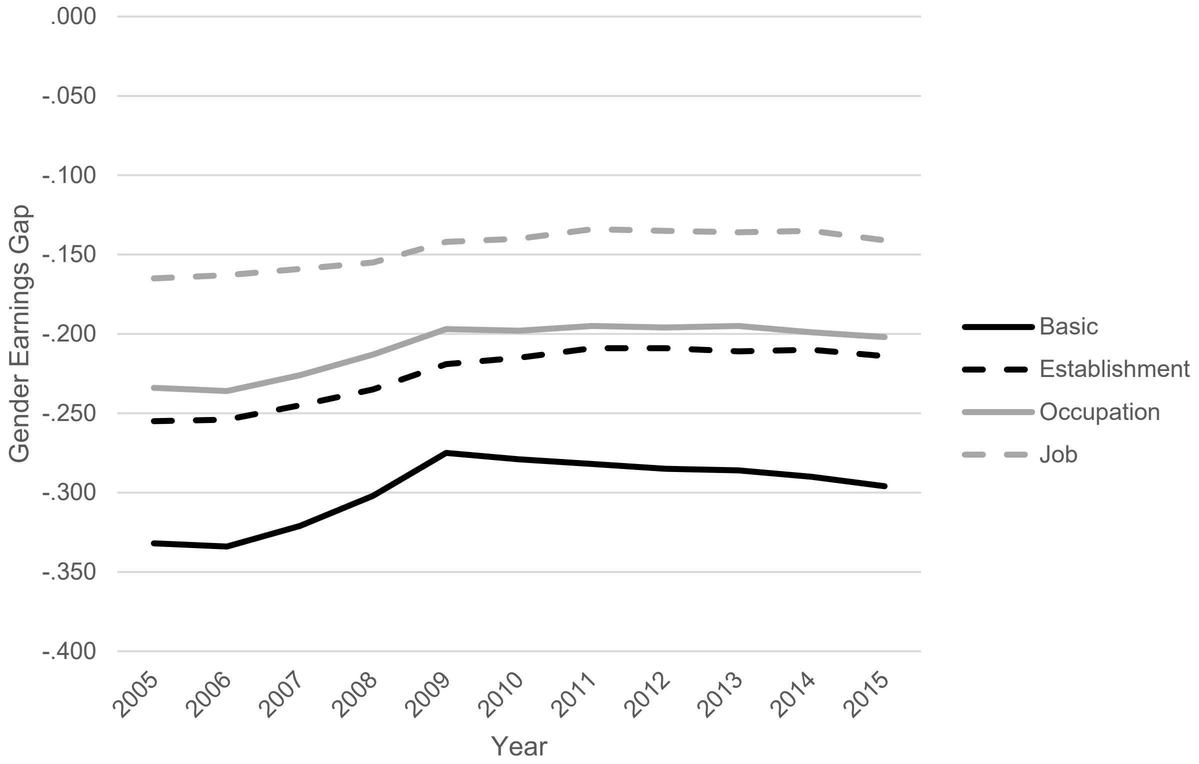

**Supplement References**

1. T. Petersen, L. A. Morgan, Separate and unequal: Occupation-establishment sex segregation and the gender wage gap. Am. J. Soc. 101, 2, 329-367 (1995).
2. E. M. Meyersson Milgrom, T. Petersen, V. Snartland, Equal pay for equal work? Evidence from Sweden and a comparison with Norway and the US. Scand. J. Econ. 103, 4, 559-583 (2001).
3. T. Petersen, V. Snartland, L-E Becken, K. Modesta Olsen, Within-job wage discrimination and the gender wage gap: The case of Norway. Eur. Soc. Rev. 13, 2, 199-213 (1997).
4. K. Bayard, J. Hellerstein, D. Neumark, and K. Troske, New Evidence on Sex Segregation and Sex Differences in Wages from Matched Employee-Employer Data. J. Lab. Econ. 21, 4, 887-922 (2003).
5. B.F. Reskin, H.I. Hartmann. Women's Work, Men's Work: Sex Segregation on the Job. (National Academy Press, 1986).
6. C. Geist, One Germany, two worlds of housework? Examining employed single and partnered women in the decade after unification. *J. Comp. Fam. Stud.* **40, 3**, 415-437 (2009).
7. R.A. Rosenfeld, H. Trappe, J.C. Gornick, Gender and work in Germany: Before and after reunification. *Ann. Rev. Sociol.* **30,** 103-124 (2004).
8. L. Kish, Sampling organizations and groups of unequal sizes. *Am. Soc. Rev.* **30**, 564-572 (1965).
9. D. Card, J. Heining, P. Kline, Workplace heterogeneity and the rise of West German wage inequality. *Quart. J. Econ.* **128, 3**, 967-1015 (2013).
10. D. Tomaskovic-Devey, & S.M. Melzer.. The organizational production of earnings inequalities, Germany 1995–2010. *PloS one*, *15*(9), e0237970 (2020).
11. J. Song, D. J. Price, F. Guvenen, N. Bloom, T. von Wachter. Firming up inequality. *Quart. J. Econ.* **134, 1,** 1-50 (2018).
