# Supplementary material for: Within-job gender pay inequality in 15 countries
Source: Nat Hum Behav. 2022 Nov 24;7(2):184–9. doi: 10.1038/s41562-022-01470-z (PMC9957724; doi:10.1038/s41562-022-01470-z)
Supplement: Supplementary file 2 — Reporting Summary [file 41562_2022_1470_MOESM2_ESM.pdf]

nature research

Corresponding author(s): Andrew Penner

Last updated by author(s): 7/26/2022

Reporting Summary

Nature Research wishes to improve the reproducibility of the work that we publish. This form provides structure for consistency and transparency in reporting. For further information on Nature Research policies, see our Editorial Policies and the Editorial Policy Checklist.

Statistics

For all statistical analyses, confirm that the following items are present in the figure legend, table legend, main text, or Methods section.

n/a	Confirmed	
☒	☐	The exact sample size (*n*) for each experimental group/condition, given as a discrete number and unit of measurement
☒	☐	A statement on whether measurements were taken from distinct samples or whether the same sample was measured repeatedly
☐	☒	The statistical test(s) used AND whether they are one- or two-sided<br>*Only common tests should be described solely by name; describe more complex techniques in the Methods section.*
☐	☒	A description of all covariates tested
☒	☐	A description of any assumptions or corrections, such as tests of normality and adjustment for multiple comparisons
☐	☒	A full description of the statistical parameters including central tendency (e.g. means) or other basic estimates (e.g. regression coefficient) AND variation (e.g. standard deviation) or associated estimates of uncertainty (e.g. confidence intervals)
☐	☒	For null hypothesis testing, the test statistic (e.g. *F*, *t*, *r*) with confidence intervals, effect sizes, degrees of freedom and *P* value noted<br>*Give P values as exact values whenever suitable.*
☒	☐	For Bayesian analysis, information on the choice of priors and Markov chain Monte Carlo settings
☒	☐	For hierarchical and complex designs, identification of the appropriate level for tests and full reporting of outcomes
☒	☐	Estimates of effect sizes (e.g. Cohen's *d*, Pearson's *r*), indicating how they were calculated

Our web collection on statistics for biologists contains articles on many of the points above.

Software and code

Policy information about availability of computer code

Data collection	No software was used to collect the data used in this study.
Data analysis	Data were analyzed in STATA 14-17 and SAS 9.

For manuscripts utilizing custom algorithms or software that are central to the research but not yet described in published literature, software must be made available to editors and reviewers. We strongly encourage code deposition in a community repository (e.g. GitHub). See the Nature Research guidelines for submitting code & software for further information.

Data

Policy information about availability of data

All manuscripts must include a data availability statement. This statement should provide the following information, where applicable:

- Accession codes, unique identifiers, or web links for publicly available datasets
- A list of figures that have associated raw data
- A description of any restrictions on data availability

This paper uses restricted-access data that require approval from Statistics Canada, the Ministry of Labor and Social Affairs of the Czech Republic, Statistics Denmark, the French Comité du Secret Statistique, the German Institute for Employment Research, the Databank of the Centre for Economic and Regional Studies in Hungary, Israel's Central Bureau of Statistics (CBS), the Japanese Ministry of Health, Labour and Welfare, the Central Bureau of Statistics of the Netherlands, Statistics Norway, the Slovenian Statistical Office, Statistics Korea, the Spanish Ministerio de Trabajo, Migraciones y Seguridad Social, Statistics Sweden, and the U.S. Census Bureau.

Field-specific reporting

Please select the one below that is the best fit for your research. If you are not sure, read the appropriate sections before making your selection.

☐ Life sciences ☒ Behavioural & social sciences ☐ Ecological, evolutionary & environmental sciences

For a reference copy of the document with all sections, see nature.com/documents/nr-reporting-summary-flat.pdf

Behavioural & social sciences study design

All studies must disclose on these points even when the disclosure is negative.

Study description	We use quantitative data from 15 countries to examine the level of within-occupation, within-establishment, and within-job gender pay differences.
Research sample	This study used existing datasets from Statistics Canada, the Ministry of Labor and Social Affairs of the Czech Republic, Statistics Denmark, the French Comité du Secret Statistique, the German Institute for Employment Research, the Databank of the Institute of Economics (Center for Economic and Regional Studies) of the Hungarian Academy of Sciences, Israel's Central Bureau of Statistics (CBS), the Japanese Ministry of Health, Labour and Welfare, the Central Bureau of Statistics of the Netherlands, Statistics Norway, the Slovenian Statistical Office, Statistics Korea, the Spanish Ministerio de Trabajo, Migraciones y Seguridad Social, Statistics Sweden, and the U.S. Census Bureau. These datasets were chosen to allow for between women and men who are working in the same occupation and establishments.
Sampling strategy	No data were collected for this study.
Data collection	No data were collected for this study.
Timing	No data were collected for this study.
Data exclusions	No valid data were excluded; see country specific descriptions of data cleaning.
Non-participation	No participants were recruited for this study.
Randomization	This is a descriptive study comparing women and men, and as such we did not randomize.

Reporting for specific materials, systems and methods

We require information from authors about some types of materials, experimental systems and methods used in many studies. Here, indicate whether each material, system or method listed is relevant to your study. If you are not sure if a list item applies to your research, read the appropriate section before selecting a response.

Materials & experimental systems

n/a	Involved in the study	
☒	☐	Antibodies
☒	☐	Eukaryotic cell lines
☒	☐	Palaeontology and archaeology
☒	☐	Animals and other organisms
☒	☐	Human research participants
☒	☐	Clinical data
☒	☐	Dual use research of concern

Methods

n/a	Involved in the study	
☒	☐	ChIP-seq
☒	☐	Flow cytometry
☒	☐	MRI-based neuroimaging
